# Supplementary material for: simPIC: flexible simulation of single-cell ATAC-seq paired-insertion counts from individuals to populations
Source: Genome Biol. 2026 Jul 29;27:325. doi: 10.1186/s13059-026-04199-4 (PMC13700425; doi:10.1186/s13059-026-04199-4)
Supplement: Supplementary file 1 [file 13059_2026_4199_MOESM1_ESM.pdf]

Supplementary information for simPIC

Sagrika Chugh[1,2], Heejung Shim[2,3,4], Davis J. McCarthy[1,2,5*]

[1]Bioinformatics and Cellular Genomics, St. Vincent's Institute of Medical Research, 9 Princes Street, Fitzroy, 3065, Victoria, Australia.
[2]School of Mathematics and Statistics, Faculty of Science, University of Melbourne, Grattan Street, Parkville, 3010, Victoria, Australia.
[3]Snow Vision Accelerator, Sydney Precision Data Science Centre, School of Mathematics and Statistics, University of Sydney, Sydney, New South Wales, Australia.
[4]Melbourne Integrative Genomics, ARC Centre of Excellence for the Mathematical Analysis of Cellular Systems, School of Mathematics and Statistics, University of Melbourne, Parkville, 3010, Victoria, Australia.
[5]Faculty of Medicine, Dentistry and Health Sciences, University of Melbourne, Grattan Street, Parkville, 3010, Victoria, Australia.

*Corresponding author(s). E-mail(s): dmccarthy@svi.edu.au;
Contributing authors: schugh@svi.edu.au;
heejung.shim@unimelb.edu.au;

Table S1: Input parameters for simPIC simulation model.

Category	Parameter	Symbol	Description	Source
Single-cell Parameters	Mean scale and shape	η, κ	Scale and shape parameters for the Weibull distribution used to model peak means (λ_i).	estimated from input data
Single-cell Parameters	Library size location and scale	μ, σ	Parameters for the log-normal distribution characterizing the library size (L_j).	estimated from input data
Single-cell Parameters	Sparsity	π_j	The proportion of zeros in the peak-by-cell matrix, modeled via a Bernoulli distribution ($Z_{i,j}$).	estimated from input data
Single-cell Parameters	Dispersion & Degrees of Freedom	ϕ, df_0	Common dispersion and degrees of freedom used to calculate the Biological Coefficient of Variation (BCV).	estimated from input data

Continued on next page

**Category**	**Parameter**	**Symbol**	**Description**	**Source**
Population Parameters	Population mean shape and rate	η_p, κ_p	Weibull distribution parameters for generating peak means across a population.	estimated from input data
Population Parameters	Variance shape and rate	α_v, β_v	Gamma distribution parameters governing the coefficient of variation (σ_i) for sample-specific means.	estimated from input data
Population Parameters	caQTL effect shape and rate	α_c, β_c	Gamma distribution parameters for the chromatin accessibility QTL effect size (ω_i).	estimated from input data
Manual & Batch Parameters	Batch factor location and scale	μ_b, σ_b	Log-normal parameters defining the batch effect (ω_j^b) applied to cell means.	user defined; default provided
Manual & Batch Parameters	Group DA effect	$\pi_{da}, \mu_{da}, \sigma_{da}$	Probability, location, and scale parameters for defining group-specific differentially accessible (DA) effects.	user-defined; default provided
Manual & Batch Parameters	Similarity scale	S_s	A manual scaling factor influencing the population-wide variance.	user-defined; default provided
Manual & Batch Parameters	caQTL group specific	$caQTL_g$	The percentage of caQTLs that are assigned as specific to a particular group.	user-defined; default provided

Table S2 Usability and practical comparison of scATAC-seq simulators.

Dimension	simPIC	scDesign3	scMultiSim	scReadSim	DiTSim
Required input	**Count matrix only**	Count matrix + cell annotations	Count matrix + GRN specification	BAM file + reference genome	Count matrix + cell annotations
Parameter estimation	**Automatic** `simPICestimate()`	Automatic	Manual / guided	Automatic from BAM	Deep-model training
Setup complexity	**Low**	Medium	High	High; BAM-processing pipeline	Medium–high; GPU recommended
Computational cost	**Low**	Moderate	High	High	High; deep learning
Bioconductor / CRAN	**Bioconductor**	GitHub / CRAN	Bioconductor	PyPI; Python	GitHub; Python
Interpretable parameters	✓	✓	Partial	✗	✗; black box
Diagnostic / QC tools	✓ `simPICcompare()`	✓	✗	✗	✗
Primary benchmarking use case	**Count-level analysis; population genetics / caQTL studies**	Multi-modal benchmarking	GRN, CCI and RNA velocity benchmarking	Read-level benchmarking; peak callers and UMI deduplication	Clustering and annotation
Population-scale capability	✓ `splatPop` integration	✗	✗	✗	✗
Accessible to R-only users	✓	✓	✓	✗	✗

Key: ✓ = supported; ✗ = not supported. Green ticks indicate supported capabilities; red crosses indicate absent capabilities. GRN, gene regulatory network; CCI, cell–cell interaction; caQTL, chromatin accessibility quantitative trait locus; UMI, unique molecular identifier.

Table S3 Feature and scope comparison of scATAC-seq simulators.

Feature	simPIC	scDesign3	scMultiSim	scReadSim	DiTSim
Modality focus	**scATAC-seq only**	Multi-modal; RNA, ATAC, spatial, CITE	Multi-modal; RNA, ATAC, velocity, spatial	scRNA-seq + scATAC-seq; read-level	scATAC-seq only
Output type	**Count matrix; peak × cell**	Count matrix	Count matrix	Raw reads; FASTQ / BAM	Count matrix; peak × cell
Statistical/modelling framework	**Gamma–Poisson / Weibull**	GAMLSS / copula	Kinetic ODE / GRN	scDesign2/3 internally	Diffusion transformer; deep learning
PIC-based quantification	✓	✗	✗	✗	✗
Multiple cell types	✓	✓	✓	✓	✓
Continuous trajectories	✗	✓	✓	✓; via scDesign3	✗
Batch effects	✓	✓	✓	✓	✗
Population-scale; multi-individual	**✓; via `splatPop`**	✗	✗	✗	✗
caQTL / genetic effects	✓	✗	✗	✗	✗
GRN-guided simulation	✗	✗	✓	✗	✗
Requires cell type annotations	✗	✓	✓; cell states	✗	✓
Requires bulk ATAC data	✗	✗	✗	✗	✗
Sparsity modelling	**✓; Bernoulli**	✓	Partial	✓	✓
Read-level output; FASTQ/BAM	✗	✗	✗	✓	✗
Diagnostic / QC tools built in	**✓; `simPICcompare`**	✓	✗	✗	✗
Language	**R**	R	R	Python	Python
Bioconductor native	✓	✗	✓	✗	✗

Abbreviations: PIC, Paired Insertion Counting; GRN, gene regulatory network; caQTL, chromatin accessibility quantitative trait locus; CCI, cell–cell interaction; ODE, ordinary differential equation; CITE, cellular indexing of transcriptomes and epitopes by sequencing.

Table S4: Cell types included in the simulator benchmark. For each dataset, the table reports the annotated cell type, number of cells, total number of peaks before filtering, and number of peaks retained after applying the 1% peak-detection filter.

Dataset	Celltype	nCells	nPeaks	nPeaks (1%filter)
PBMC5k	CD14 Monocytes	1768	69523	56279
	CD4 Naive	432	69523	45140
	CD8 Effector	505	69523	51493
	Double negative T cell	286	69523	46534
	CD4 Memory	641	69523	47538
	CD8 Naive	278	69523	43410
PBMC10k	CD14 Monocytes	2201	127689	106967
	CD4 Memory	2419	127689	83919
	CD4 Naive	1033	127689	78962
	CD8 Effector	866	127689	81813
	B cell progenitor	203	165434	97785
	CD8 naïve	440	165434	84291
	NK dim	760	165434	91594
Satpathy	CD 34 Progenitors	14633	571400	175151
	Monocytes	1868	571400	144533
Buquicchio	Liver TRM	4618	67364	64428
Fly Brain	OL Type I NB	323	51135	42926
	OL Neuroepithelium	274	51135	49296
	Capa	216	51135	42196
	CB Type I NB	205	51135	43759
Cusanovich (Spleen)	B cells	2197	73177	62219
Cusanovich (Cerebellum)	Cerebellar granule cells	1091	67260	37376
	Astrocytes	363	67260	36496
Cusanovich (Kidney)	Podocyte	447	101900	40426
	Proximal Tubule	2565	101900	85796

Table S5 Summary statistics for real and simulated data
(a) Peak usage and absolute value percentiles

Cell Type	Transform	Peaks Used		P95 Abs		P99 Abs	
		Real	Sim	Real	Sim	Real	Sim
PBMC10k_CD8_effector	TFIDF	2000	2000	0.080	0.070	0.112	0.092
PBMC5k_CD4_memory	TFIDF	2000	2000	0.312	0.187	0.737	0.455
Cusanovich_Podocytes	TFIDF	2000	2000	0.204	0.438	0.426	0.780
Satpathy_Monocytes	TFIDF	2000	2000	0.163	0.050	0.384	0.070
FlyBrain_OL_neuroepi	TFIDF	2000	2000	0.162	0.632	0.273	0.829
Liver_TRM	TFIDF	2000	2000	0.078	0.034	0.391	0.049

(b) Pair counts and correlation thresholds

Cell Type	Transform	N Pairs		Abs $r > 0.3$		Abs $r > 0.5$	
		Real	Sim	Real	Sim	Real	Sim
PBMC10k_CD8_effector	TFIDF	1999000	1999000	63	0	6	0
PBMC5k_CD4_memory	TFIDF	1999000	1999000	104445	46953	56421	15093
Cusanovich_Podocytes	TFIDF	1999000	1999000	50800	120320	11225	87219
Satpathy_Monocytes	TFIDF	1999000	1999000	36053	38	9187	1
FlyBrain_OL_neuroepi	TFIDF	1999000	1999000	14322	199140	2012	142129
Liver_TRM	TFIDF	1999000	1999000	35606	26	9718	2

Table S6: Donor-associated separation metrics for real and simulated data across earlyAD and nonAD libraries.

Condition	Library	Type	Mean silhouette	Mean purity
earlyAD	Library11	real	-0.0120	0.5063
		simulated	-0.0002	0.3074
	Library7	real	-0.1261	0.2562
		simulated	-0.0092	0.2229
	Library9	real	-0.0246	0.5943
		simulated	0.0118	0.5653
	Library5	real	0.0051	0.5393
		simulated	0.0171	0.4731
	Library10	real	-0.0581	0.3333
		simulated	-0.0070	0.2640
	Library4	real	0.0247	0.6249
		simulated	0.0118	0.5702
nonAD	Library10	real	-0.0816	0.2570
		simulated	-0.0153	0.1912
	Library7	real	-0.1448	0.2843
		simulated	-0.0139	0.1974
	Library9	real	-0.0320	0.3475
		simulated	-0.0004	0.3018
	Library5	real	0.0728	0.5300
		simulated	0.0114	0.3947
