# Supplementary material for: simPIC: flexible simulation of single-cell ATAC-seq paired-insertion counts from individuals to populations
Source: Genome Biol. 2026 Jul 29;27:325. doi: 10.1186/s13059-026-04199-4 (PMC13700425; doi:10.1186/s13059-026-04199-4)
Supplement: Supplementary file 2 [file 13059_2026_4199_MOESM2_ESM.pdf]

Supplementary information for simPIC

Sagrika Chugh[1,2], Heejung Shim[2,3,4], Davis J. McCarthy[1,2,5*]

[1]Bioinformatics and Cellular Genomics, St. Vincent's Institute of Medical Research, 9 Princes Street, Fitzroy, 3065, Victoria, Australia.
[2]School of Mathematics and Statistics, Faculty of Science, University of Melbourne, Grattan Street, Parkville, 3010, Victoria, Australia.
[3]Snow Vision Accelerator, Sydney Precision Data Science Centre, School of Mathematics and Statistics, University of Sydney, Sydney, New South Wales, Australia.
[4]Melbourne Integrative Genomics, ARC Centre of Excellence for the Mathematical Analysis of Cellular Systems, School of Mathematics and Statistics, University of Melbourne, Parkville, 3010, Victoria, Australia.
[5]Faculty of Medicine, Dentistry and Health Sciences, University of Melbourne, Grattan Street, Parkville, 3010, Victoria, Australia.

*Corresponding author(s). E-mail(s): dmccarthy@svi.edu.au;
Contributing authors: schugh@svi.edu.au;
heejung.shim@unimelb.edu.au;

Supplementary Note 1: Distribution for modelling peak means

We provide four distributions for simulating peak means (λ), namely Weibull, gamma and lognormal-gamma mixture(default), and Pareto.

The probability density function (PDF) of the lognormal-gamma mixture model is given by:

$$f_{\text{lngamma}}(x|\alpha, \beta, \mu, \sigma^2) = \pi \cdot f_{\text{Gamma}}(x|\alpha, \beta) + (1 - \pi) \cdot f_{\text{Lognormal}}(x|\mu, \sigma^2) \quad (1)$$

where π is the proportion of non-zero elements/counts ($0 \leq \pi \leq 1$), α and β are the shape and rate parameter of the gamma distribution respectively,μ and σ^2 are the mean and variance of the lognormal distribution,

The lognormal-gamma mixture model is particularly useful when the data exhibit characteristics that could be modeled by both a gamma distribution (for positive

skewness and flexibility in shape) and a lognormal distribution (for right-skewed data where values are strictly positive and logarithmic transformations are appropriate).

Supplementary Note 2: Mathematical Derivation of BCV and Trended Means

The biological coefficient of variation (BCV) for each peak i in cell j ($B_{i,j}$) is simulated to reflect the mean–variance trend observed in scATAC-seq data. Using the common dispersion ϕ and prior degrees of freedom df_0 estimated via *edgeR*, $B_{i,j}$ is derived as follows:

1. BCV Calculation

If df_0 is finite, $B_{i,j}$ is sampled using a scaled inverse chi-square distribution:

$$B_{i,j} = \left(\phi + \frac{1}{\sqrt{\lambda'_{i,j}}} \right) \cdot \sqrt{\frac{df_0}{\chi^2_{df_0}}} \tag{2}$$

where $\chi^2_{df_o}$ is a chi-square-distributed variable with df_o degrees of freedom. In the limiting case where df_0 is infinite, the BCV simplifies to the global trend:

$$B_{i,j} = \phi + \frac{1}{\sqrt{\lambda'_{i,j}}} \tag{3}$$

This formulation ensures that peaks with lower base accessibility ($\lambda'_{i,j}$) exhibit higher relative variability, accurately reflecting the sparsity-driven noise in scATAC-seq data.

2. Gamma Sampling for Trended Means

To incorporate this variability, the final trended cell mean ($\lambda_{i,j}$) is sampled from a Gamma distribution. The distribution is re-parameterized using the base mean and the simulated BCV to ensure the expectation $E[\lambda_{i,j}] = \lambda'_{i,j}$:

$$\lambda_{i,j} \sim \text{Gamma}\left(\text{shape} = \frac{1}{B^2_{i,j}}, \text{scale} = \lambda'_{i,j} \cdot B^2_{i,j} \right) \tag{4}$$

By defining the shape as $1/B^2_{i,j}$ and the scale as $\lambda'_{i,j} \cdot B^2_{i,j}$, the model captures the biological dispersion of the reference dataset while maintaining the discrete, stochastic nature of single-cell chromatin accessibility.

Supplementary Note 3: Dispersion estimate

It is well established that scATAC-seq data exhibits a strong mean-variance trend, where regions with higher mean accessibility tend to show greater variability [1, 2]. This pattern arises due to inherent biological variability, technical noise in single-cell measurements and fundamental statistical property of count data. To accurately replicate this trend in simulated data, We enforce it in extended simPIC framework (Fig.2) by estimating biological coefficient of variation (BCV) from real data, with parameters ϕ and df_0 from a scaled inverse chi-squared distribution using the `estimateDisp` function from the `edgeR` package [3], where the scaling factor is a function of the peak mean. This ensures that simulated datasets capture realistic variability patterns observed in real single-cell ATAC-seq experiments. However, upon testing this estimation procedure on simulated datasets, we observed that the `edgeR` estimate of common dispersion was inflated, as shown in Fig. S1. This inflation has also been reported in the simulation of single-cell RNA-seq data using the `splatter` package [4]. To address this issue, we applied a linear correction to the edgeR-estimated dispersions using the formula $\hat{\phi} = -0.3 + 0.15\hat{\phi}_{\texttt{edgeR}}$.The coefficients of the linear dispersion correction were obtained from a joint regression of true dispersion values against edgeR's common dispersion estimates using simulations parameterised from six distinct real scATAC-seq datasets (Table 1). Despite differences in organism, platform, and sequencing technology, all datasets showed a highly similar inflation of edgeR-estimated dispersions. Applying a single set of coefficients consistently reduced this inflation and improved agreement between simulated and empirical mean–variance relationships. Owing to this consistency, we summarise the behaviour using a single representative example in Fig. S1 and adopt the resulting correction as an empirically calibrated default in simPIC. This correction helps to adjust for the overestimation of dispersion, leading to more accurate simulated data that better reflects the true variability observed in real datasets.

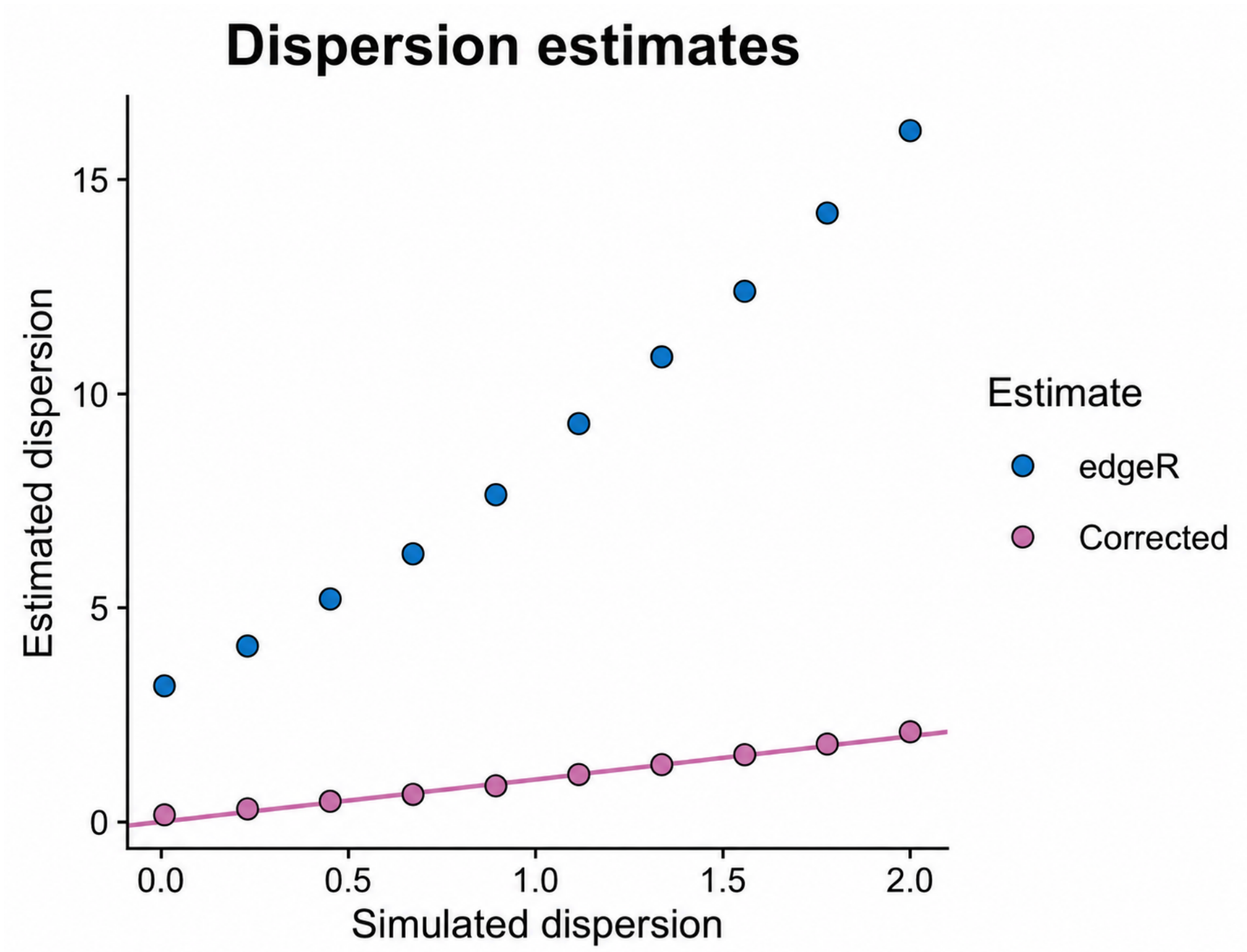

Fig. S1 Scatter plot of estimated dispersions against the true simulated values. Estimates of common dispersion obtained from edgeR (green) can be inflated for single-cell data. The SIMPIC simulation uses a linearly corrected value (orange) in its estimation procedure. The red line shows the true values estimates.

Supplementary Figures

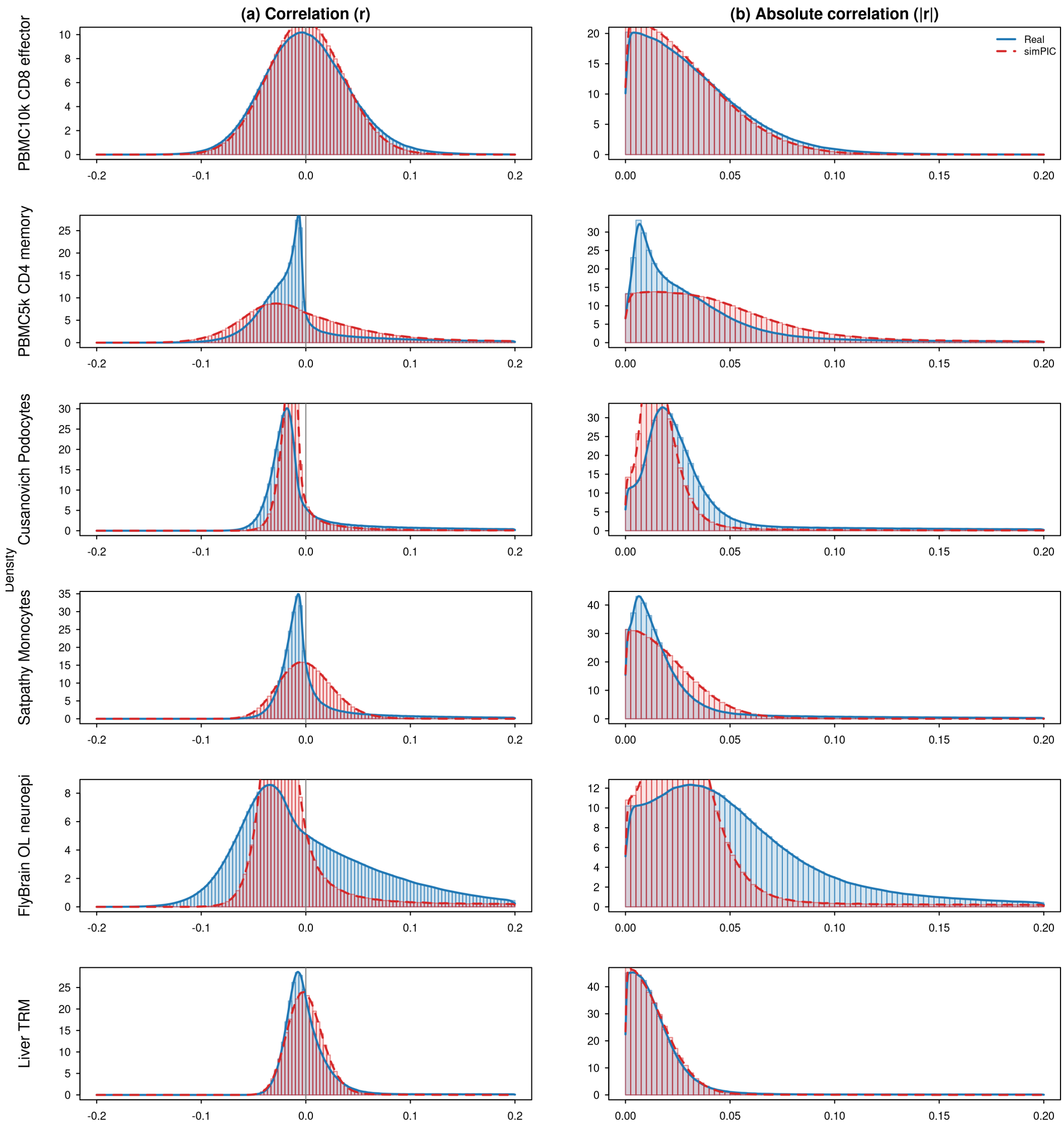

Fig. S2 Peak–peak correlation structure in real versus simPIC data. Pairwise peak–peak correlations across cells were computed on TF–IDF transformed accessibility using the top 2,000 variable peaks (filtered to peaks detected in ≥ 10 cells). (a) Signed correlations (r). (b) Absolute correlations ($|r|$). Blue solid: real; red dashed: simPIC.

Supplementary Figure S3

The below figures show all the 25 cell types evaluated in this study. The figures are divided into two pages for each cell type with cell type name indicated at top.

All figures present the same set of diagnostics in different cell types across the six datasets.

Top panels (a–i) show cell-level and peak-level summary distributions: (a) log library size, (b) log peak mean, (c) cell sparsity; (d–f) corresponding density distributions of log library size, log peak mean and cell sparsity; (g–i) density differences relative to real data.

The next page is of the same cell type and shows additional peak-level characteristics and mean-variance relationships: (a) peak sparsity, (b) log peak variance, (c) peak mean–variance relationship, (d) peak mean vs non-zero proportion, (e) peak mean vs peak sparsity; (f–g) density overlaps for peak sparsity and log peak variance; (h–i) corresponding density differences.

The numbers on top of violins indicate KS statistic.

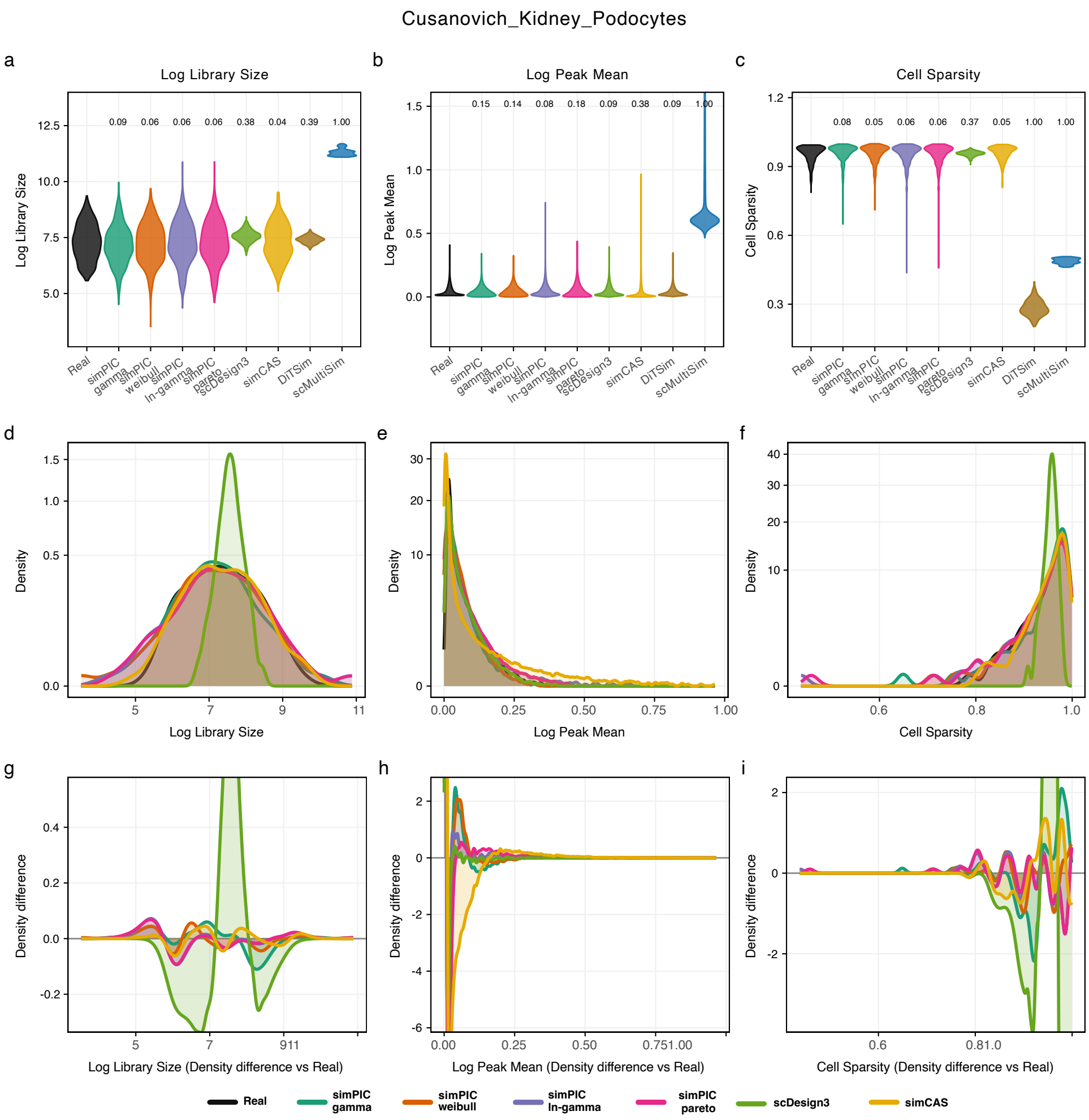
Cusanovich_Kidney_Podocytes
a
Log Library Size
Log Library Size
0.09 0.06 0.06 0.06 0.38 0.04 0.39 1.00
Real
simPIC gamma
simPIC weibull
simPIC ln-gamma
simPIC pareto
scDesign3
simCAS
DITSim
scMultiSim
b
Log Peak Mean
Log Peak Mean
0.15 0.14 0.08 0.18 0.09 0.38 0.09 1.00
c
Cell Sparsity
Cell Sparsity
0.08 0.05 0.06 0.06 0.37 0.05 1.00 1.00
d
Density
Log Library Size
e
Density
Log Peak Mean
f
Density
Cell Sparsity
g
Density difference
Log Library Size (Density difference vs Real)
h
Density difference
Log Peak Mean (Density difference vs Real)
i
Density difference
Cell Sparsity (Density difference vs Real)
Real
simPIC gamma
simPIC weibull
simPIC ln-gamma
simPIC pareto
scDesign3
simCAS

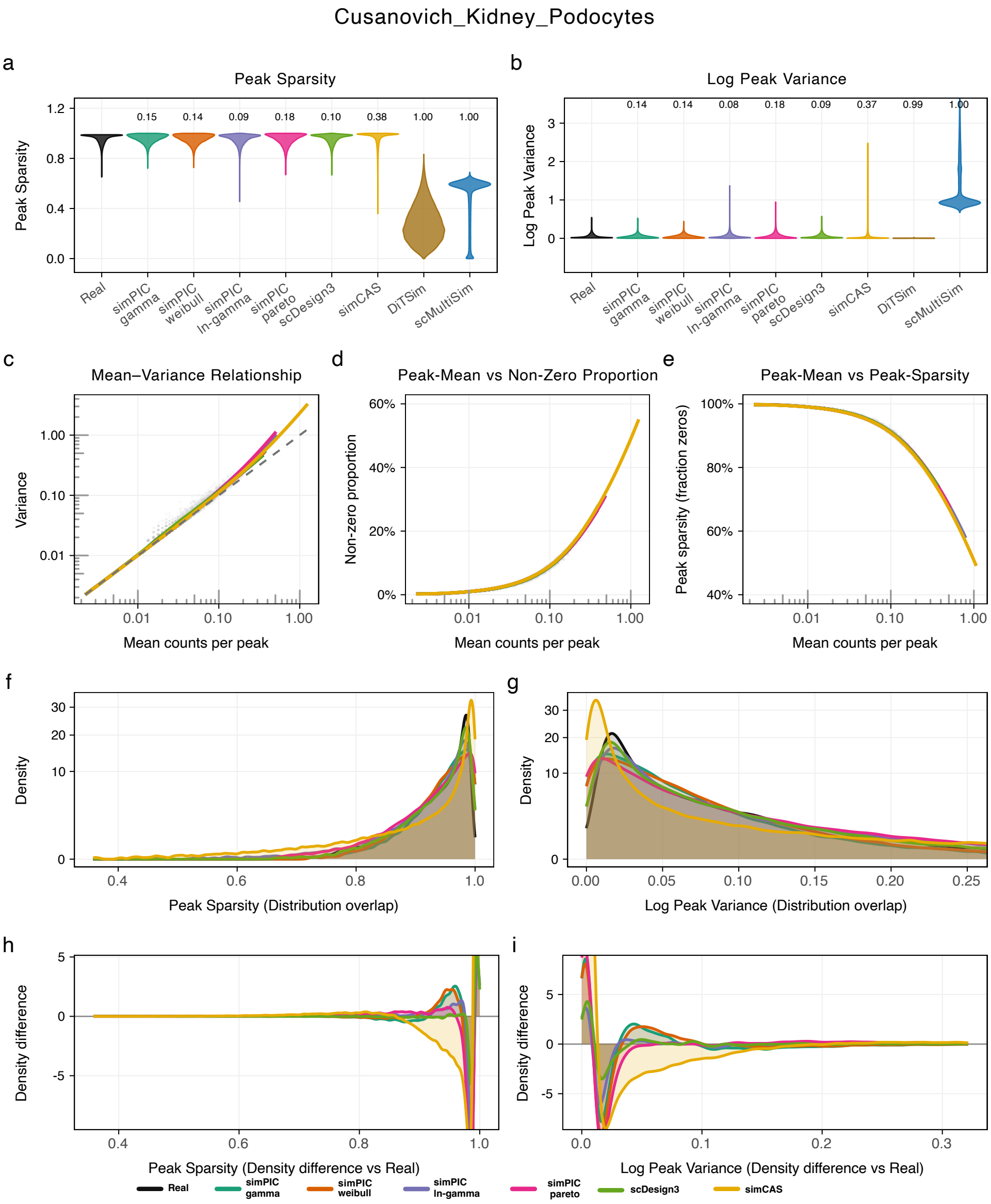
Cusanovich_Kidney_Podocytes
a
Peak Sparsity
b
Log Peak Variance
c
Mean–Variance Relationship
d
Peak-Mean vs Non-Zero Proportion
e
Peak-Mean vs Peak-Sparsity
f
g
h
i
Peak Sparsity
Log Peak Variance
Variance
Mean counts per peak
Non-zero proportion
Peak sparsity (fraction zeros)
Density
Peak Sparsity (Distribution overlap)
Log Peak Variance (Distribution overlap)
Density difference
Peak Sparsity (Density difference vs Real)
Log Peak Variance (Density difference vs Real)
Real
simPIC gamma
simPIC weibull
simPIC ln-gamma
simPIC pareto
scDesign3
simCAS
DiTSim
scMultiSim

Cusanovich_Cerebellum_Astrocytes

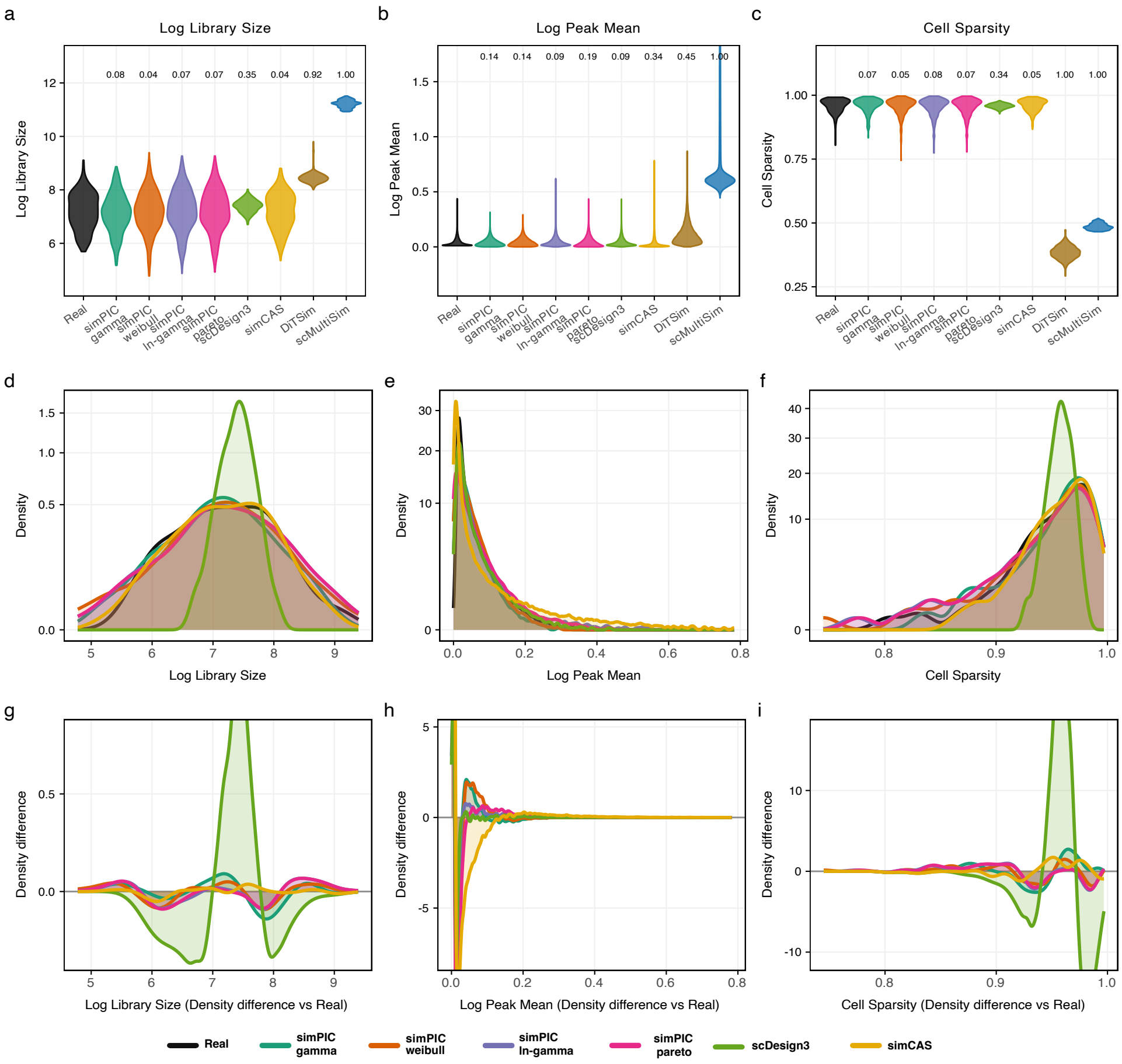

Cusanovich_Cerebellum_Astrocytes

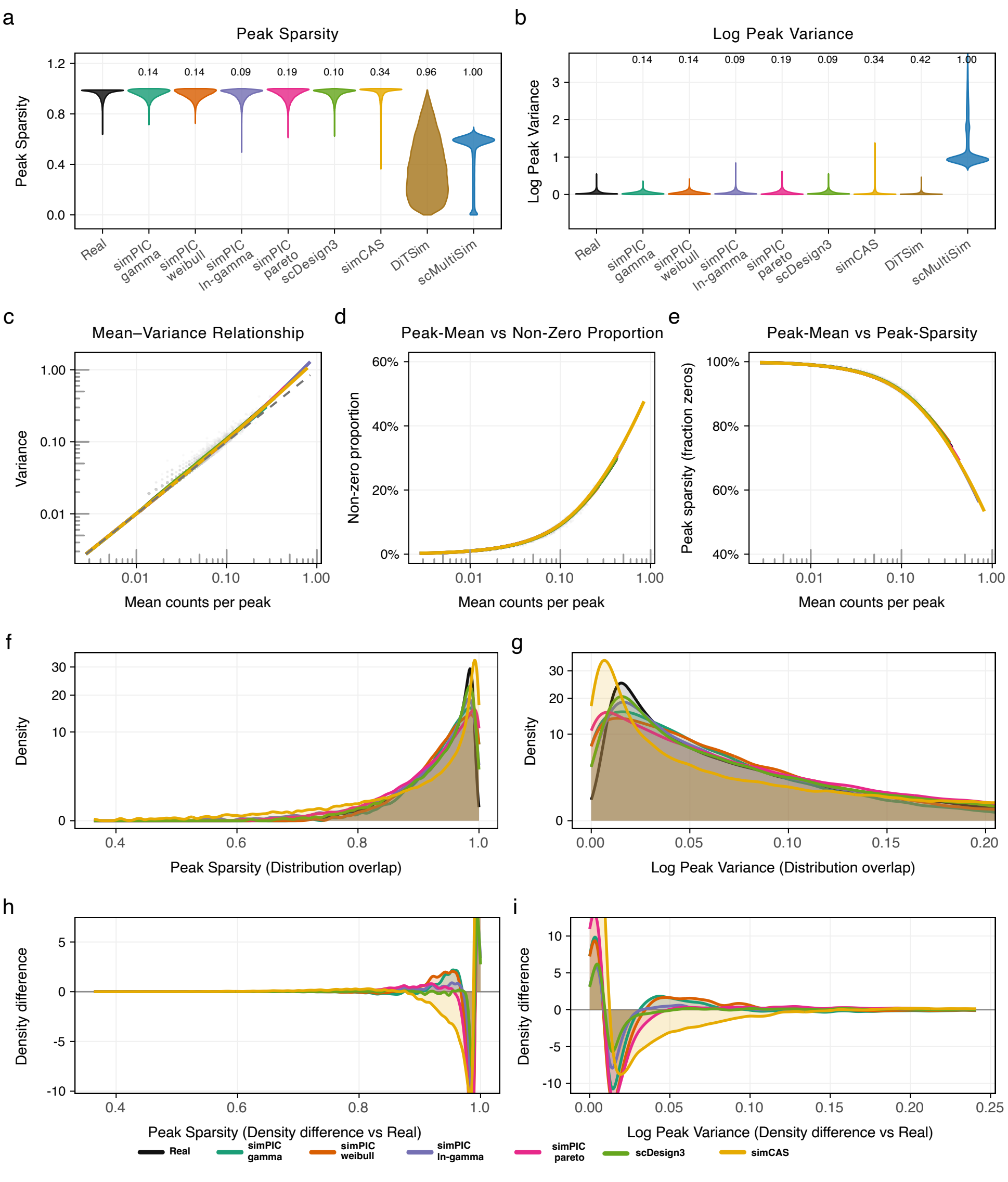

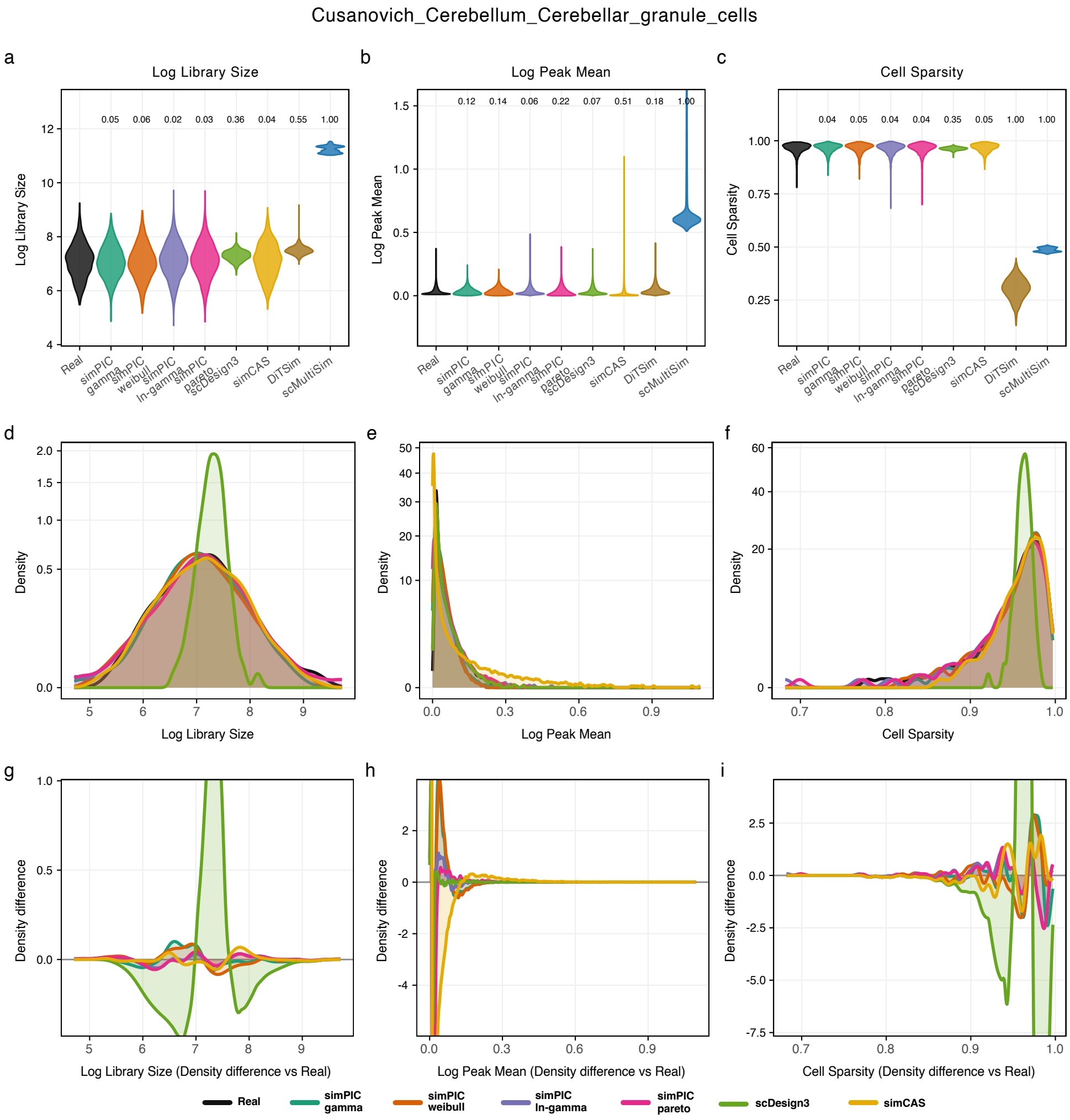

Cusanovich_Cerebellum_Cerebellar_granule_cells
a
Log Library Size
b
Log Peak Mean
c
Cell Sparsity
d
e
f
g
h
i
Log Library Size
Log Peak Mean
Cell Sparsity
Log Library Size (Density difference vs Real)
Log Peak Mean (Density difference vs Real)
Cell Sparsity (Density difference vs Real)
Density
Density difference
Real
simPIC gamma
simPIC weibull
simPIC ln-gamma
simPIC pareto
scDesign3
simCAS
DiTSim
scMultiSim

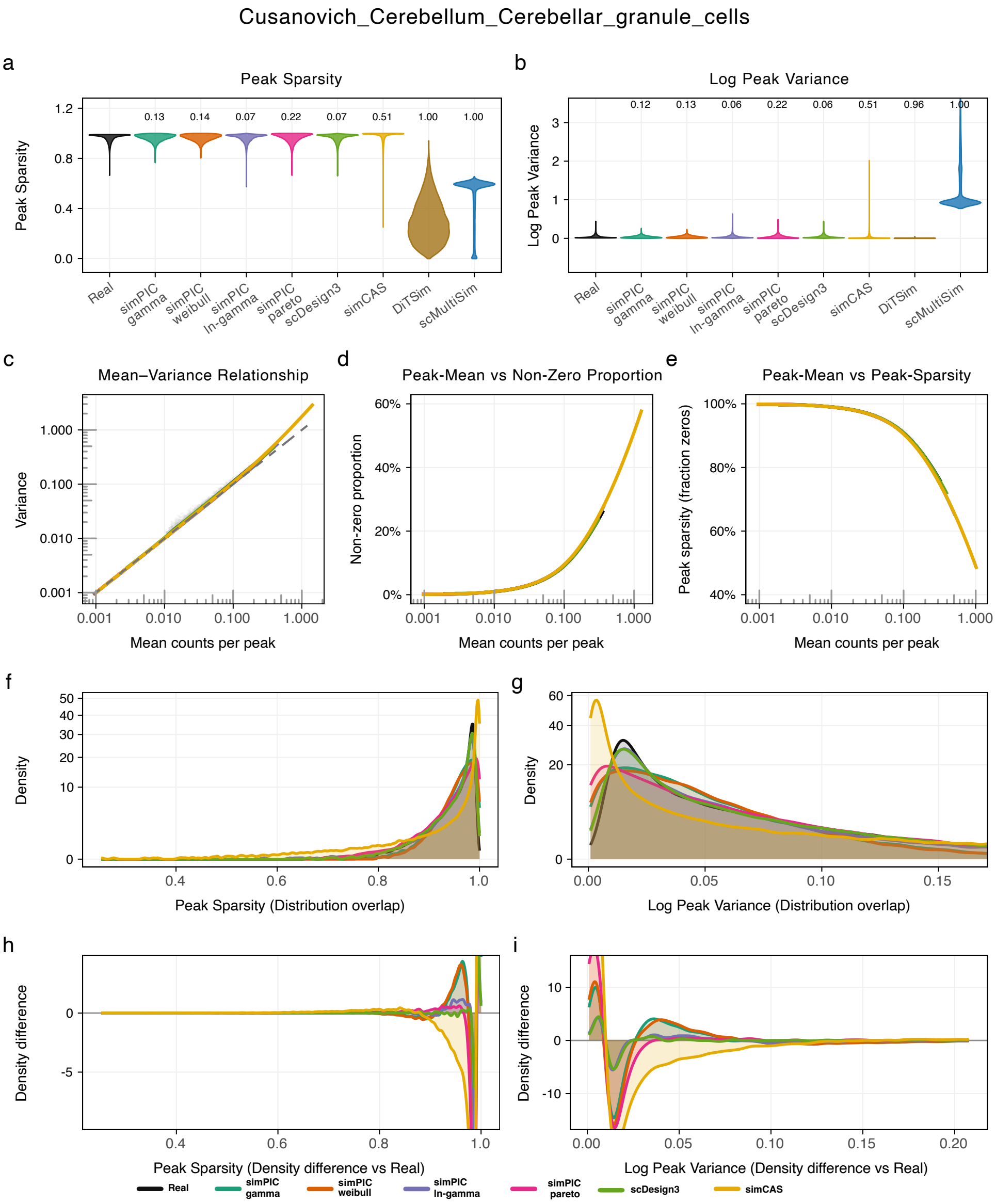
Cusanovich_Cerebellum_Cerebellar_granule_cells
a
Peak Sparsity
b
Log Peak Variance
c
Mean–Variance Relationship
d
Peak-Mean vs Non-Zero Proportion
e
Peak-Mean vs Peak-Sparsity
f
g
h
i
Peak Sparsity (Distribution overlap)
Log Peak Variance (Distribution overlap)
Peak Sparsity (Density difference vs Real)
Log Peak Variance (Density difference vs Real)
Real
simPIC gamma
simPIC weibull
simPIC ln-gamma
simPIC pareto
scDesign3
simCAS

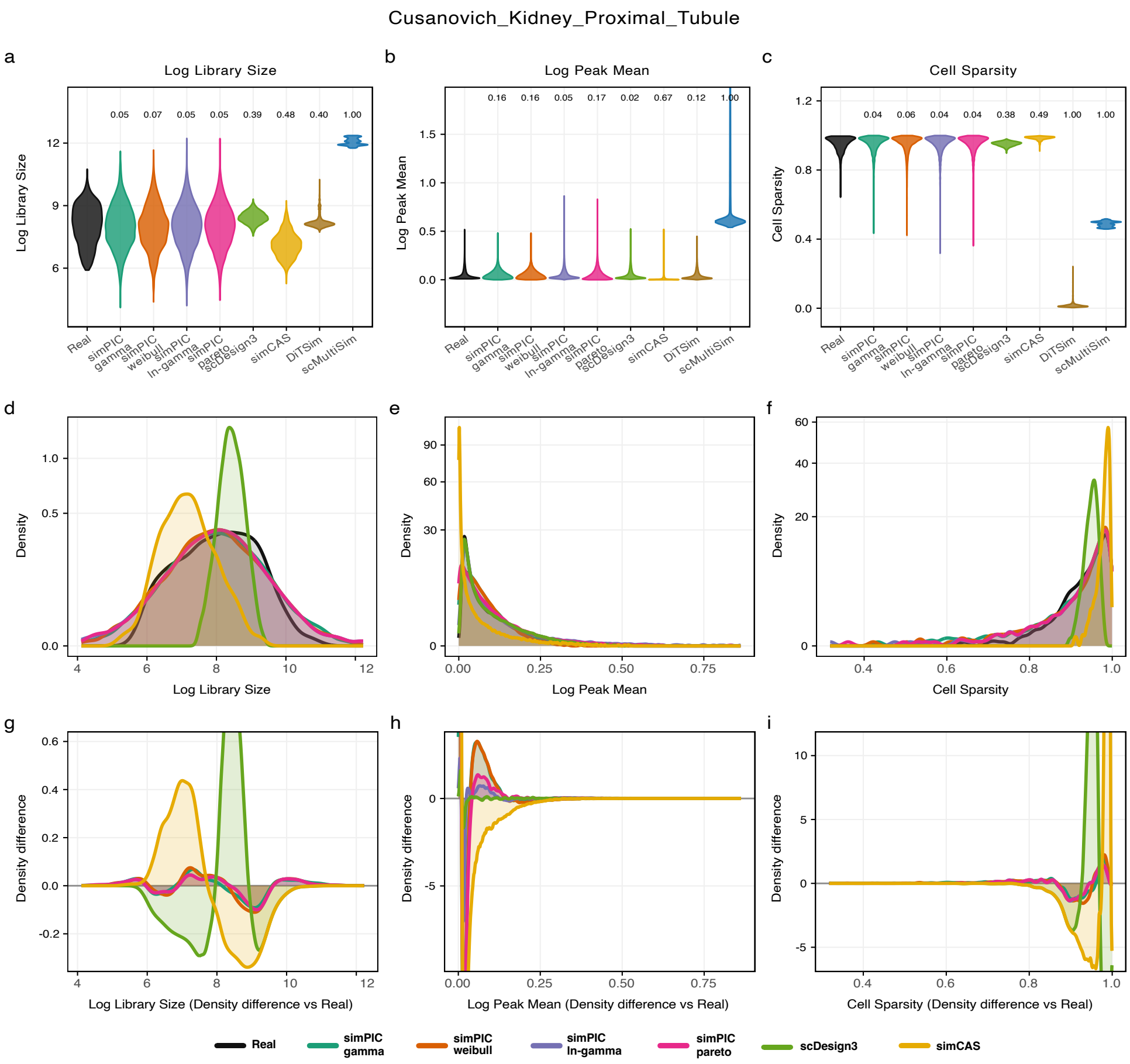
Cusanovich_Kidney_Proximal_Tubule
a
Log Library Size
b
Log Peak Mean
c
Cell Sparsity
d
e
f
g
h
i
Real
simPIC gamma
simPIC weibull
simPIC ln-gamma
simPIC pareto
scDesign3
simCAS
DiTSim
scMultiSim
Density
Log Library Size
Log Peak Mean
Cell Sparsity
Density difference
Log Library Size (Density difference vs Real)
Log Peak Mean (Density difference vs Real)
Cell Sparsity (Density difference vs Real)

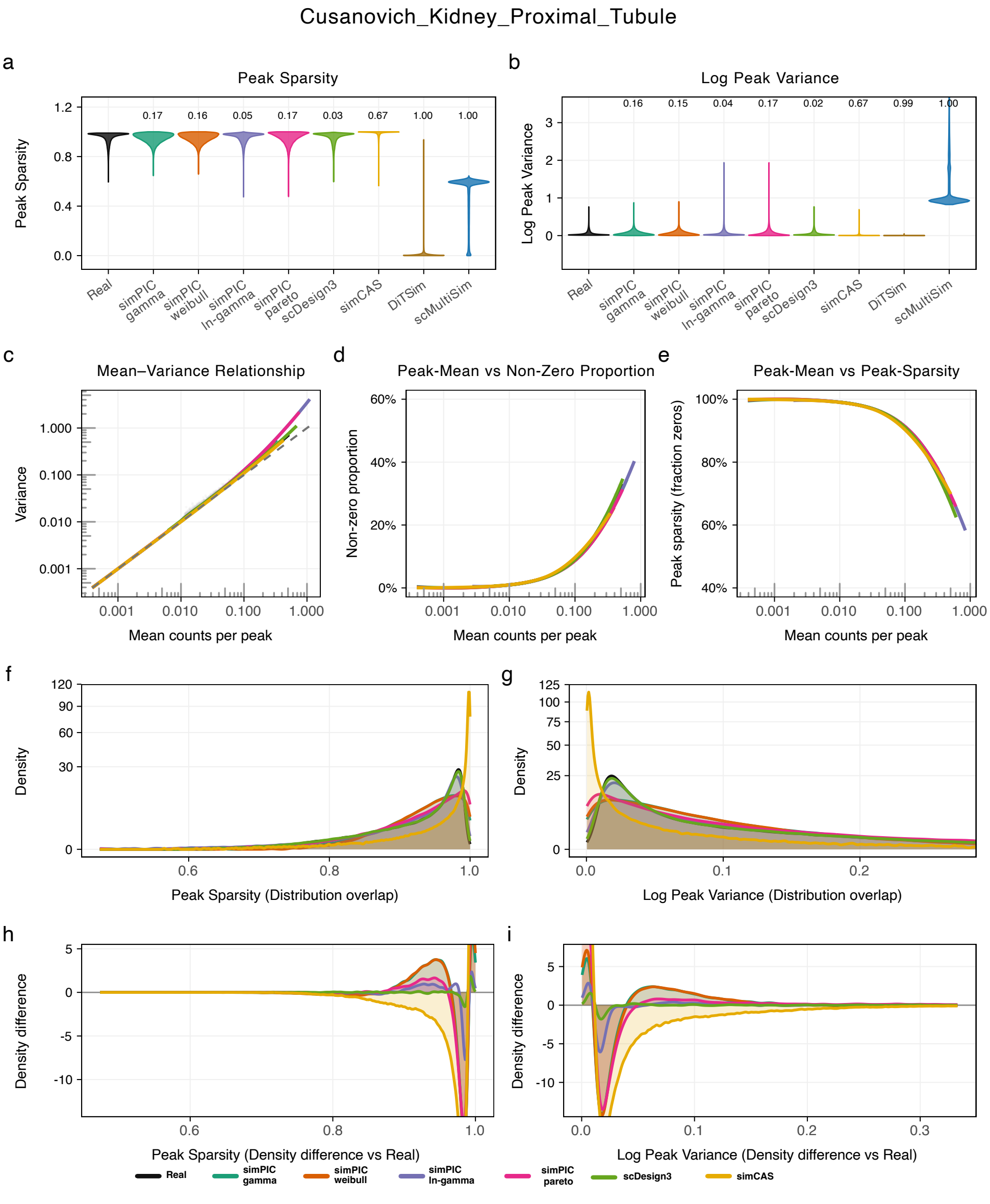

Cusanovich_Kidney_Proximal_Tubule
a
Peak Sparsity
b
Log Peak Variance
c
Mean–Variance Relationship
d
Peak-Mean vs Non-Zero Proportion
e
Peak-Mean vs Peak-Sparsity
f
g
h
i
Peak Sparsity
Log Peak Variance
Variance
Non-zero proportion
Peak sparsity (fraction zeros)
Mean counts per peak
Density
Peak Sparsity (Distribution overlap)
Log Peak Variance (Distribution overlap)
Density difference
Peak Sparsity (Density difference vs Real)
Log Peak Variance (Density difference vs Real)
Real
simPIC gamma
simPIC weibull
simPIC ln-gamma
simPIC pareto
scDesign3
simCAS
DiTSim
scMultiSim

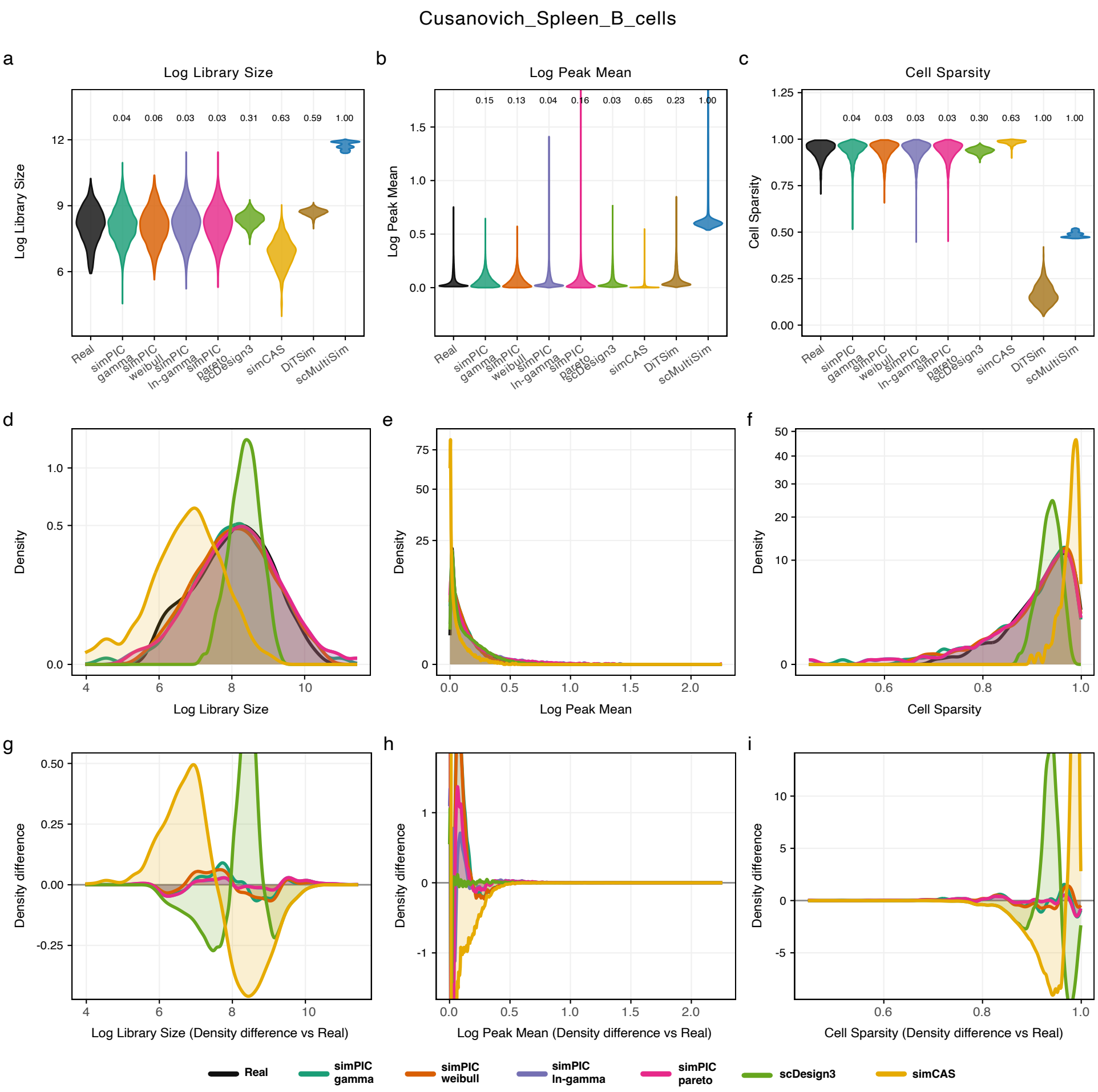
Cusanovich_Spleen_B_cells
a
Log Library Size
b
Log Peak Mean
c
Cell Sparsity
0.04 0.06 0.03 0.03 0.31 0.63 0.59 1.00
0.15 0.13 0.04 0.16 0.03 0.65 0.23 1.00
0.04 0.03 0.03 0.03 0.30 0.63 1.00 1.00
Log Library Size
Log Peak Mean
Cell Sparsity
Real
simPIC gamma
simPIC weibull
simPIC ln-gamma
simPIC pareto
scDesign3
simCAS
DiTSim
scMultiSim
d
e
f
Density
Log Library Size
Log Peak Mean
Cell Sparsity
g
h
i
Density difference
Log Library Size (Density difference vs Real)
Log Peak Mean (Density difference vs Real)
Cell Sparsity (Density difference vs Real)
Real
simPIC gamma
simPIC weibull
simPIC ln-gamma
simPIC pareto
scDesign3
simCAS

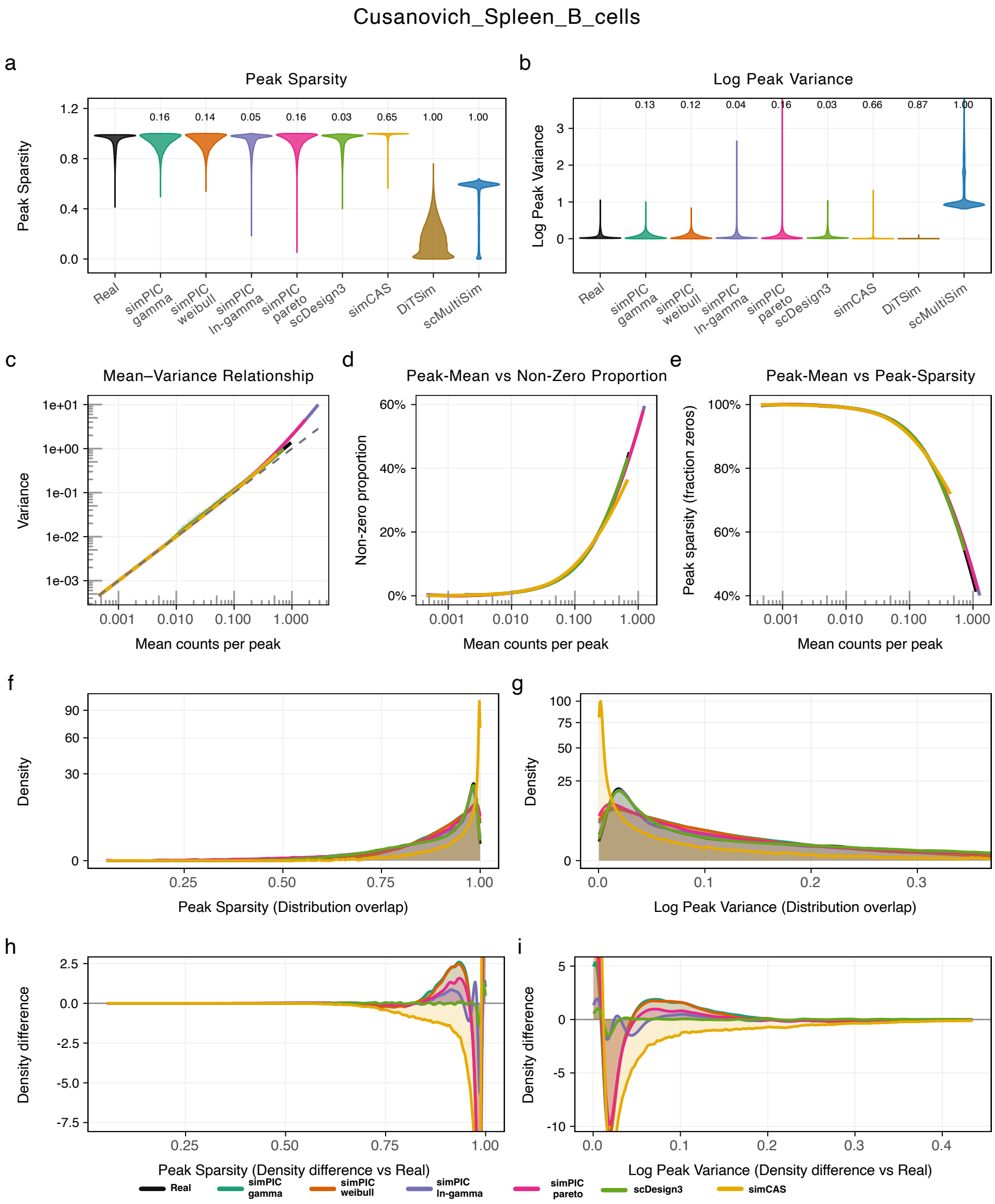
Cusanovich_Spleen_B_cells
a
Peak Sparsity
Peak Sparsity
0.16
0.14
0.05
0.16
0.03
0.65
1.00
1.00
Real
simPIC gamma
simPIC weibull
simPIC ln-gamma
simPIC pareto
scDesign3
simCAS
DiTSim
scMultiSim
b
Log Peak Variance
Log Peak Variance
0.13
0.12
0.04
0.16
0.03
0.66
0.87
1.00
c
Mean–Variance Relationship
Variance
Mean counts per peak
d
Peak-Mean vs Non-Zero Proportion
Non-zero proportion
Mean counts per peak
e
Peak-Mean vs Peak-Sparsity
Peak sparsity (fraction zeros)
Mean counts per peak
f
Density
Peak Sparsity (Distribution overlap)
g
Density
Log Peak Variance (Distribution overlap)
h
Density difference
Peak Sparsity (Density difference vs Real)
i
Density difference
Log Peak Variance (Density difference vs Real)
Real
simPIC gamma
simPIC weibull
simPIC ln-gamma
simPIC pareto
scDesign3
simCAS

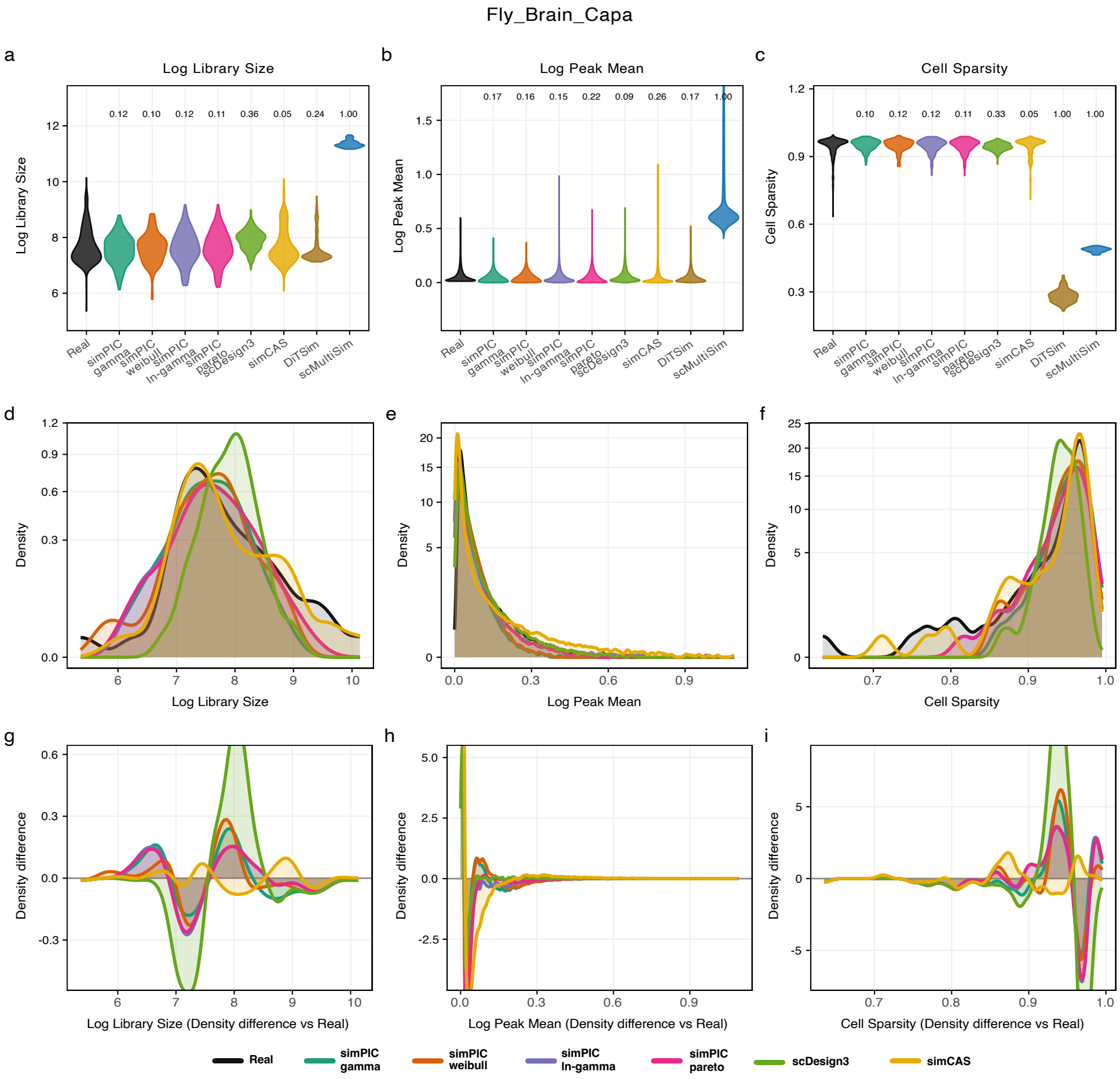

Fly_Brain_Capa
a
Log Library Size
b
Log Peak Mean
c
Cell Sparsity
Log Library Size
Log Peak Mean
Cell Sparsity
0.12 0.10 0.12 0.11 0.36 0.05 0.24 1.00
0.17 0.16 0.15 0.22 0.09 0.26 0.17 1.00
0.10 0.12 0.12 0.11 0.33 0.05 1.00 1.00
Real
simPIC gamma
simPIC weibull
simPIC ln-gamma
simPIC pareto
scDesign3
simCAS
DiTSim
scMultiSim
d
e
f
Density
Log Library Size
Log Peak Mean
Cell Sparsity
g
h
i
Density difference
Log Library Size (Density difference vs Real)
Log Peak Mean (Density difference vs Real)
Cell Sparsity (Density difference vs Real)
Real
simPIC gamma
simPIC weibull
simPIC ln-gamma
simPIC pareto
scDesign3
simCAS

Fly_Brain_Capa

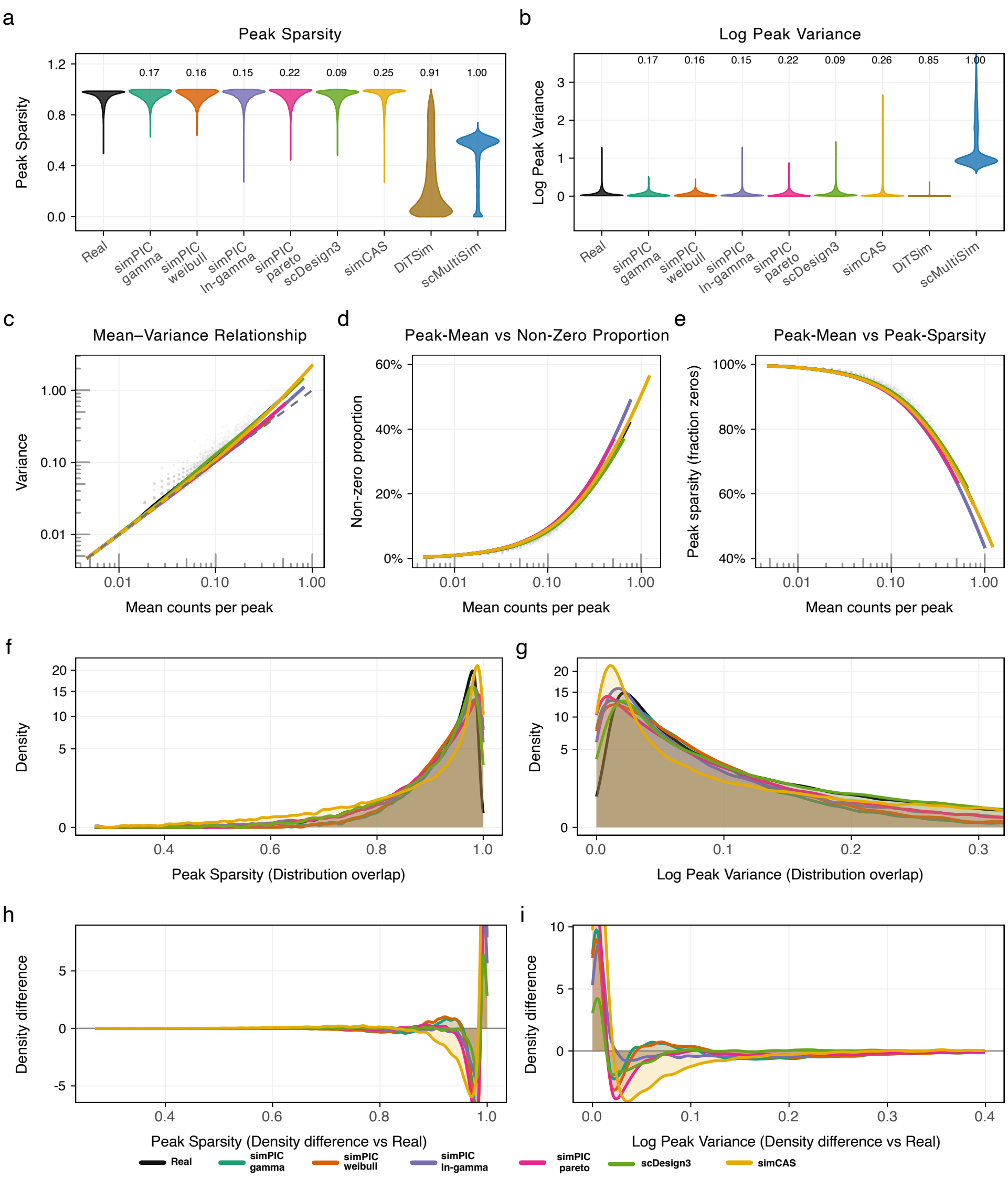

Fly_Brain_CB_Type_I_NB

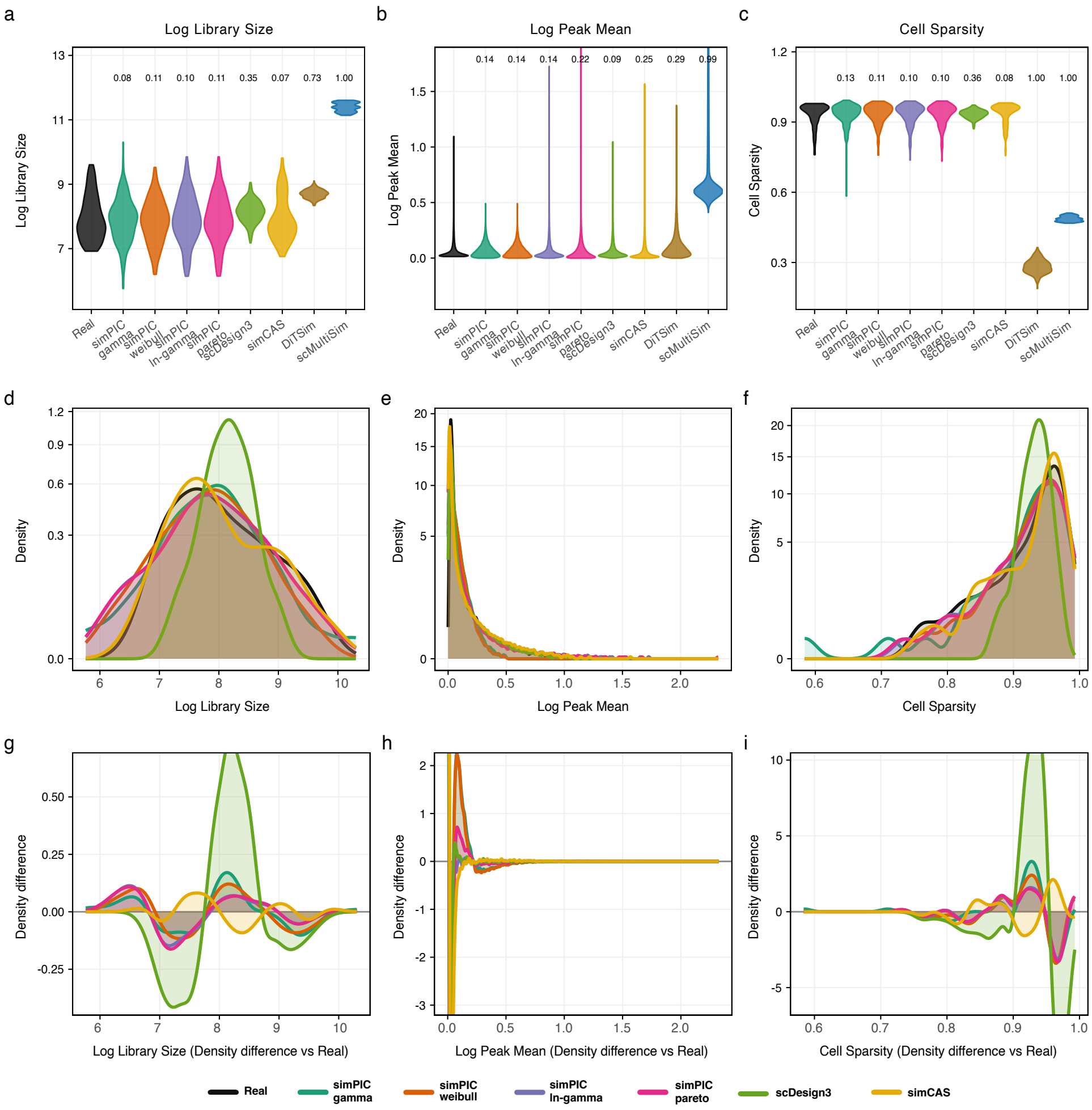

Fly_Brain_CB_Type_I_NB

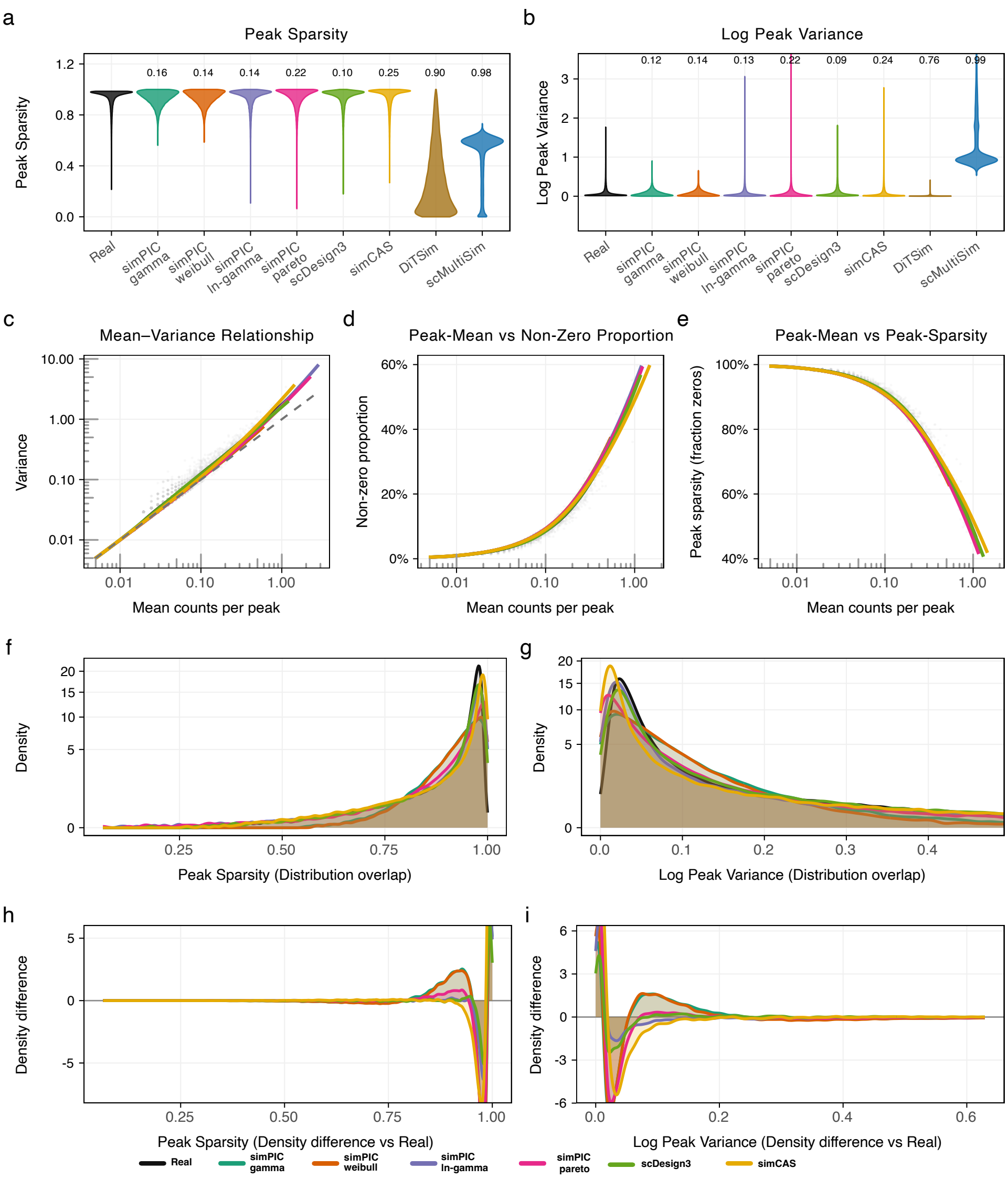

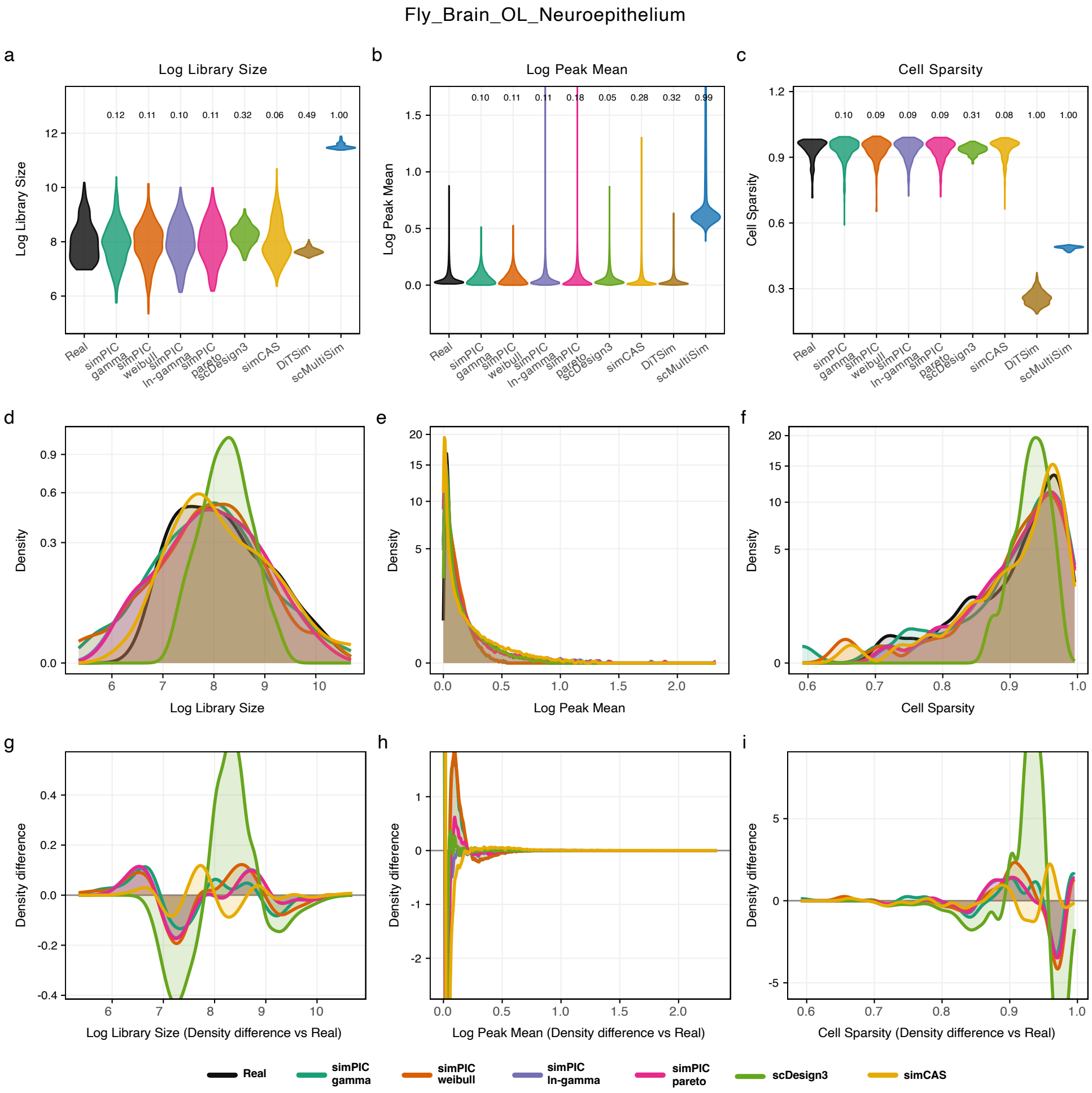
Fly_Brain_OL_Neuroepithelium
a
Log Library Size
b
Log Peak Mean
c
Cell Sparsity
d
e
f
g
h
i
Real
simPIC gamma
simPIC weibull
simPIC ln-gamma
simPIC pareto
scDesign3
simCAS
DiTSim
scMultiSim
Log Library Size
Log Peak Mean
Cell Sparsity
Density
Density difference
Log Library Size (Density difference vs Real)
Log Peak Mean (Density difference vs Real)
Cell Sparsity (Density difference vs Real)

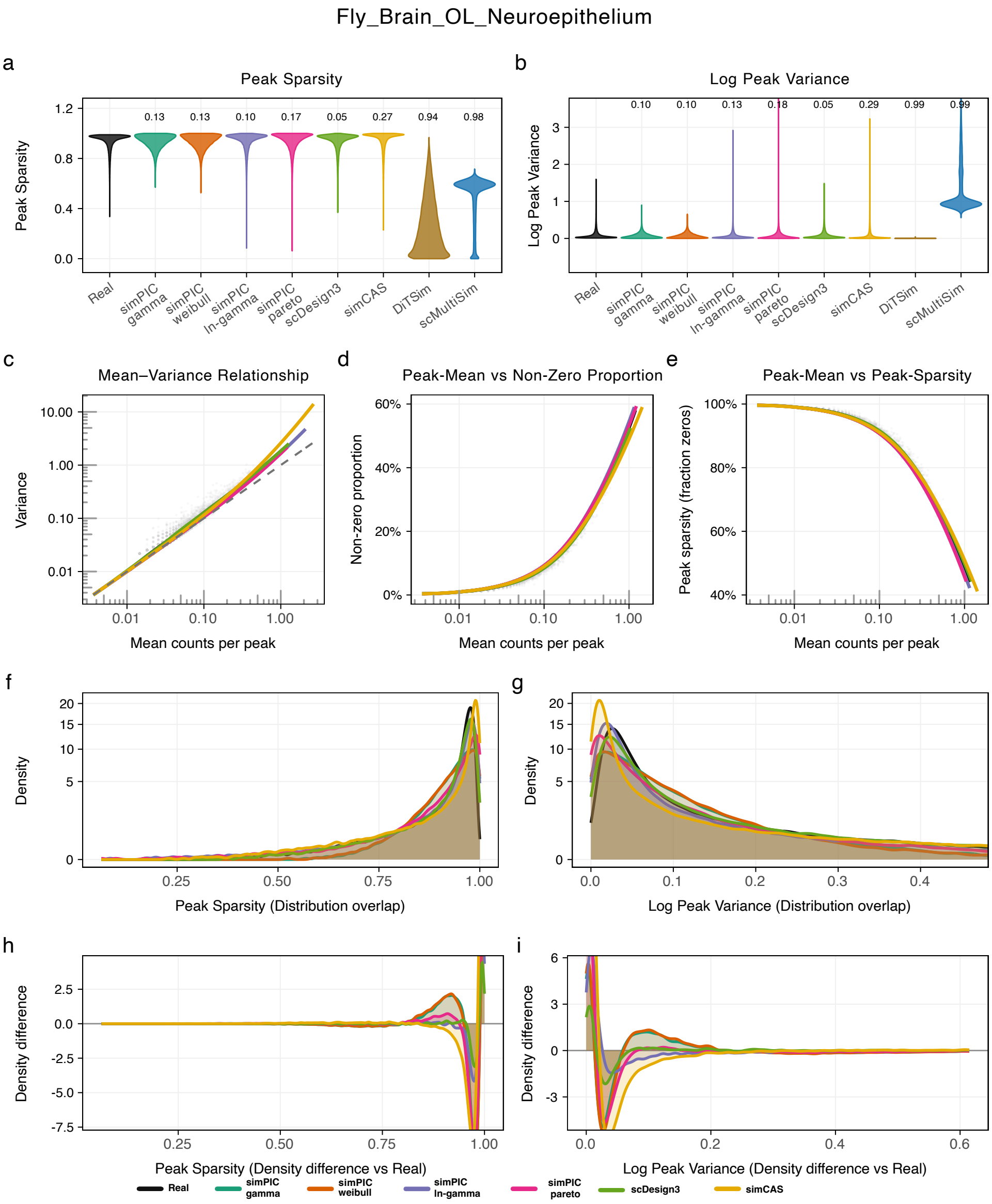

Fly_Brain_OL_Neuroepithelium
a
Peak Sparsity
Peak Sparsity
0.13
0.13
0.10
0.17
0.05
0.27
0.94
0.98
Real
simPIC gamma
simPIC weibull
simPIC ln-gamma
simPIC pareto
scDesign3
simCAS
DiTSim
scMultiSim
b
Log Peak Variance
Log Peak Variance
0.10
0.10
0.13
0.18
0.05
0.29
0.99
0.99
c
Mean–Variance Relationship
Variance
Mean counts per peak
d
Peak-Mean vs Non-Zero Proportion
Non-zero proportion
Mean counts per peak
e
Peak-Mean vs Peak-Sparsity
Peak sparsity (fraction zeros)
Mean counts per peak
f
Density
Peak Sparsity (Distribution overlap)
g
Density
Log Peak Variance (Distribution overlap)
h
Density difference
Peak Sparsity (Density difference vs Real)
i
Density difference
Log Peak Variance (Density difference vs Real)
Real
simPIC gamma
simPIC weibull
simPIC ln-gamma
simPIC pareto
scDesign3
simCAS

Fly_Brain_OL_Type_I_NB

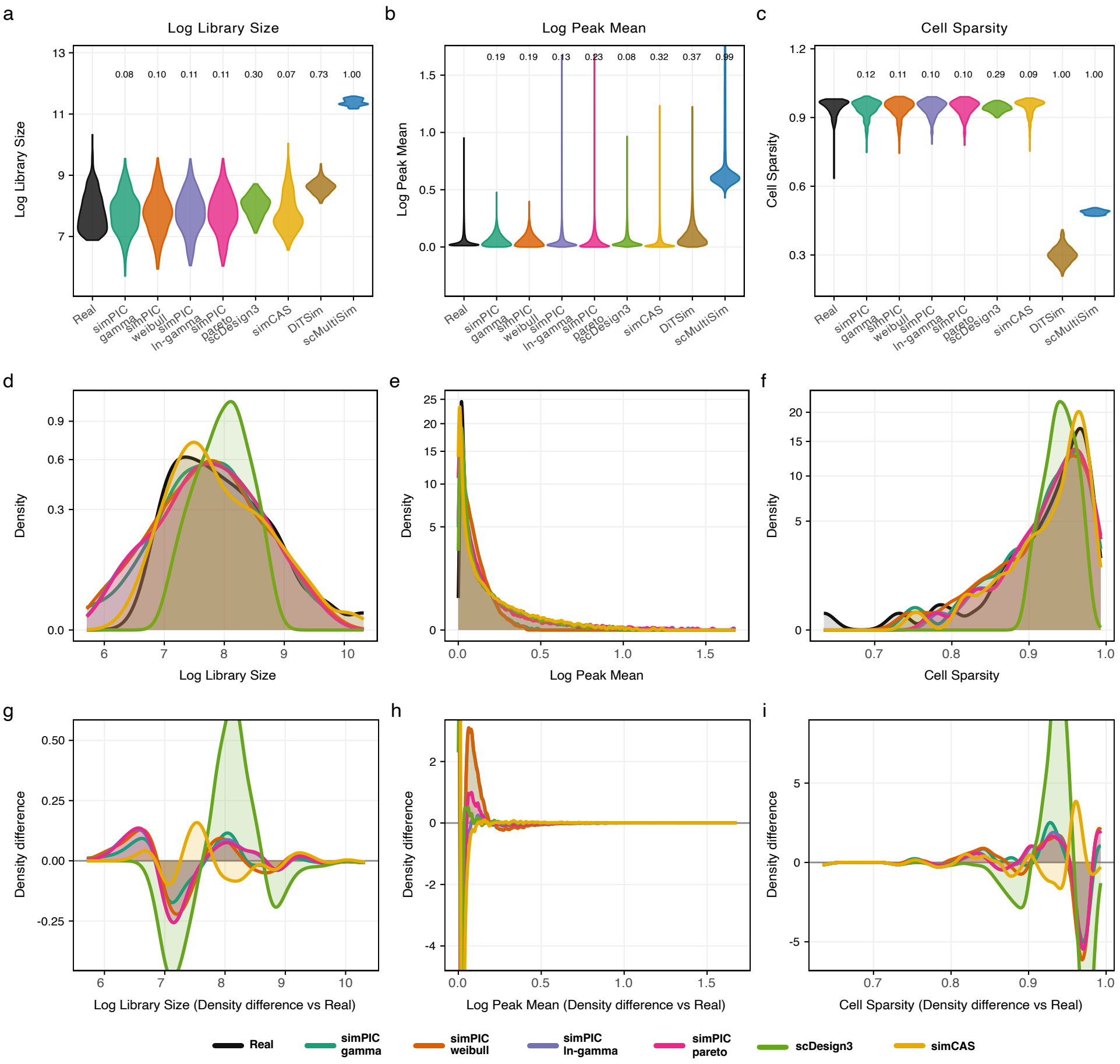

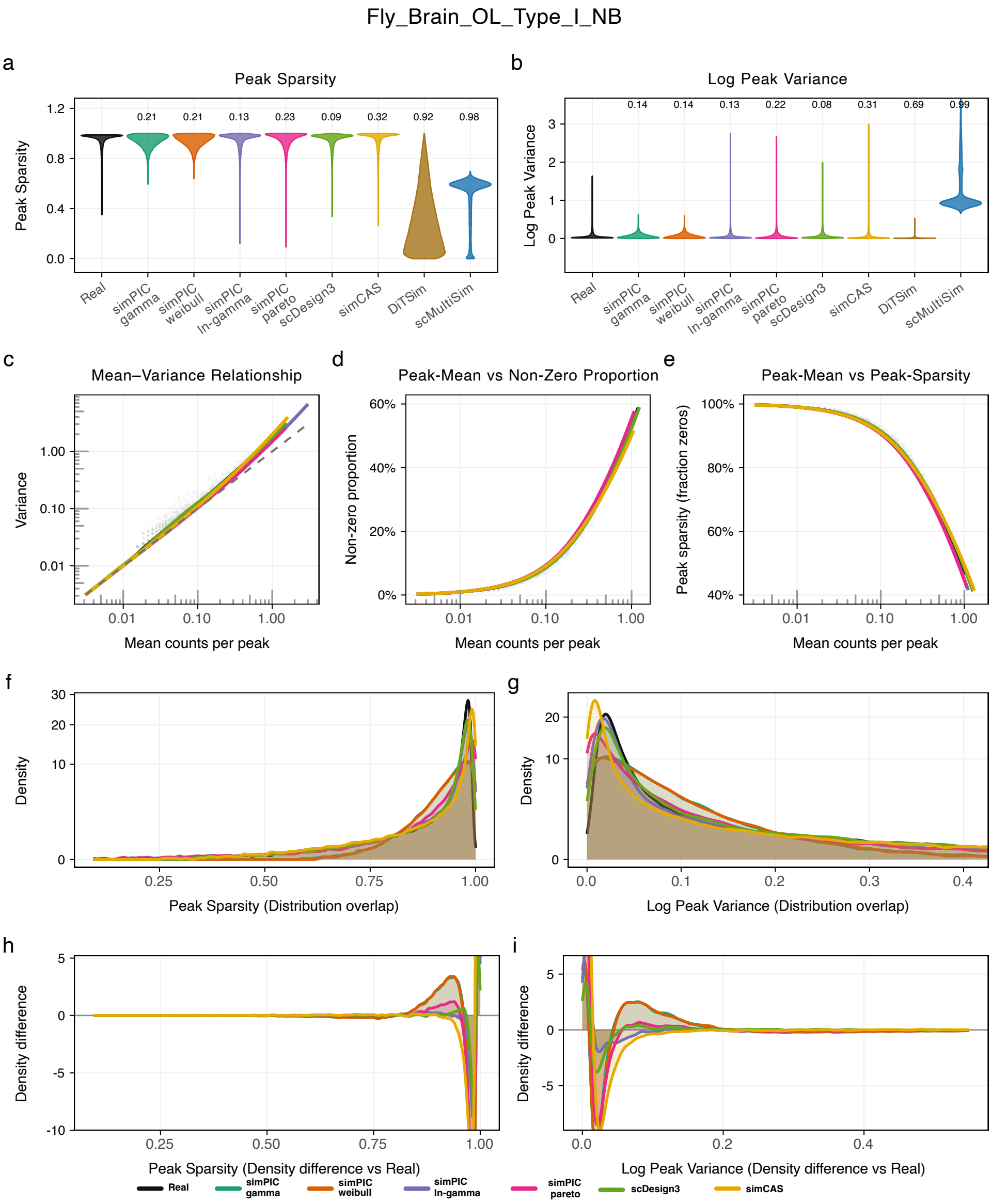
Fly_Brain_OL_Type_I_NB
a
Peak Sparsity
Peak Sparsity
0.21
0.21
0.13
0.23
0.09
0.32
0.92
0.98
Real
simPIC gamma
simPIC weibull
simPIC ln-gamma
simPIC pareto
scDesign3
simCAS
DiTSim
scMultiSim
b
Log Peak Variance
Log Peak Variance
0.14
0.14
0.13
0.22
0.08
0.31
0.69
0.99
c
Mean–Variance Relationship
Variance
Mean counts per peak
d
Peak-Mean vs Non-Zero Proportion
Non-zero proportion
Mean counts per peak
e
Peak-Mean vs Peak-Sparsity
Peak sparsity (fraction zeros)
Mean counts per peak
f
Density
Peak Sparsity (Distribution overlap)
g
Density
Log Peak Variance (Distribution overlap)
h
Density difference
Peak Sparsity (Density difference vs Real)
i
Density difference
Log Peak Variance (Density difference vs Real)
Real
simPIC gamma
simPIC weibull
simPIC ln-gamma
simPIC pareto
scDesign3
simCAS

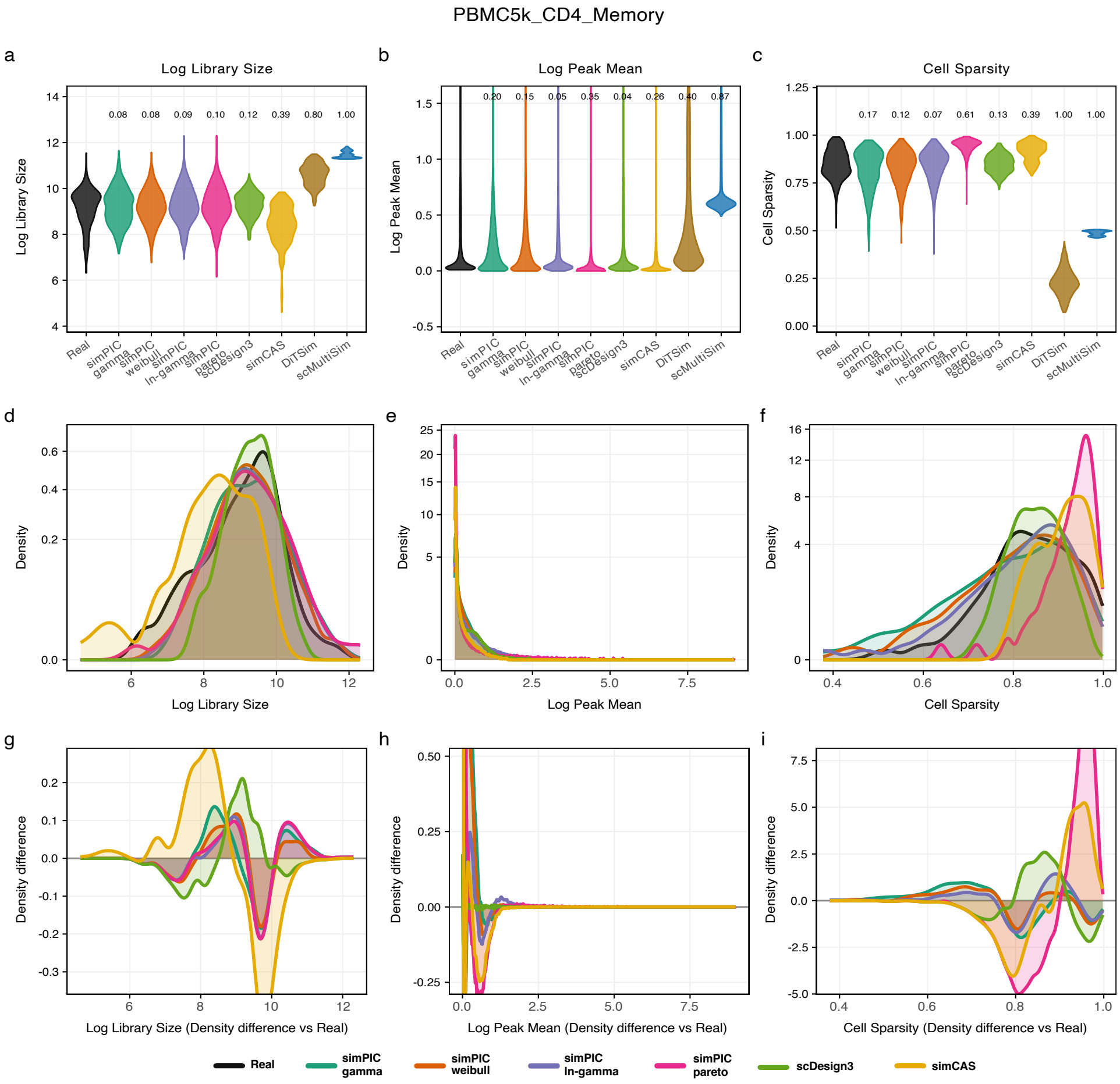
PBMC5k_CD4_Memory
a
Log Library Size
b
Log Peak Mean
c
Cell Sparsity
d
e
f
g
h
i
Log Library Size
Log Peak Mean
Cell Sparsity
Density
Density difference
Log Library Size (Density difference vs Real)
Log Peak Mean (Density difference vs Real)
Cell Sparsity (Density difference vs Real)
Real
simPIC gamma
simPIC weibull
simPIC ln-gamma
simPIC pareto
scDesign3
simCAS
DiTSim
scMultiSim

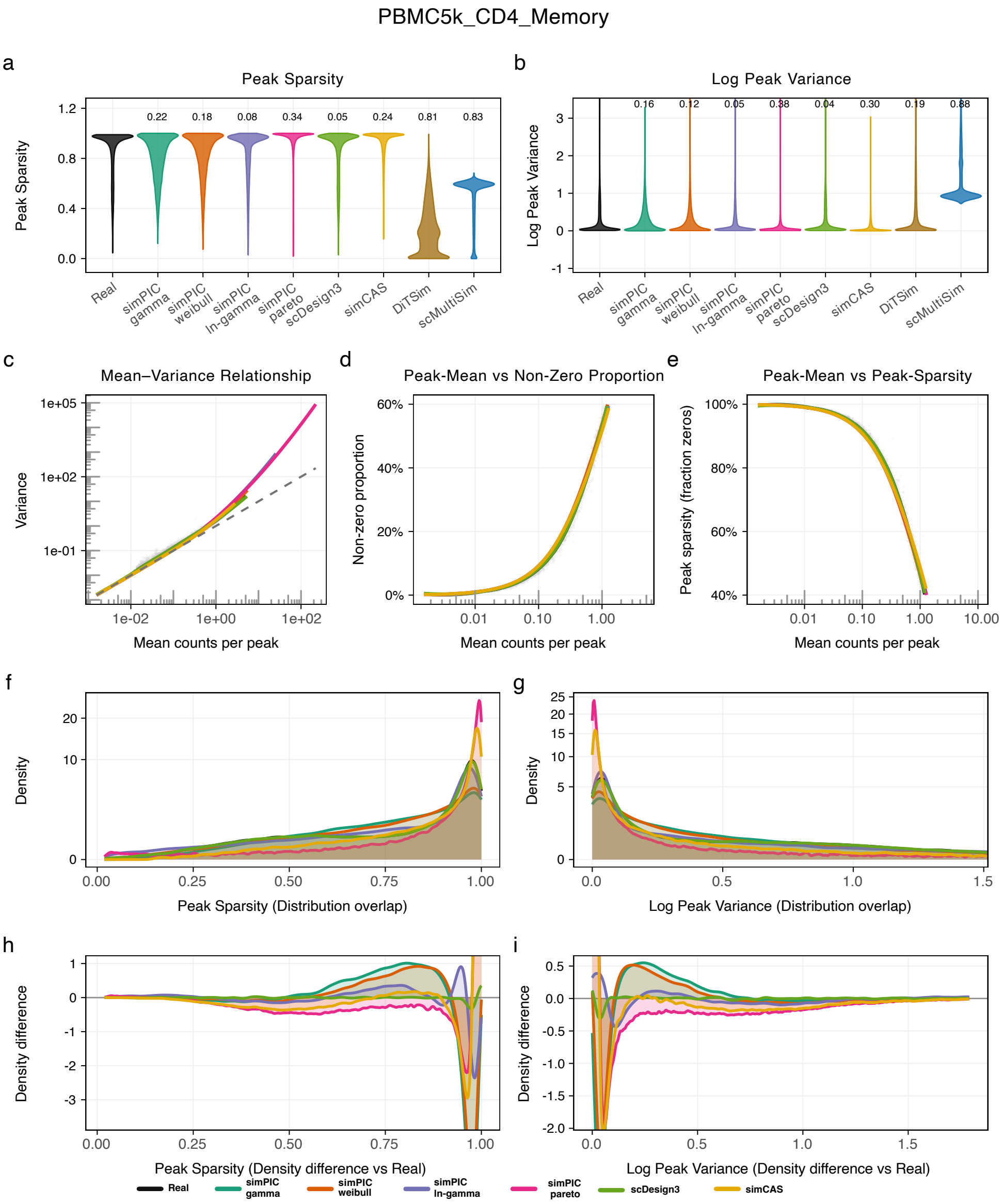
PBMC5k_CD4_Memory
a
Peak Sparsity
Peak Sparsity
0.22
0.18
0.08
0.34
0.05
0.24
0.81
0.83
Real
simPIC gamma
simPIC weibull
simPIC ln-gamma
simPIC pareto
scDesign3
simCAS
DiTSim
scMultiSim
b
Log Peak Variance
Log Peak Variance
0.16
0.12
0.05
0.38
0.04
0.30
0.19
0.88
c
Mean–Variance Relationship
Variance
Mean counts per peak
d
Peak-Mean vs Non-Zero Proportion
Non-zero proportion
Mean counts per peak
e
Peak-Mean vs Peak-Sparsity
Peak sparsity (fraction zeros)
Mean counts per peak
f
Density
Peak Sparsity (Distribution overlap)
g
Density
Log Peak Variance (Distribution overlap)
h
Density difference
Peak Sparsity (Density difference vs Real)
i
Density difference
Log Peak Variance (Density difference vs Real)
Real
simPIC gamma
simPIC weibull
simPIC ln-gamma
simPIC pareto
scDesign3
simCAS

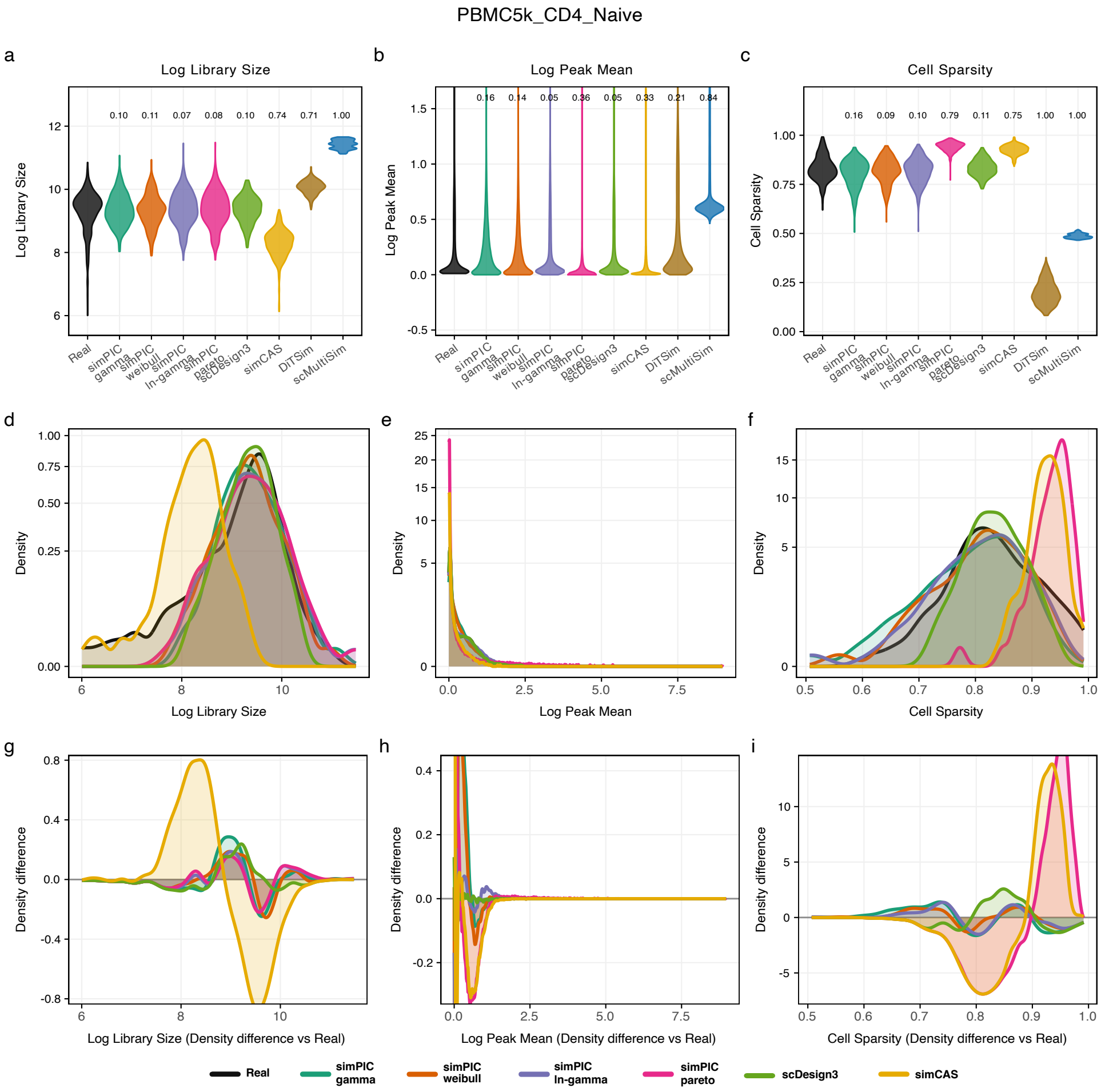
PBMC5k_CD4_Naive
a
Log Library Size
b
Log Peak Mean
c
Cell Sparsity
Log Library Size
Log Peak Mean
Cell Sparsity
Real
simPIC gamma
simPIC weibull
simPIC ln-gamma
simPIC pareto
scDesign3
simCAS
DiTSim
scMultiSim
d
e
f
Density
Log Library Size
Log Peak Mean
Cell Sparsity
g
h
i
Density difference
Log Library Size (Density difference vs Real)
Log Peak Mean (Density difference vs Real)
Cell Sparsity (Density difference vs Real)
Real
simPIC gamma
simPIC weibull
simPIC ln-gamma
simPIC pareto
scDesign3
simCAS

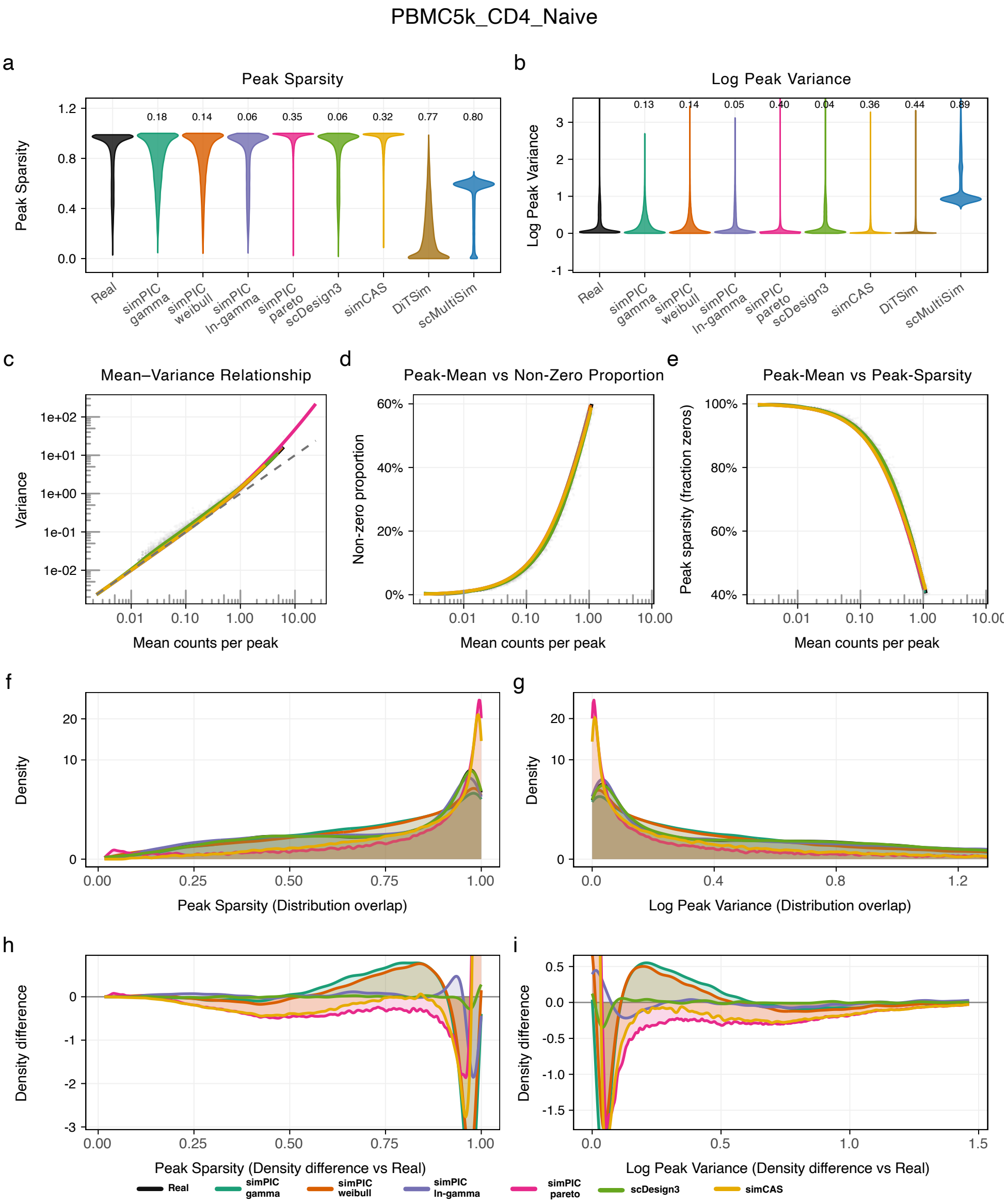

PBMC5k_CD4_Naive
a
Peak Sparsity
Peak Sparsity
0.18 0.14 0.06 0.35 0.06 0.32 0.77 0.80
Real
simPIC gamma
simPIC weibull
simPIC ln-gamma
simPIC pareto
scDesign3
simCAS
DiTSim
scMultiSim
b
Log Peak Variance
Log Peak Variance
0.13 0.14 0.05 0.40 0.04 0.36 0.44 0.89
c
Mean–Variance Relationship
Variance
Mean counts per peak
d
Peak-Mean vs Non-Zero Proportion
Non-zero proportion
Mean counts per peak
e
Peak-Mean vs Peak-Sparsity
Peak sparsity (fraction zeros)
Mean counts per peak
f
Density
Peak Sparsity (Distribution overlap)
g
Density
Log Peak Variance (Distribution overlap)
h
Density difference
Peak Sparsity (Density difference vs Real)
i
Density difference
Log Peak Variance (Density difference vs Real)
Real
simPIC gamma
simPIC weibull
simPIC ln-gamma
simPIC pareto
scDesign3
simCAS

PBMC5k_CD8_Effector

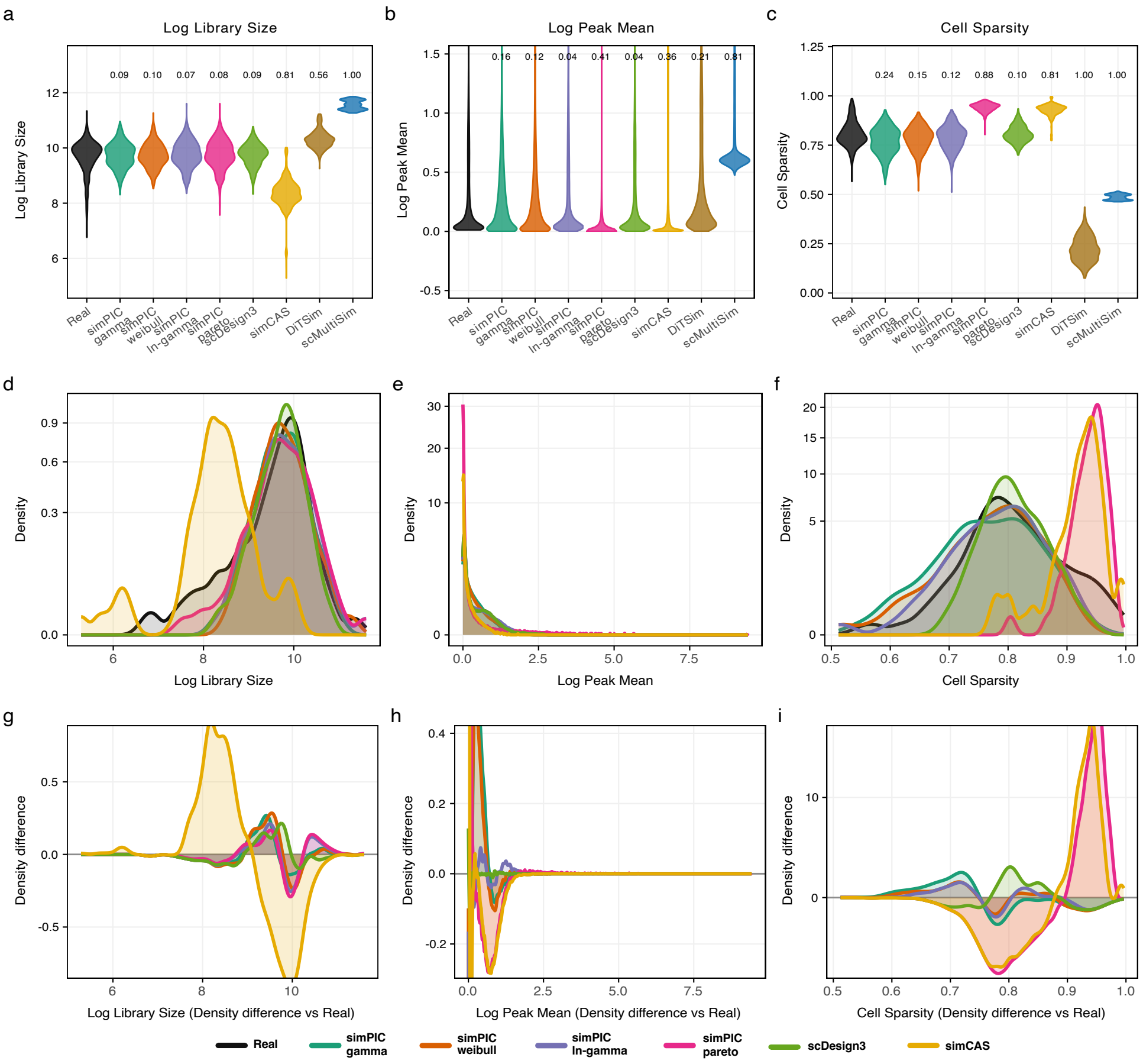

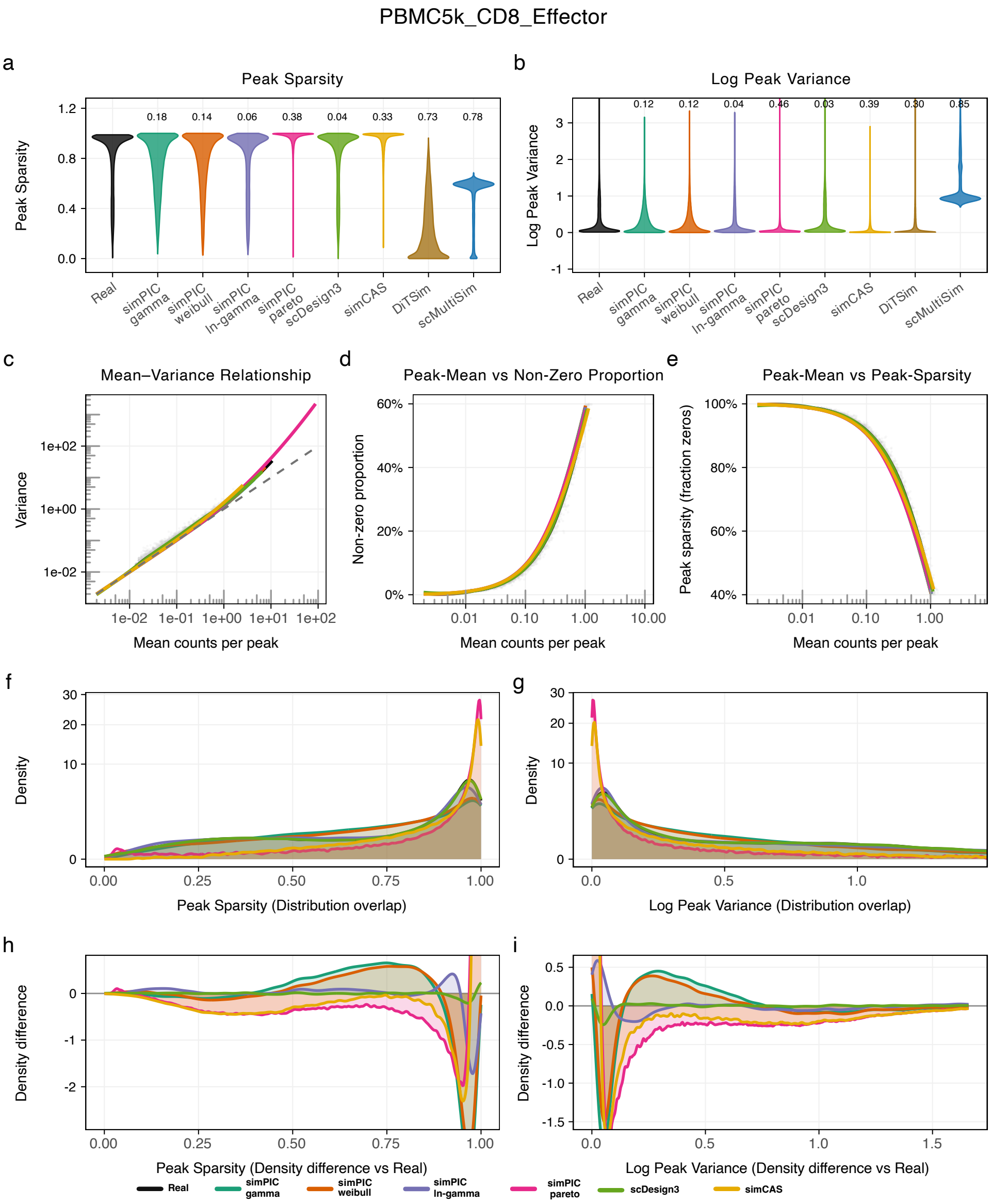
PBMC5k_CD8_Effector
a
Peak Sparsity
Peak Sparsity
b
Log Peak Variance
Log Peak Variance
Real
simPIC gamma
simPIC weibull
simPIC ln-gamma
simPIC pareto
scDesign3
simCAS
DiTSim
scMultiSim
c
Mean–Variance Relationship
Variance
Mean counts per peak
d
Peak-Mean vs Non-Zero Proportion
Non-zero proportion
Mean counts per peak
e
Peak-Mean vs Peak-Sparsity
Peak sparsity (fraction zeros)
Mean counts per peak
f
Density
Peak Sparsity (Distribution overlap)
g
Density
Log Peak Variance (Distribution overlap)
h
Density difference
Peak Sparsity (Density difference vs Real)
i
Density difference
Log Peak Variance (Density difference vs Real)

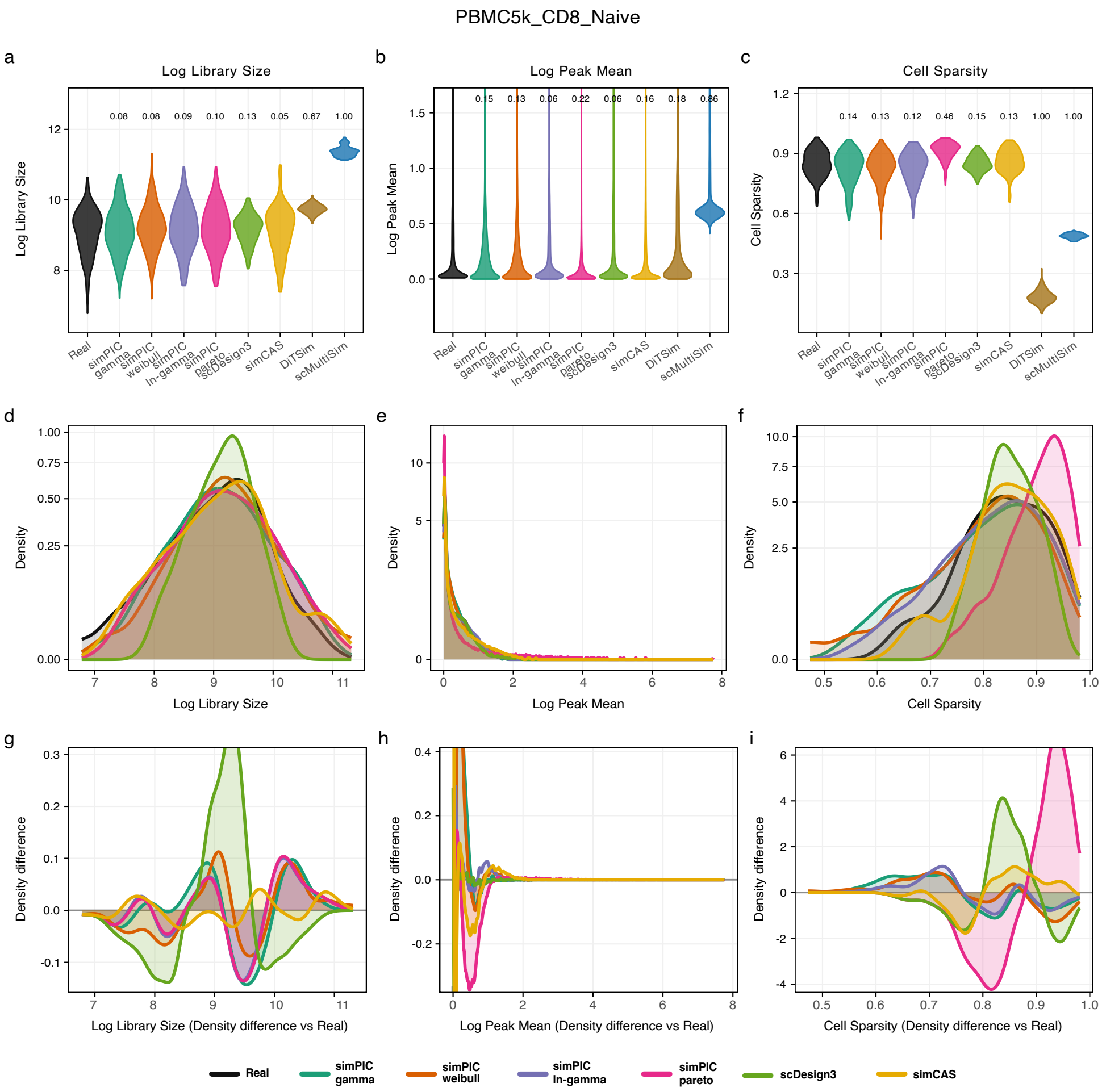

PBMC5k_CD8_Naive
a
Log Library Size
b
Log Peak Mean
c
Cell Sparsity
Log Library Size
Log Peak Mean
Cell Sparsity
Real
simPIC gamma
simPIC weibull
simPIC ln-gamma
simPIC pareto
scDesign3
simCAS
DiTSim
scMultiSim
d
e
f
Density
Log Library Size
Log Peak Mean
Cell Sparsity
g
h
i
Density difference
Log Library Size (Density difference vs Real)
Log Peak Mean (Density difference vs Real)
Cell Sparsity (Density difference vs Real)

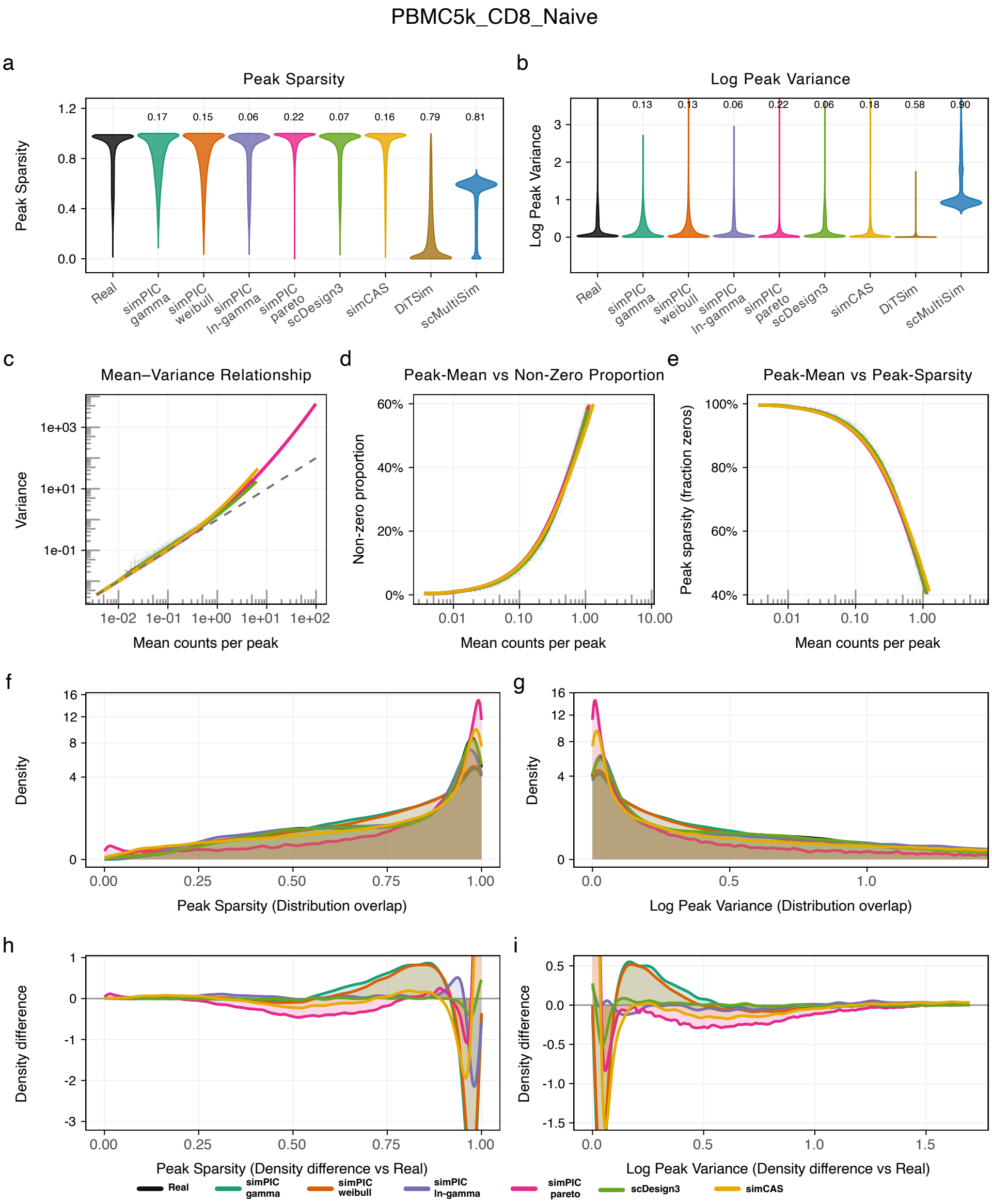

PBMC5k_CD8_Naive
a
Peak Sparsity
Peak Sparsity
0.17
0.15
0.06
0.22
0.07
0.16
0.79
0.81
Real
simPIC gamma
simPIC weibull
simPIC ln-gamma
simPIC pareto
scDesign3
simCAS
DiTSim
scMultiSim
b
Log Peak Variance
Log Peak Variance
0.13
0.13
0.06
0.22
0.06
0.18
0.58
0.90
c
Mean–Variance Relationship
Variance
Mean counts per peak
d
Peak-Mean vs Non-Zero Proportion
Non-zero proportion
Mean counts per peak
e
Peak-Mean vs Peak-Sparsity
Peak sparsity (fraction zeros)
Mean counts per peak
f
Density
Peak Sparsity (Distribution overlap)
g
Density
Log Peak Variance (Distribution overlap)
h
Density difference
Peak Sparsity (Density difference vs Real)
i
Density difference
Log Peak Variance (Density difference vs Real)
Real
simPIC gamma
simPIC weibull
simPIC ln-gamma
simPIC pareto
scDesign3
simCAS

PBMC5k_CD14_Monocytes

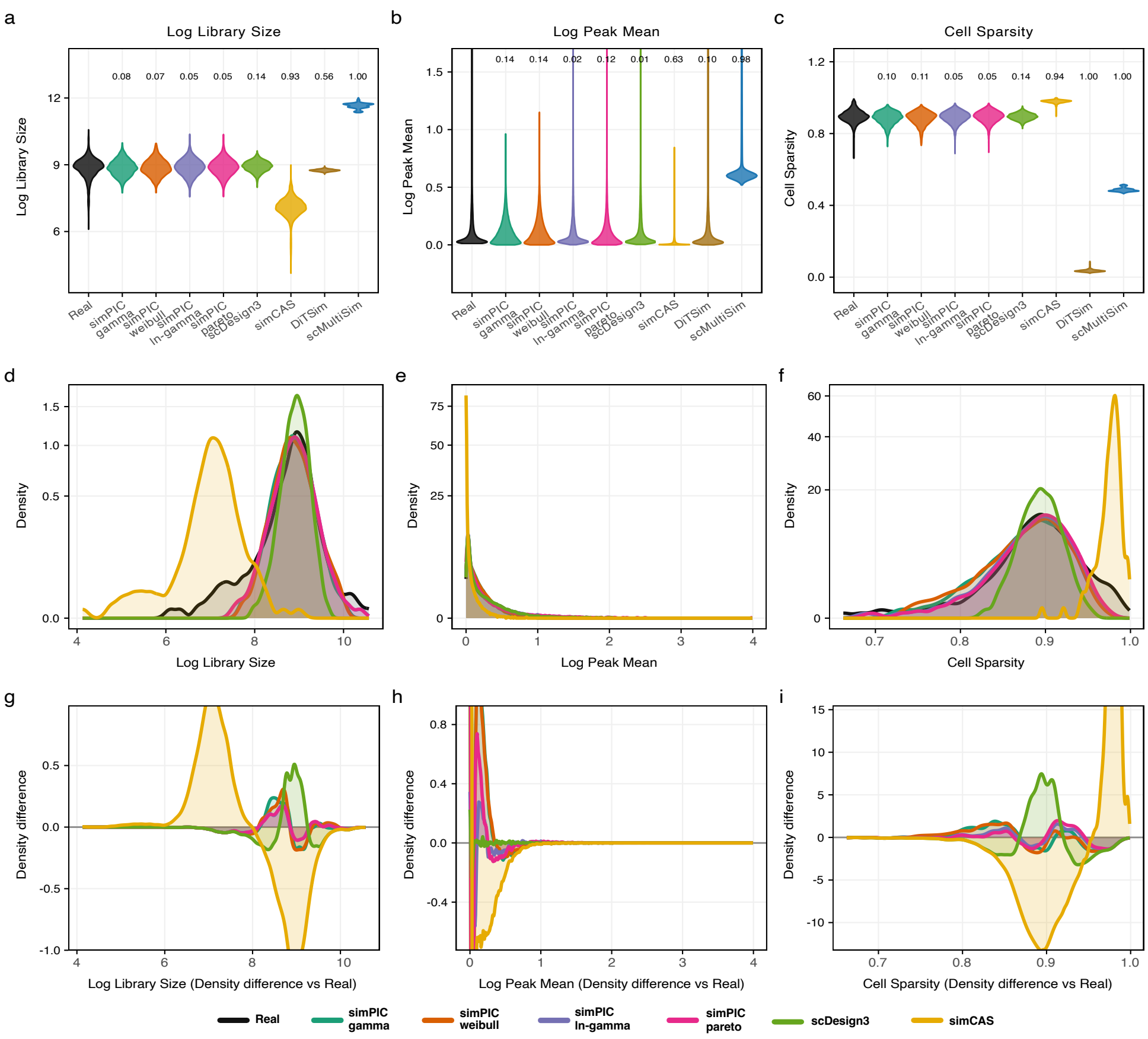

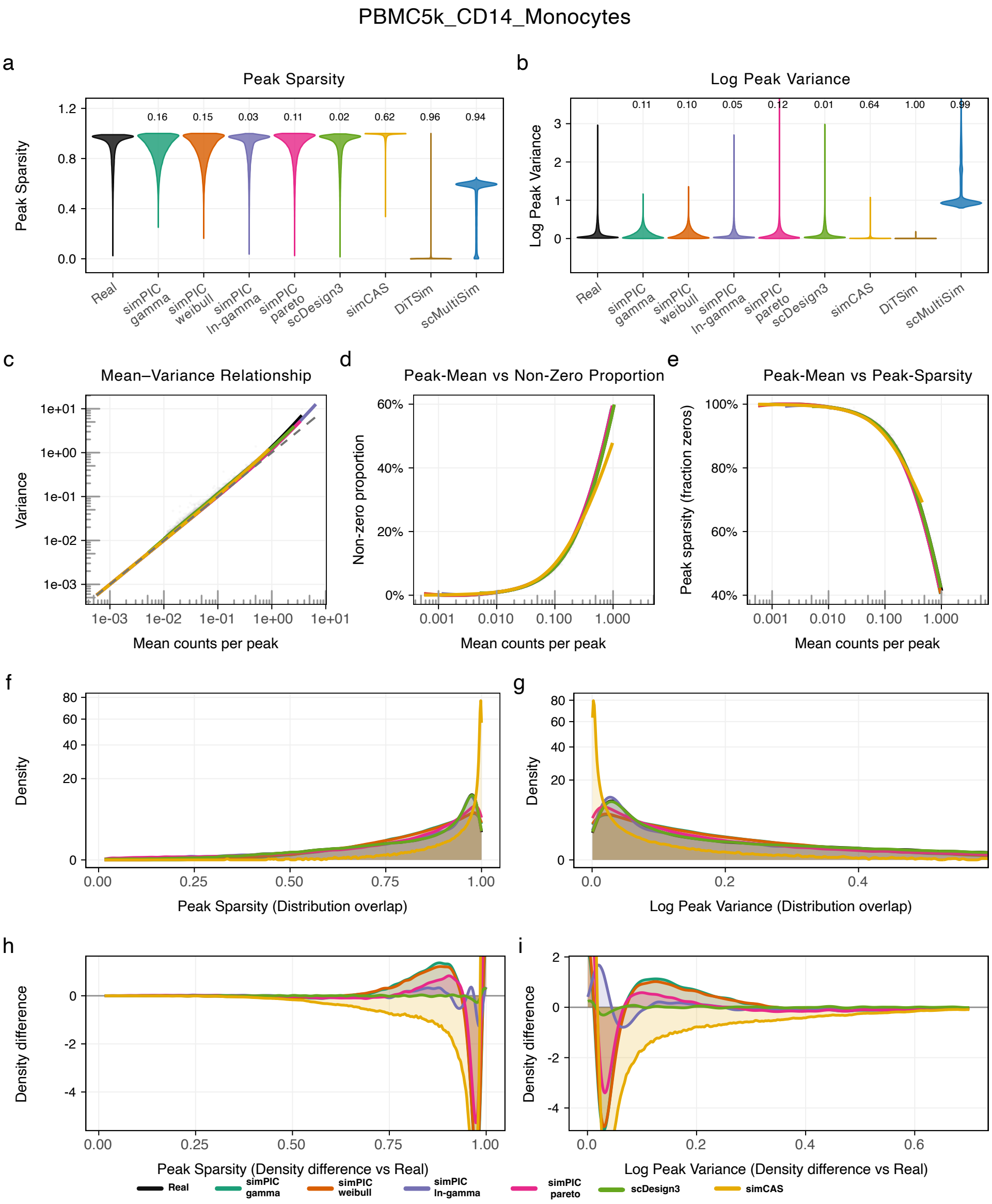
PBMC5k_CD14_Monocytes
a
Peak Sparsity
Peak Sparsity
0.16 0.15 0.03 0.11 0.02 0.62 0.96 0.94
Real
simPIC gamma
simPIC weibull
simPIC ln-gamma
simPIC pareto
scDesign3
simCAS
DiTSim
scMultiSim
b
Log Peak Variance
Log Peak Variance
0.11 0.10 0.05 0.12 0.01 0.64 1.00 0.99
c
Mean–Variance Relationship
Variance
Mean counts per peak
d
Peak-Mean vs Non-Zero Proportion
Non-zero proportion
Mean counts per peak
e
Peak-Mean vs Peak-Sparsity
Peak sparsity (fraction zeros)
Mean counts per peak
f
Density
Peak Sparsity (Distribution overlap)
g
Density
Log Peak Variance (Distribution overlap)
h
Density difference
Peak Sparsity (Density difference vs Real)
i
Density difference
Log Peak Variance (Density difference vs Real)
Real
simPIC gamma
simPIC weibull
simPIC ln-gamma
simPIC pareto
scDesign3
simCAS

PBMC5k_Double_Negative_T_cell

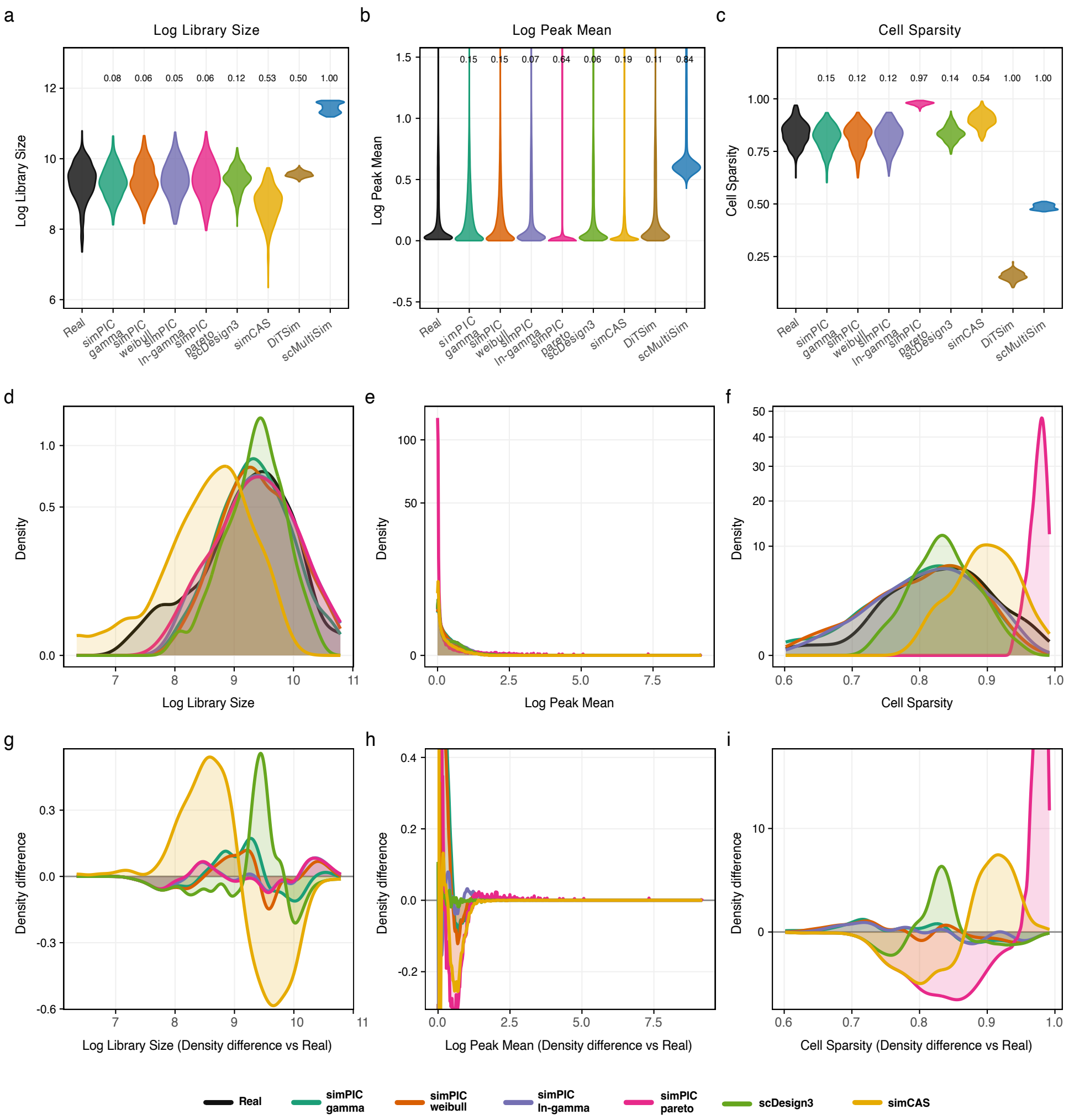

PBMC5k_Double_Negative_T_cell

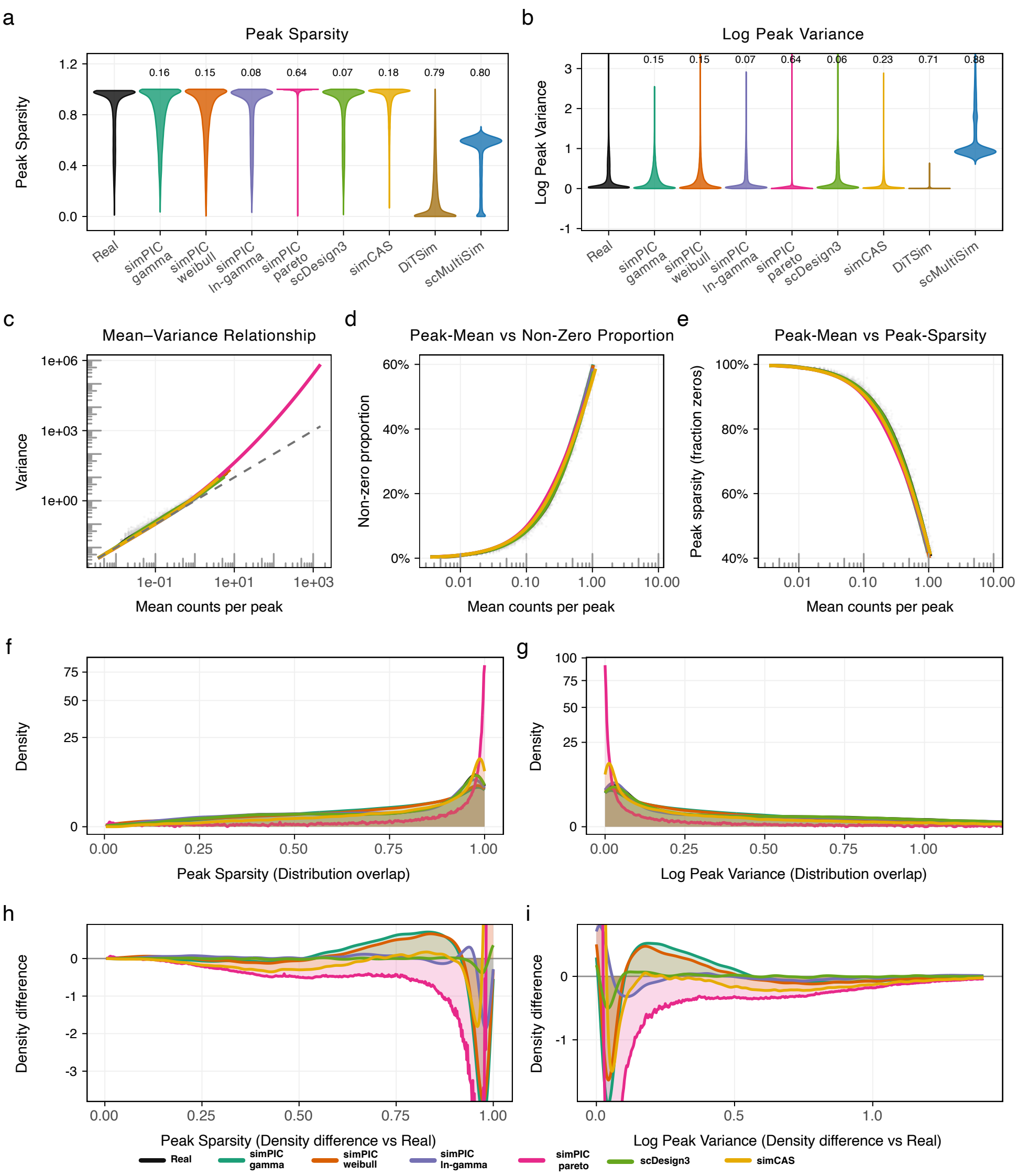

PBMC10k_CD14_Monocytes

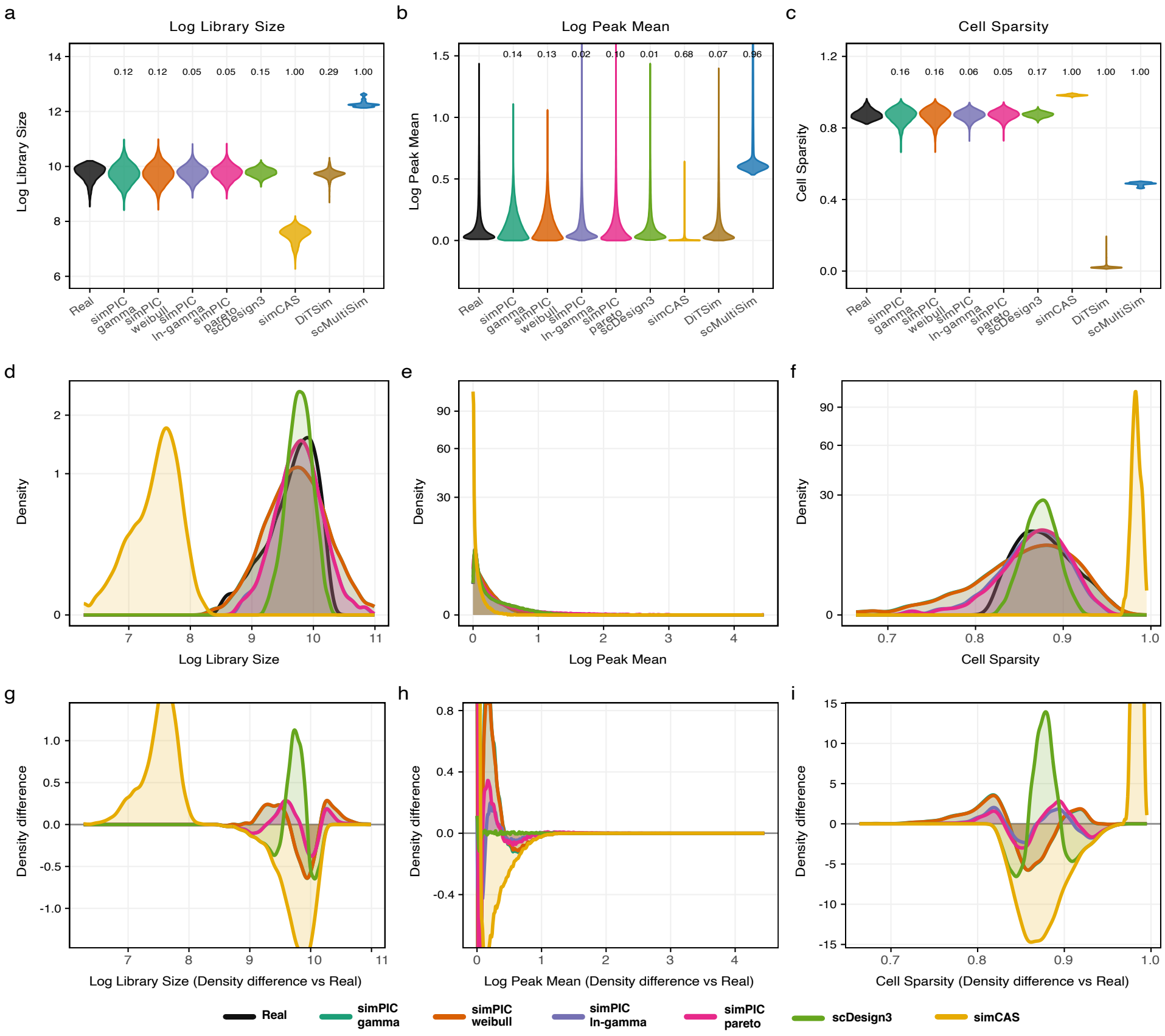

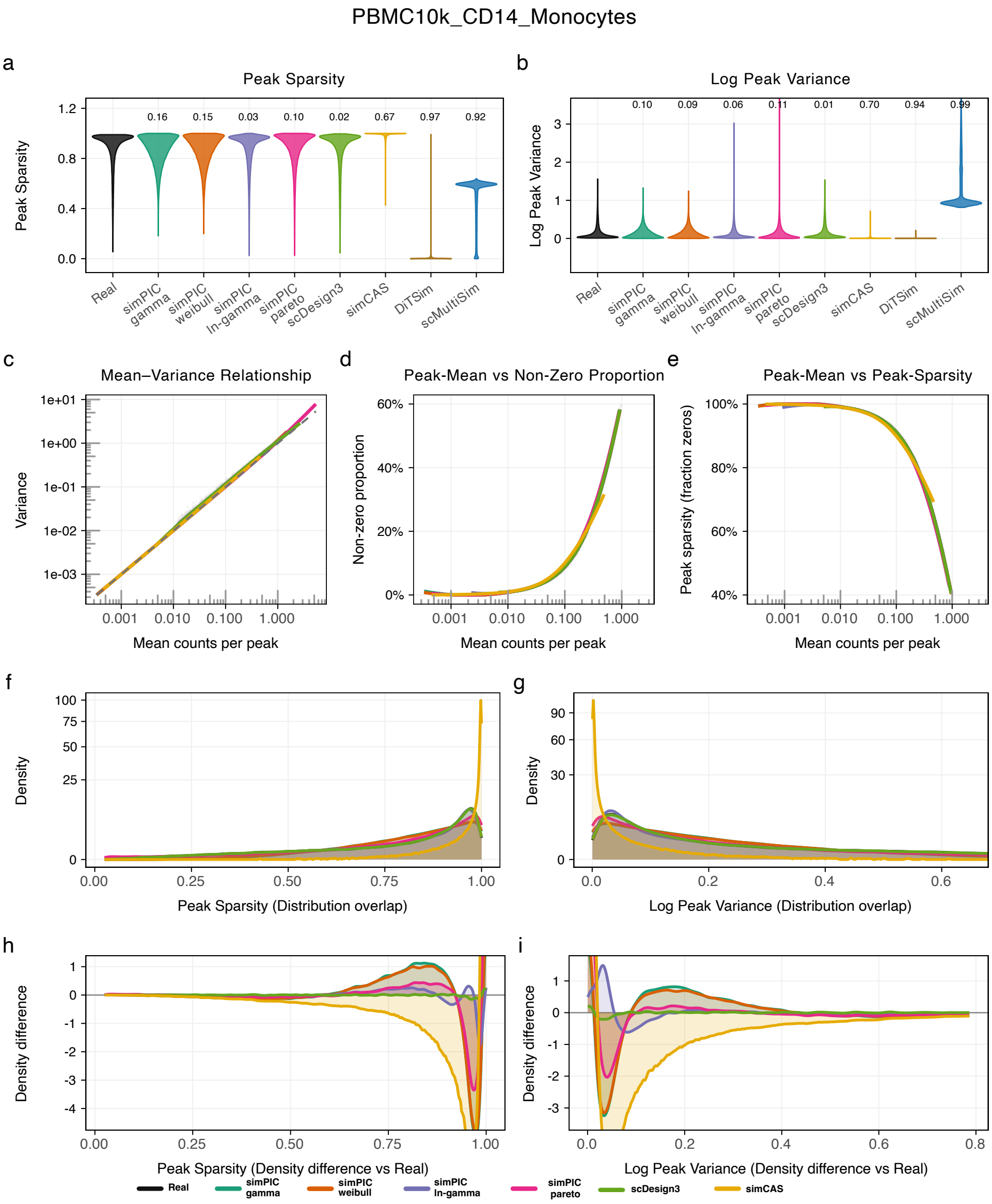

PBMC10k_CD14_Monocytes
a
Peak Sparsity
b
Log Peak Variance
Real
simPIC gamma
simPIC weibull
simPIC ln-gamma
simPIC pareto
scDesign3
simCAS
DiTSim
scMultiSim
c
Mean–Variance Relationship
Variance
Mean counts per peak
d
Peak-Mean vs Non-Zero Proportion
Non-zero proportion
e
Peak-Mean vs Peak-Sparsity
Peak sparsity (fraction zeros)
f
Peak Sparsity (Distribution overlap)
g
Log Peak Variance (Distribution overlap)
Density
h
Peak Sparsity (Density difference vs Real)
i
Log Peak Variance (Density difference vs Real)
Density difference

PBMC10k_CD4_Memory

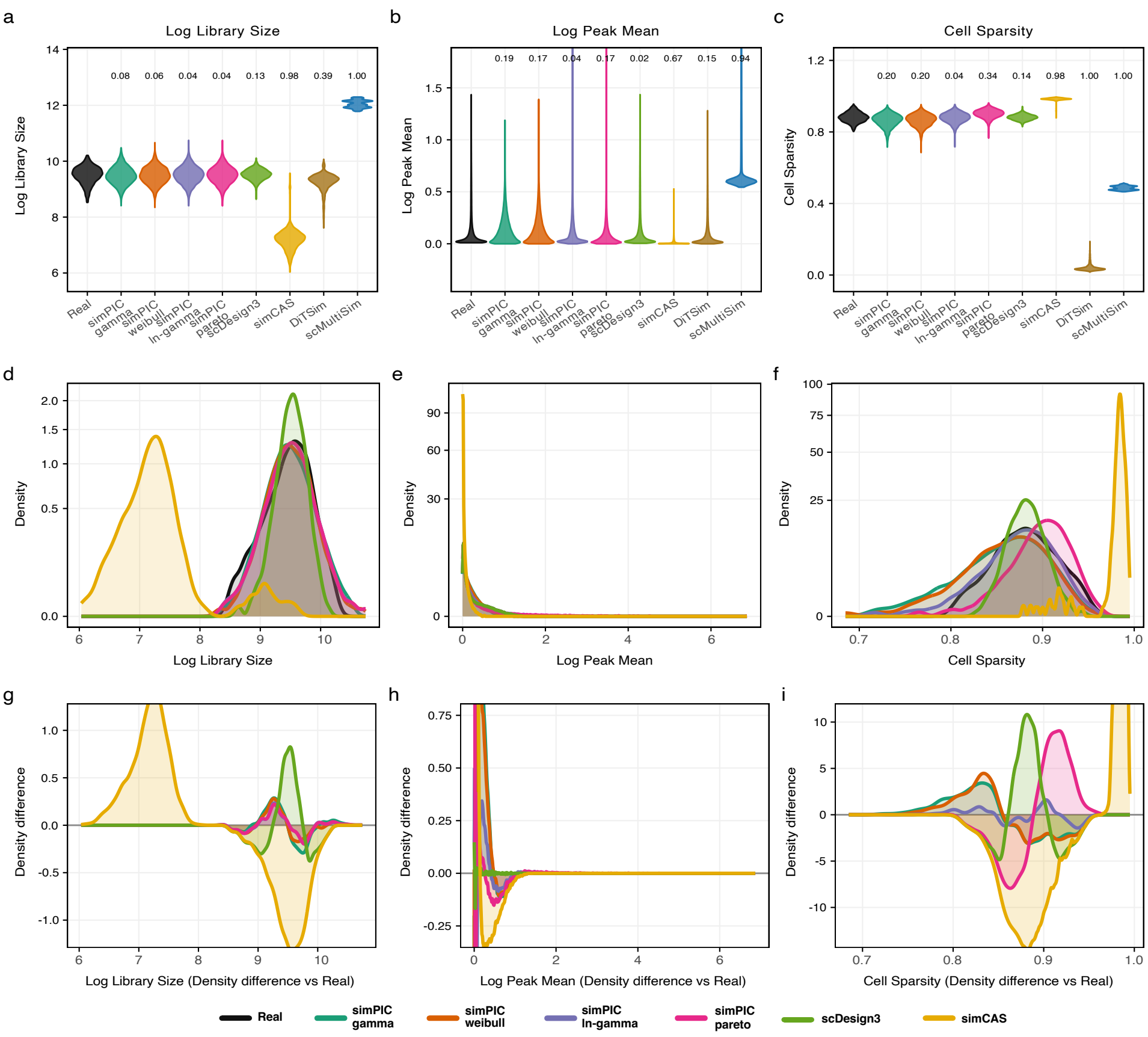

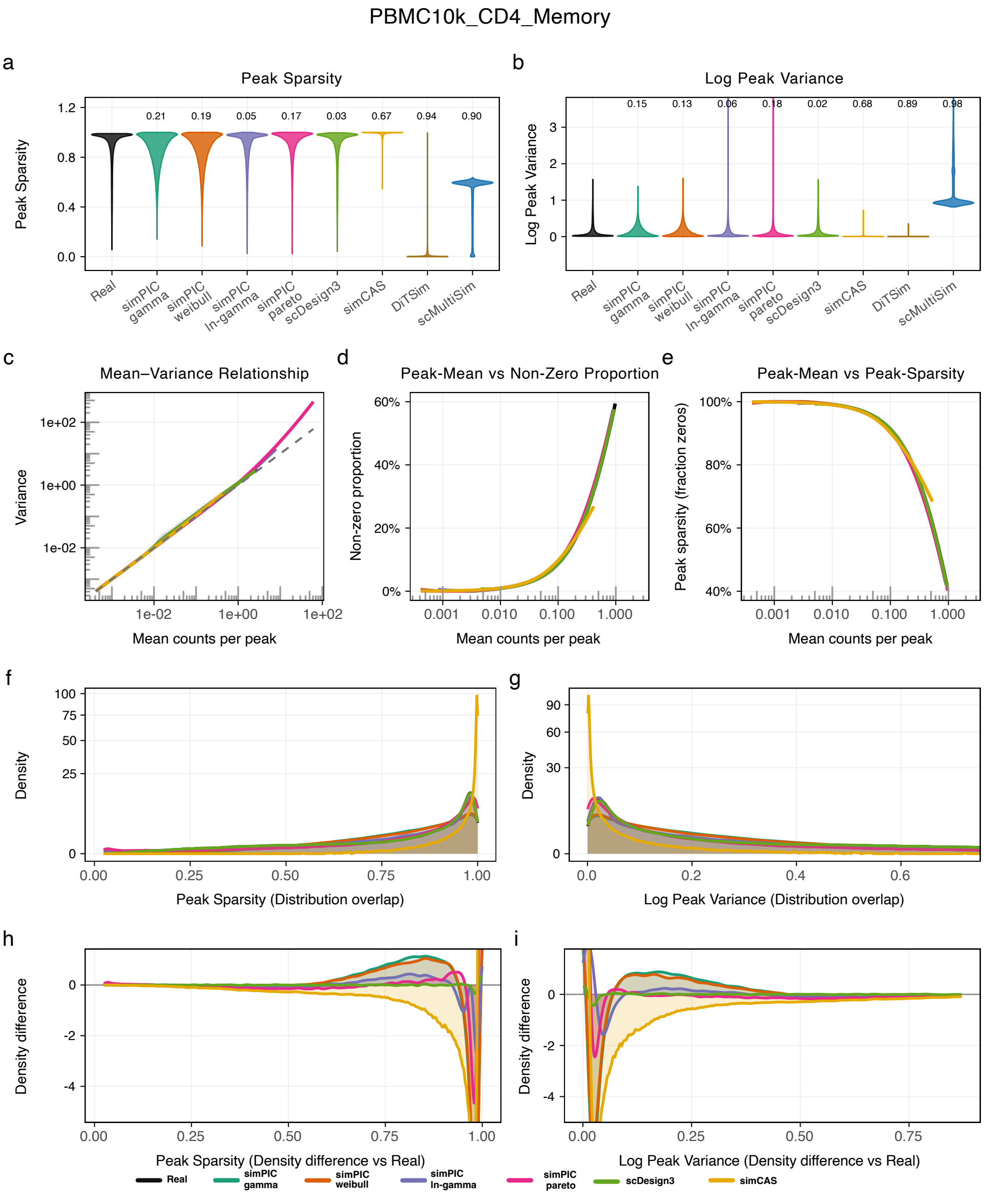

PBMC10k_CD4_Memory
a
Peak Sparsity
Peak Sparsity
0.21 0.19 0.05 0.17 0.03 0.67 0.94 0.90
Real
simPIC gamma
simPIC weibull
simPIC ln-gamma
simPIC pareto
scDesign3
simCAS
DiTSim
scMultiSim
b
Log Peak Variance
Log Peak Variance
0.15 0.13 0.06 0.18 0.02 0.68 0.89 0.98
c
Mean–Variance Relationship
Variance
Mean counts per peak
d
Peak-Mean vs Non-Zero Proportion
Non-zero proportion
Mean counts per peak
e
Peak-Mean vs Peak-Sparsity
Peak sparsity (fraction zeros)
Mean counts per peak
f
Density
Peak Sparsity (Distribution overlap)
g
Density
Log Peak Variance (Distribution overlap)
h
Density difference
Peak Sparsity (Density difference vs Real)
i
Density difference
Log Peak Variance (Density difference vs Real)
Real
simPIC gamma
simPIC weibull
simPIC ln-gamma
simPIC pareto
scDesign3
simCAS

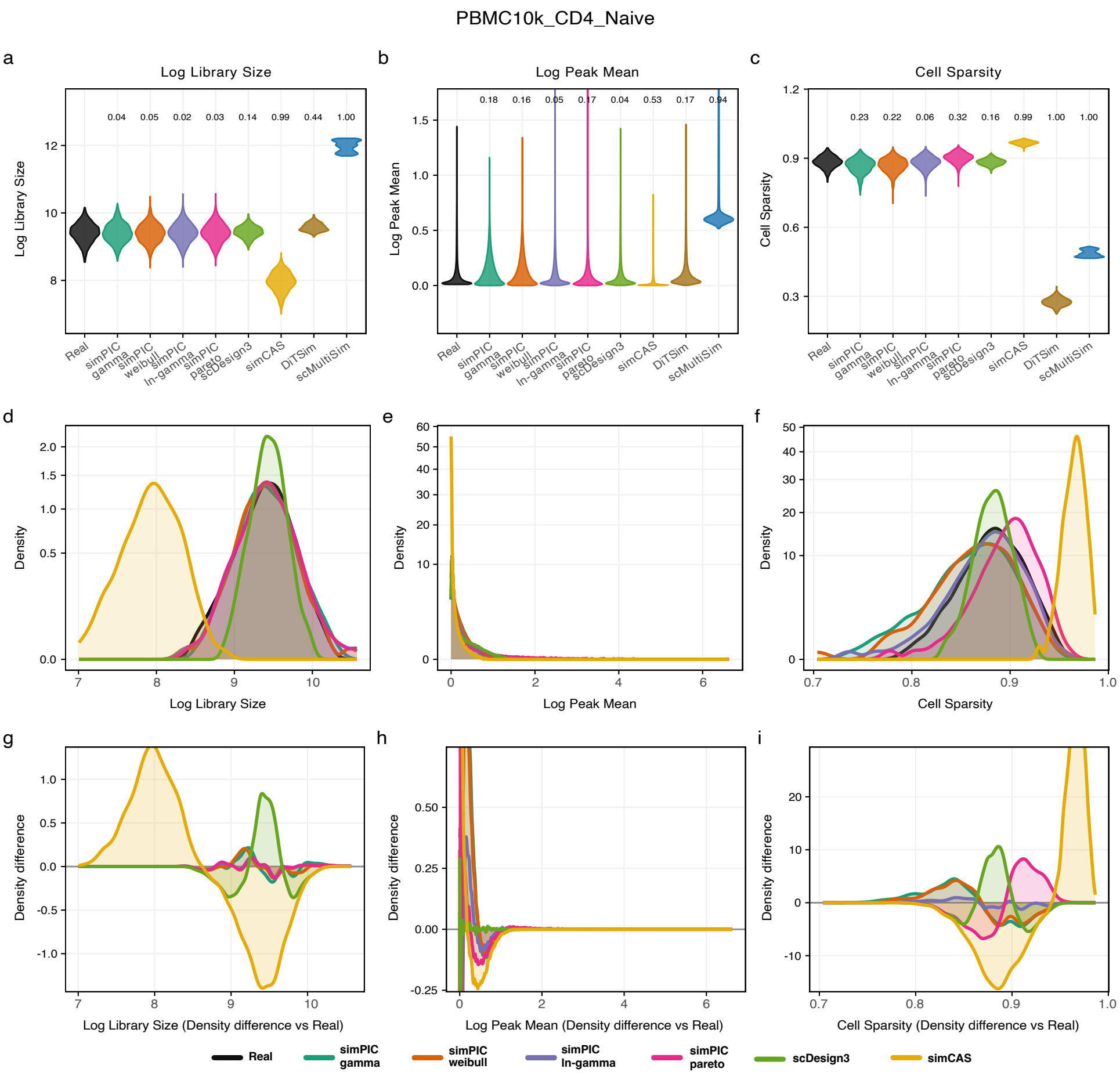
PBMC10k_CD4_Naive
a
Log Library Size
b
Log Peak Mean
c
Cell Sparsity
d
e
f
g
h
i
Log Library Size
Log Peak Mean
Cell Sparsity
Density
Density difference
Log Library Size (Density difference vs Real)
Log Peak Mean (Density difference vs Real)
Cell Sparsity (Density difference vs Real)
Real
simPIC gamma
simPIC weibull
simPIC ln-gamma
simPIC pareto
scDesign3
simCAS
DiTSim
scMultiSim

PBMC10k_CD4_Naive

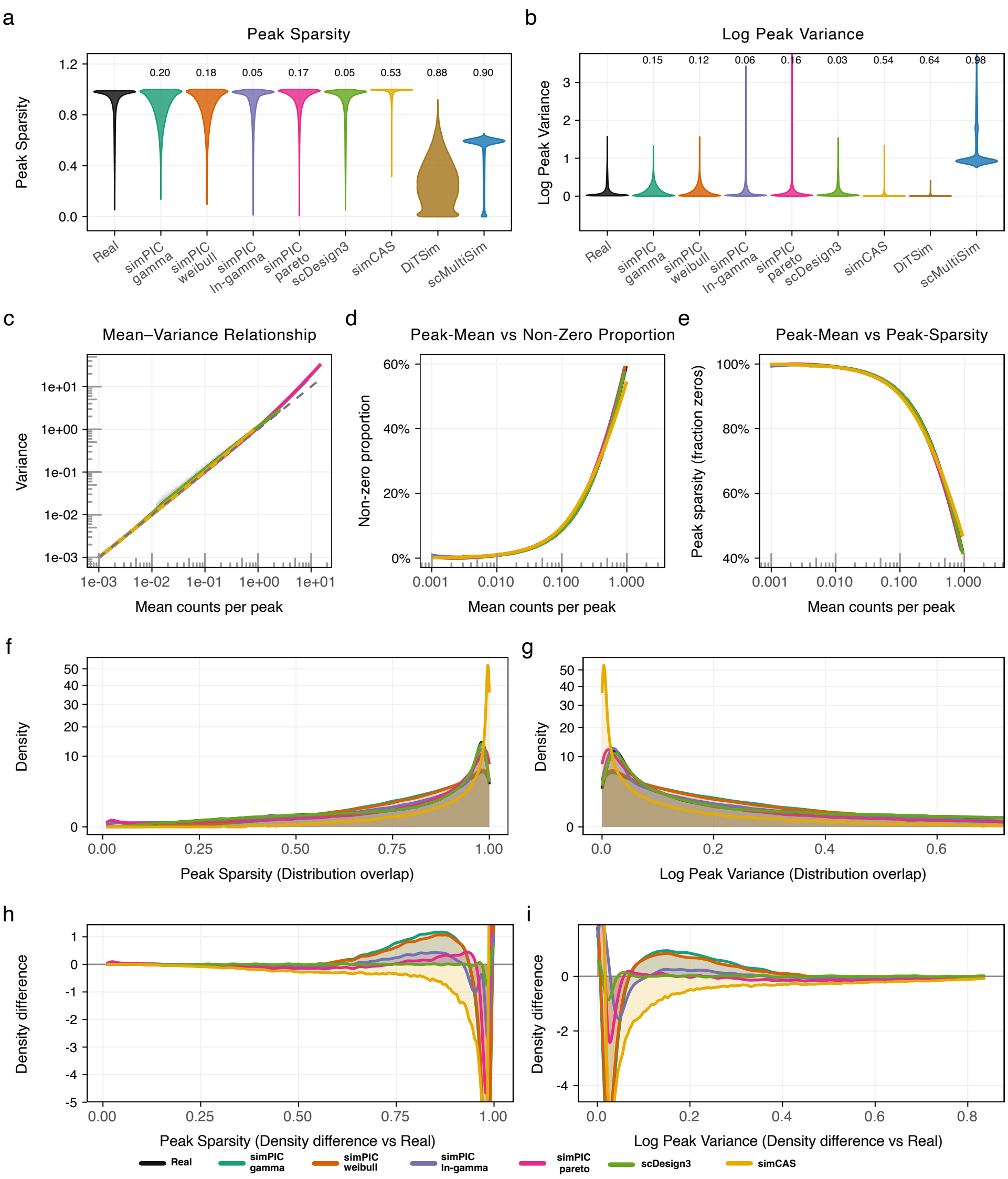

PBMC10k_CD8_naive

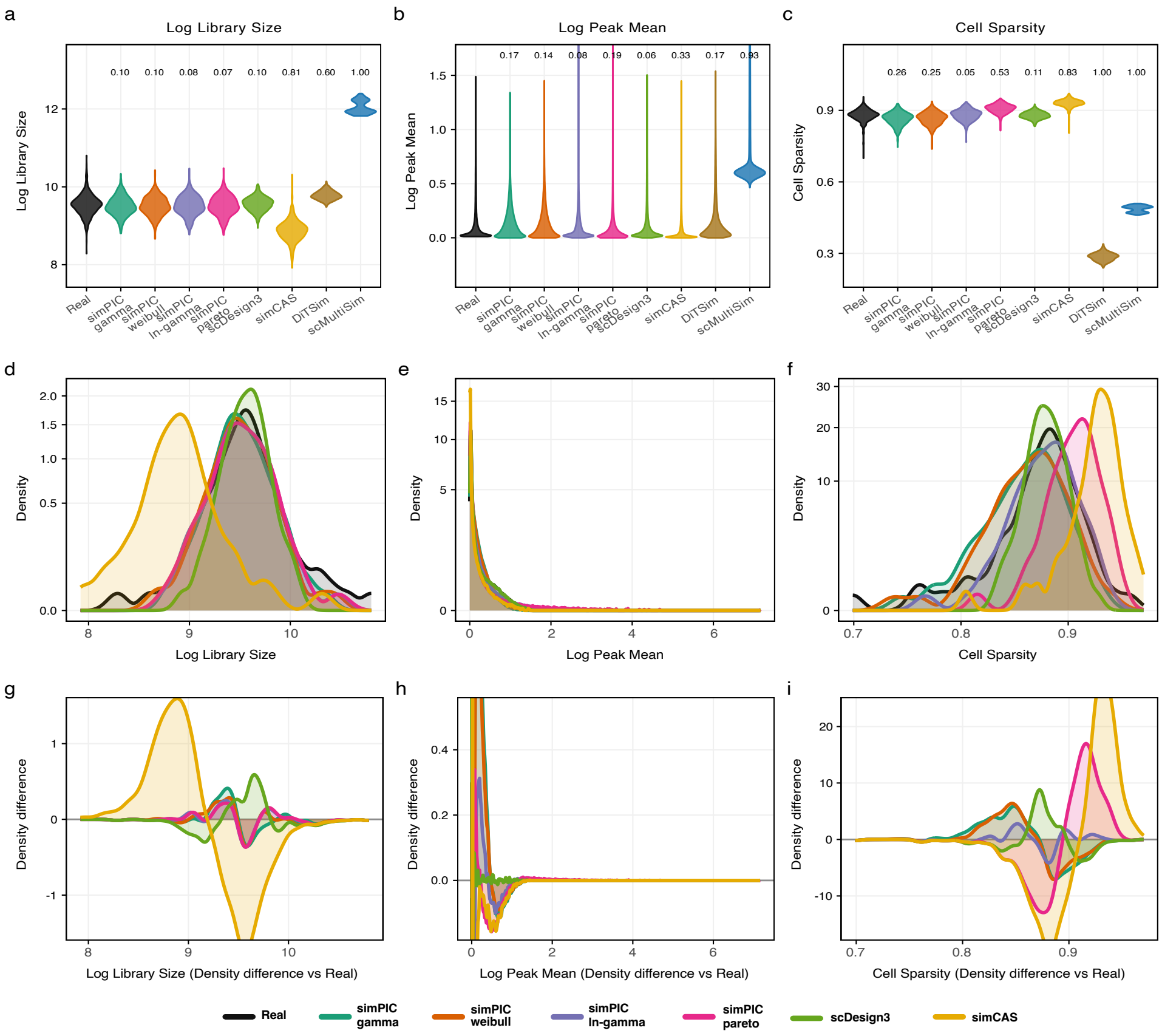

PBMC10k_CD8_naive

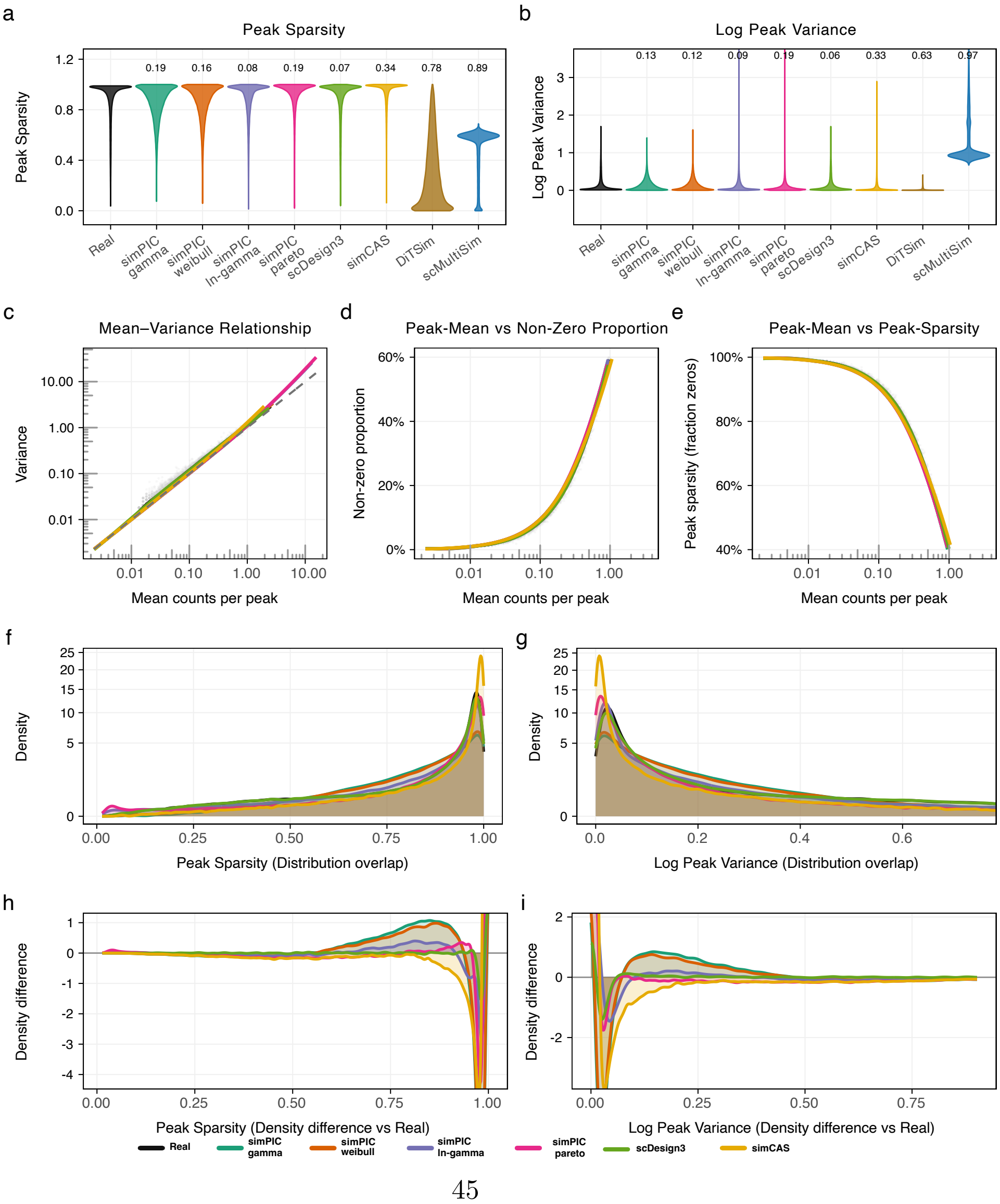

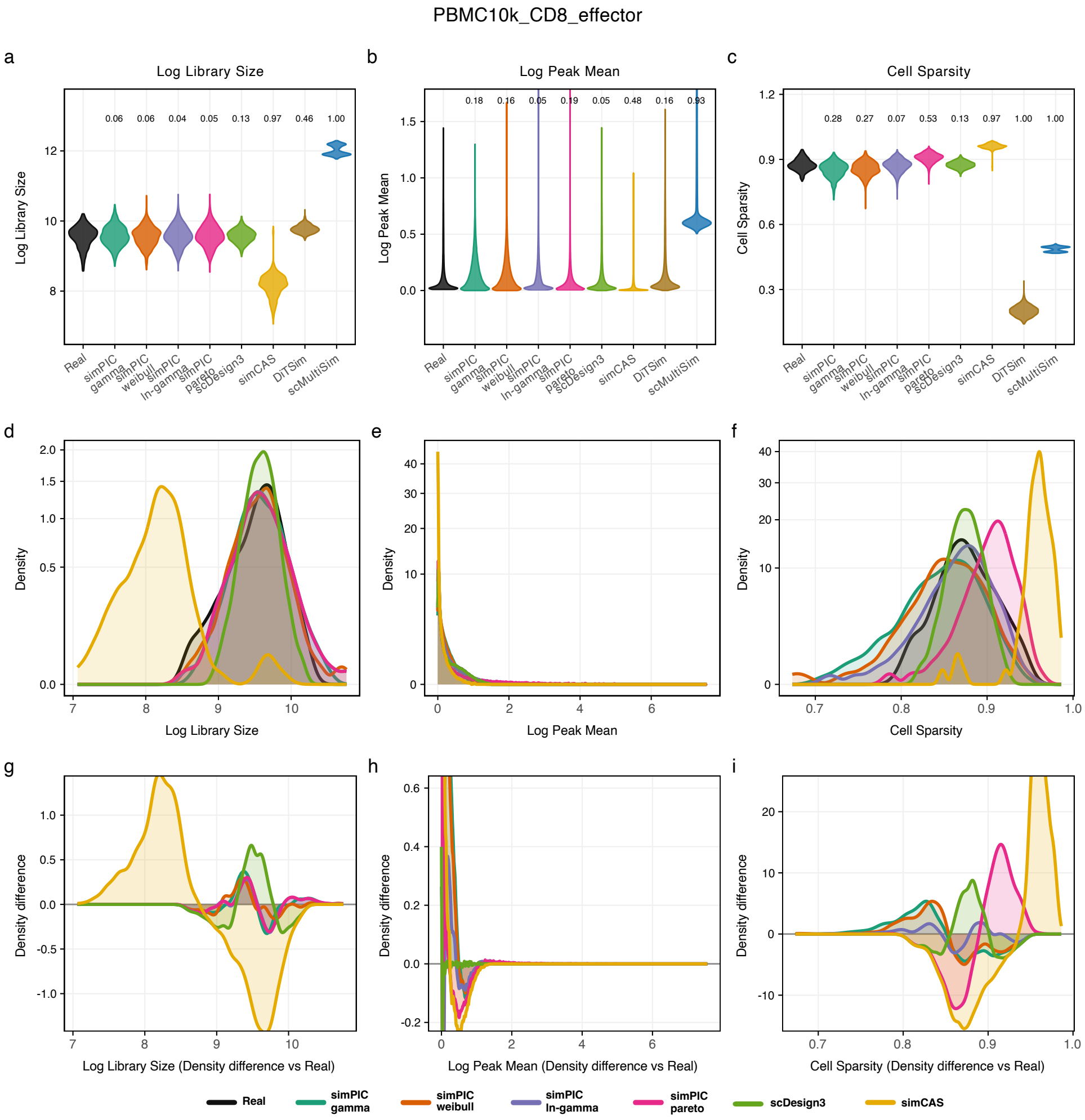
PBMC10k_CD8_effector
a
Log Library Size
b
Log Peak Mean
c
Cell Sparsity
Log Library Size
Log Peak Mean
Cell Sparsity
0.06 0.06 0.04 0.05 0.13 0.97 0.46 1.00
0.18 0.16 0.05 0.19 0.05 0.48 0.16 0.93
0.28 0.27 0.07 0.53 0.13 0.97 1.00 1.00
Real
simPIC gamma
simPIC weibull
simPIC ln-gamma
simPIC pareto
scDesign3
simCAS
DiTSim
scMultiSim
d
e
f
Density
Log Library Size
Log Peak Mean
Cell Sparsity
g
h
i
Density difference
Log Library Size (Density difference vs Real)
Log Peak Mean (Density difference vs Real)
Cell Sparsity (Density difference vs Real)
Real
simPIC gamma
simPIC weibull
simPIC ln-gamma
simPIC pareto
scDesign3
simCAS

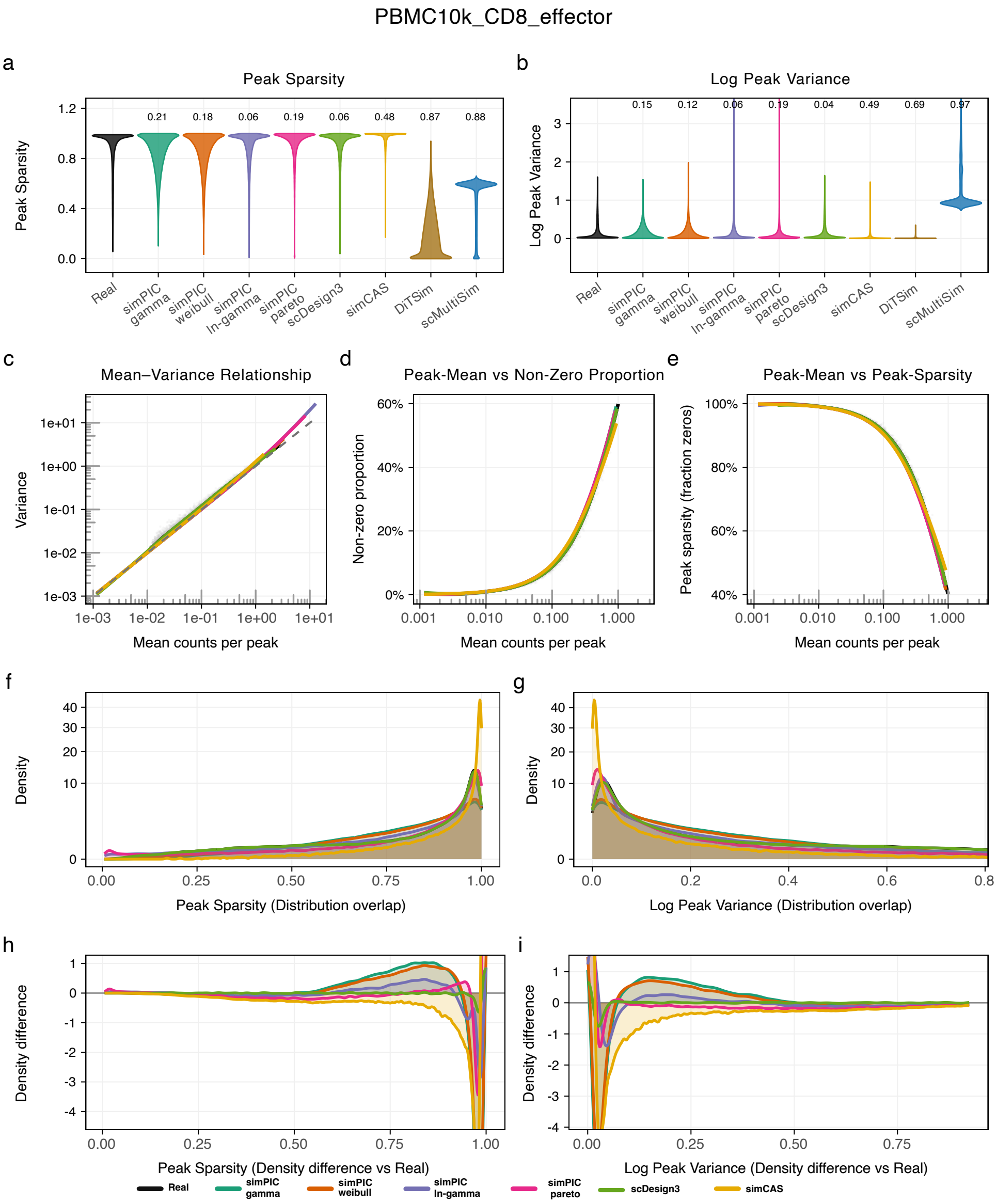
PBMC10k_CD8_effector
a
Peak Sparsity
Peak Sparsity
0.21
0.18
0.06
0.19
0.06
0.48
0.87
0.88
Real
simPIC gamma
simPIC weibull
simPIC ln-gamma
simPIC pareto
scDesign3
simCAS
DiTSim
scMultiSim
b
Log Peak Variance
Log Peak Variance
0.15
0.12
0.06
0.19
0.04
0.49
0.69
0.97
c
Mean–Variance Relationship
Variance
Mean counts per peak
d
Peak-Mean vs Non-Zero Proportion
Non-zero proportion
Mean counts per peak
e
Peak-Mean vs Peak-Sparsity
Peak sparsity (fraction zeros)
Mean counts per peak
f
Density
Peak Sparsity (Distribution overlap)
g
Density
Log Peak Variance (Distribution overlap)
h
Density difference
Peak Sparsity (Density difference vs Real)
i
Density difference
Log Peak Variance (Density difference vs Real)
Real
simPIC gamma
simPIC weibull
simPIC ln-gamma
simPIC pareto
scDesign3
simCAS

PBMC10k_B_cell_progenitor

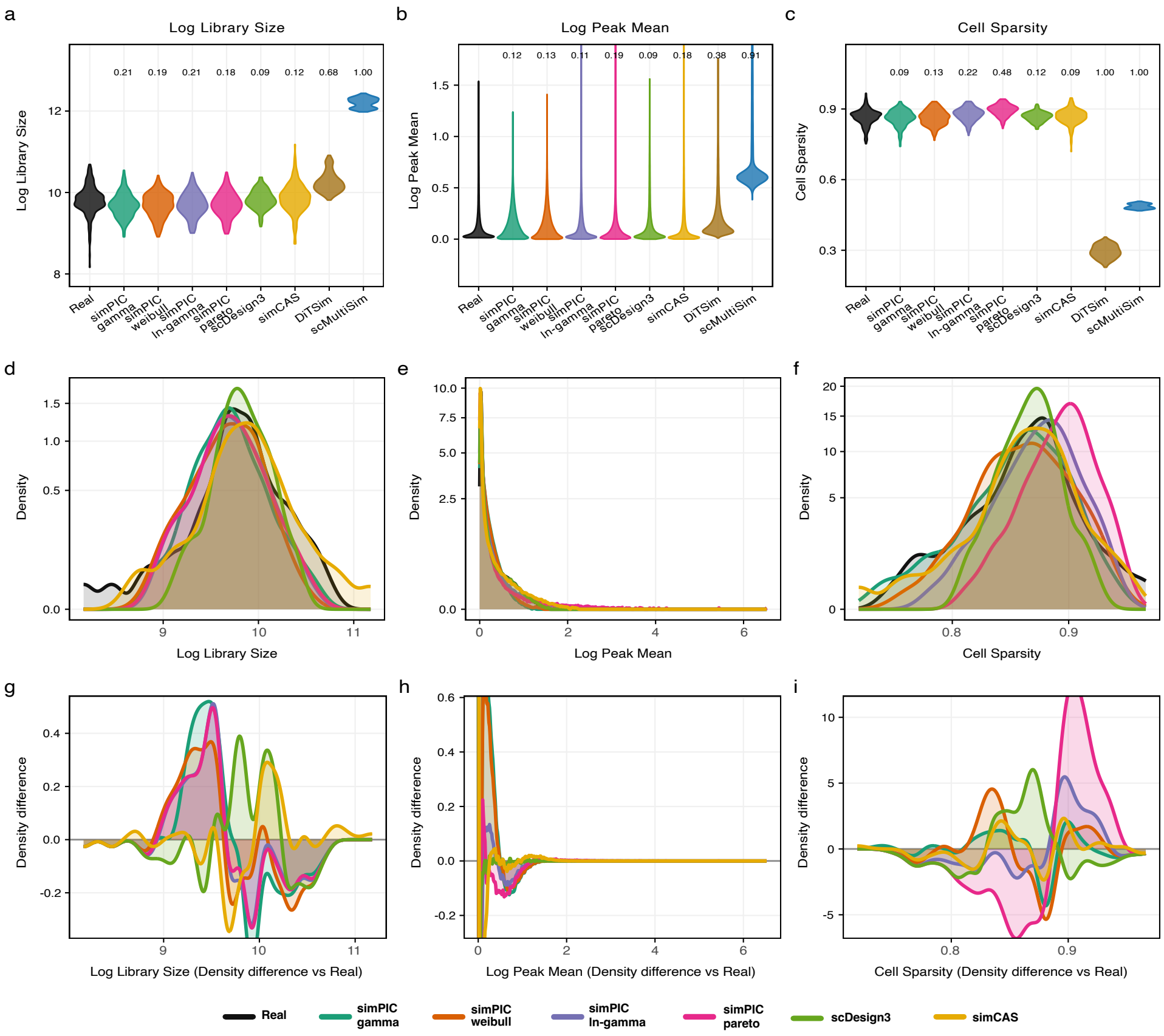

PBMC10k_B_cell_progenitor

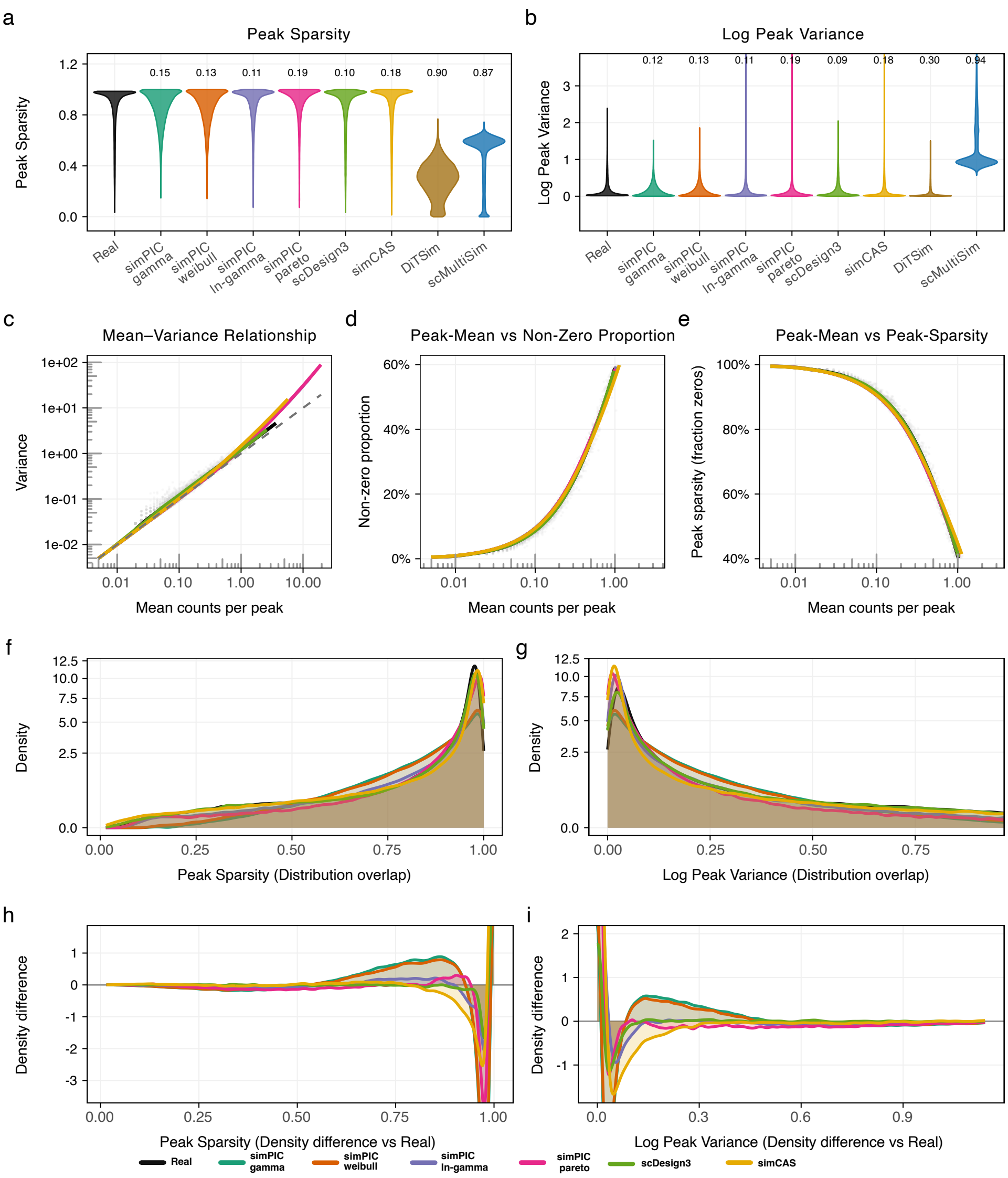

PBMC10k_NK_dim

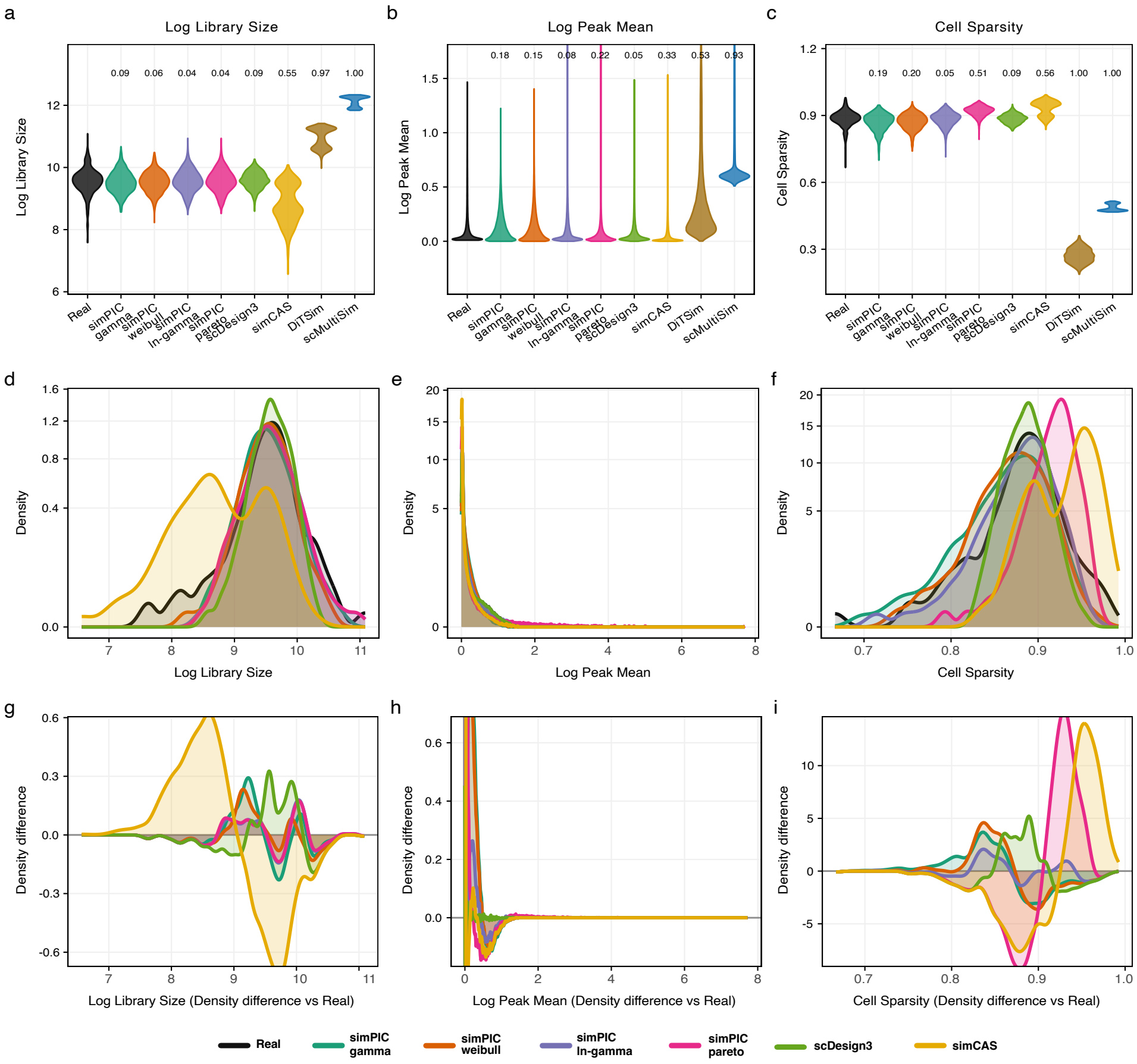

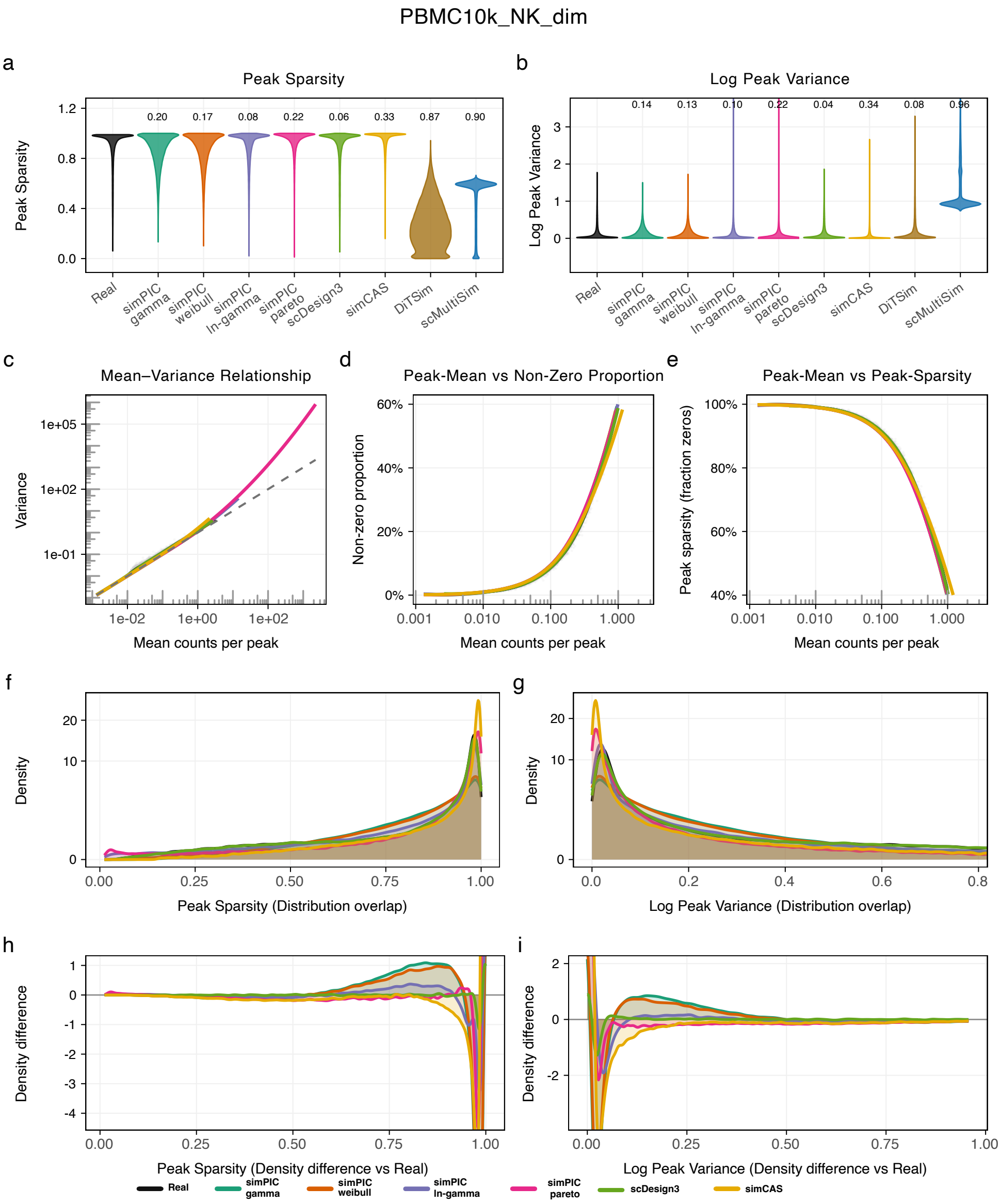

PBMC10k_NK_dim
a
Peak Sparsity
b
Log Peak Variance
c
Mean–Variance Relationship
d
Peak-Mean vs Non-Zero Proportion
e
Peak-Mean vs Peak-Sparsity
f
g
h
i
Peak Sparsity (Distribution overlap)
Log Peak Variance (Distribution overlap)
Peak Sparsity (Density difference vs Real)
Log Peak Variance (Density difference vs Real)
Real
simPIC gamma
simPIC weibull
simPIC ln-gamma
simPIC pareto
scDesign3
simCAS

Satpathy_CD8_Naive

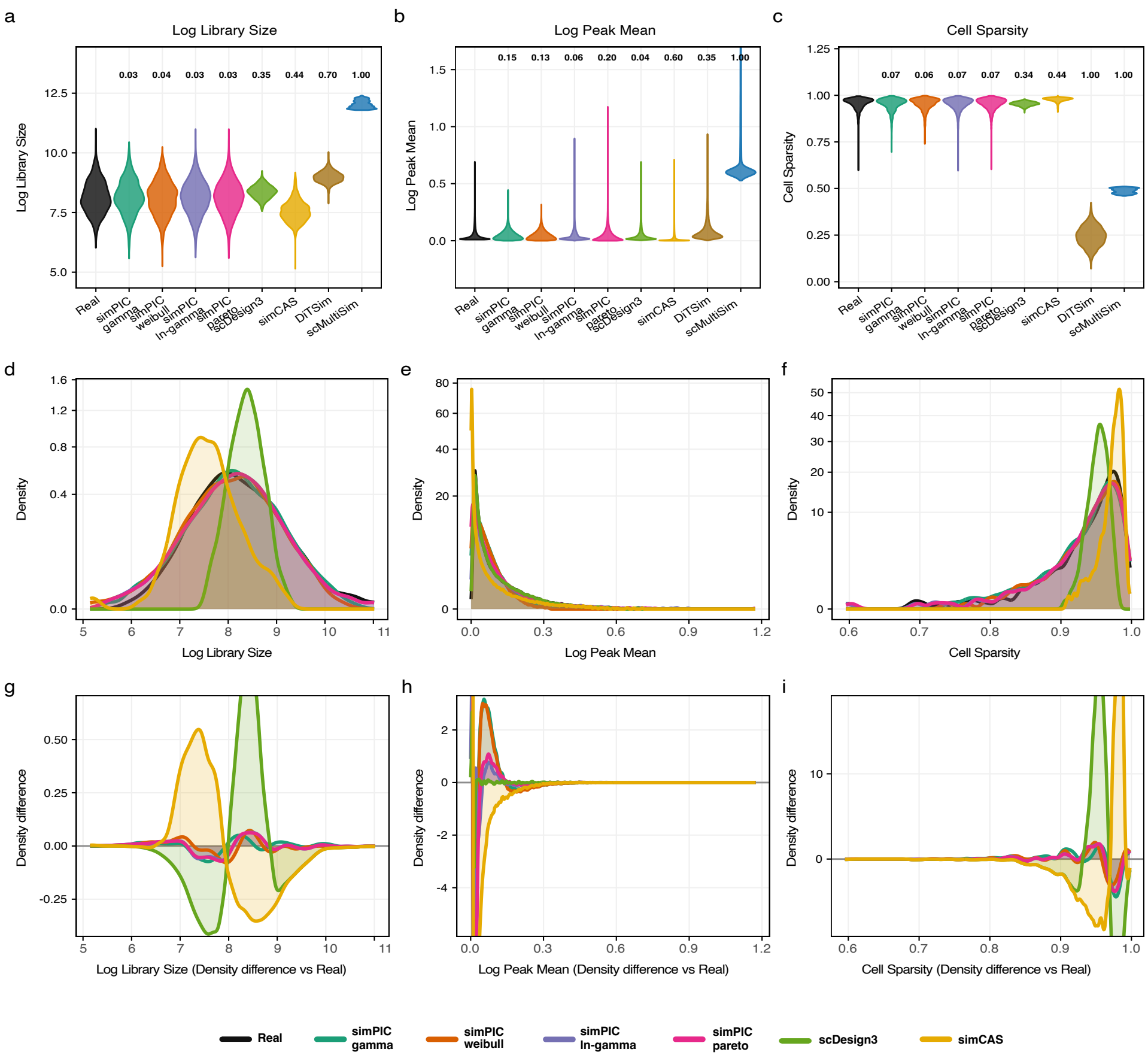

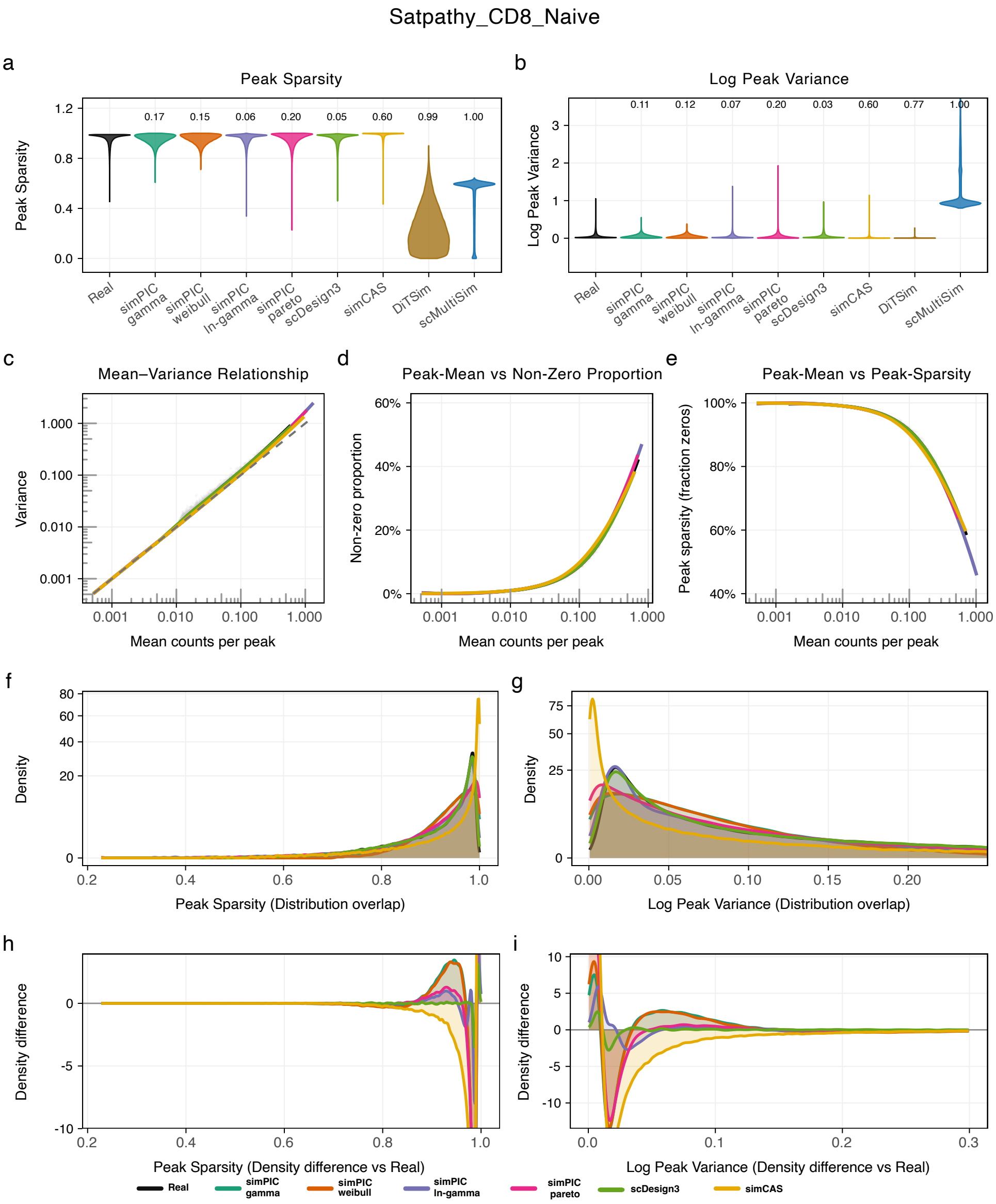

Satpathy_CD8_Naive
a
Peak Sparsity
b
Log Peak Variance
c
Mean–Variance Relationship
d
Peak-Mean vs Non-Zero Proportion
e
Peak-Mean vs Peak-Sparsity
f
g
h
i
Peak Sparsity
Log Peak Variance
Variance
Non-zero proportion
Peak sparsity (fraction zeros)
Mean counts per peak
Density
Peak Sparsity (Distribution overlap)
Log Peak Variance (Distribution overlap)
Density difference
Peak Sparsity (Density difference vs Real)
Log Peak Variance (Density difference vs Real)
Real
simPIC gamma
simPIC weibull
simPIC ln-gamma
simPIC pareto
scDesign3
simCAS
DiTSim
scMultiSim

Satpathy_CD34_Progenitors

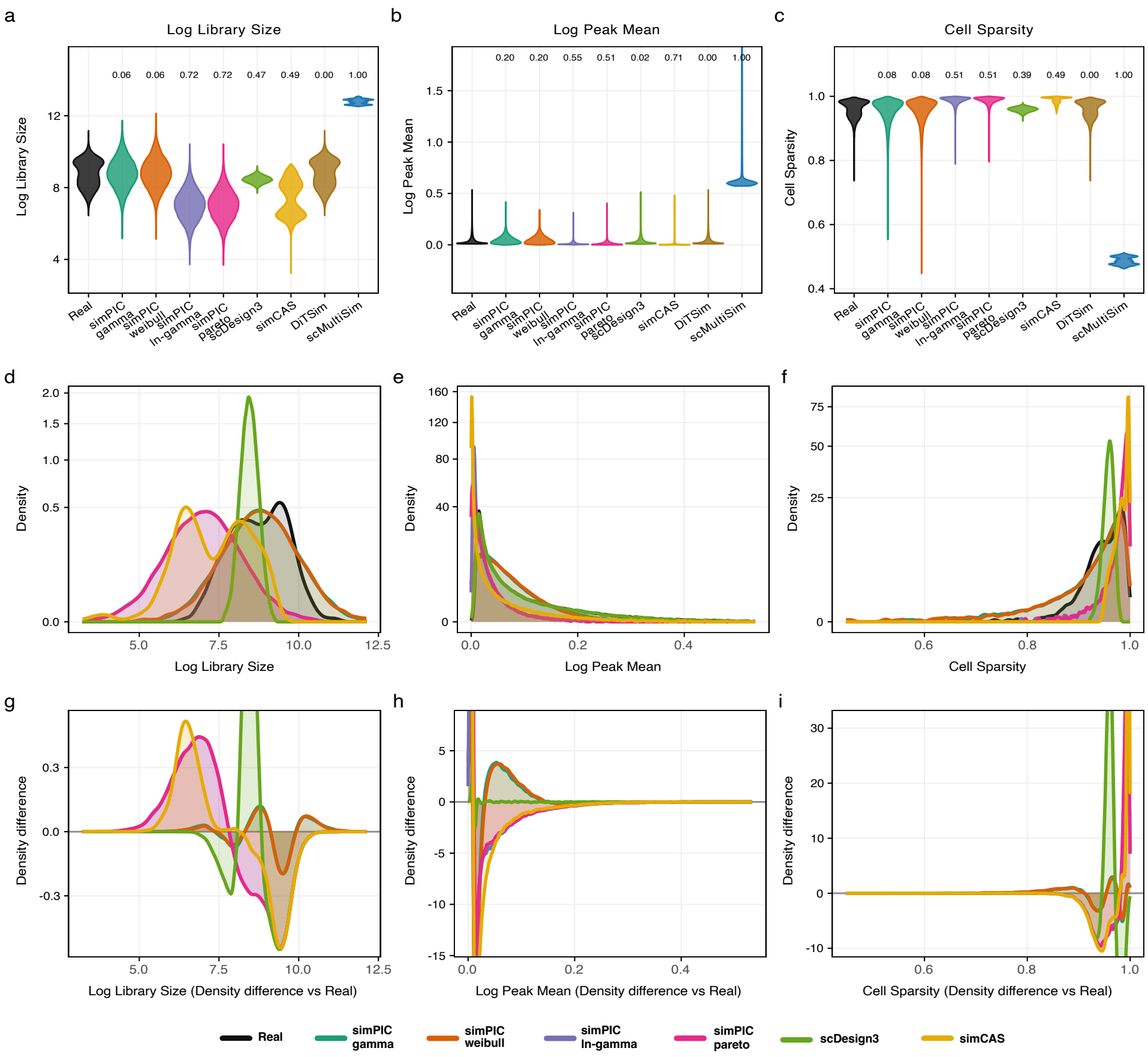

Satpathy_CD34_Progenitors

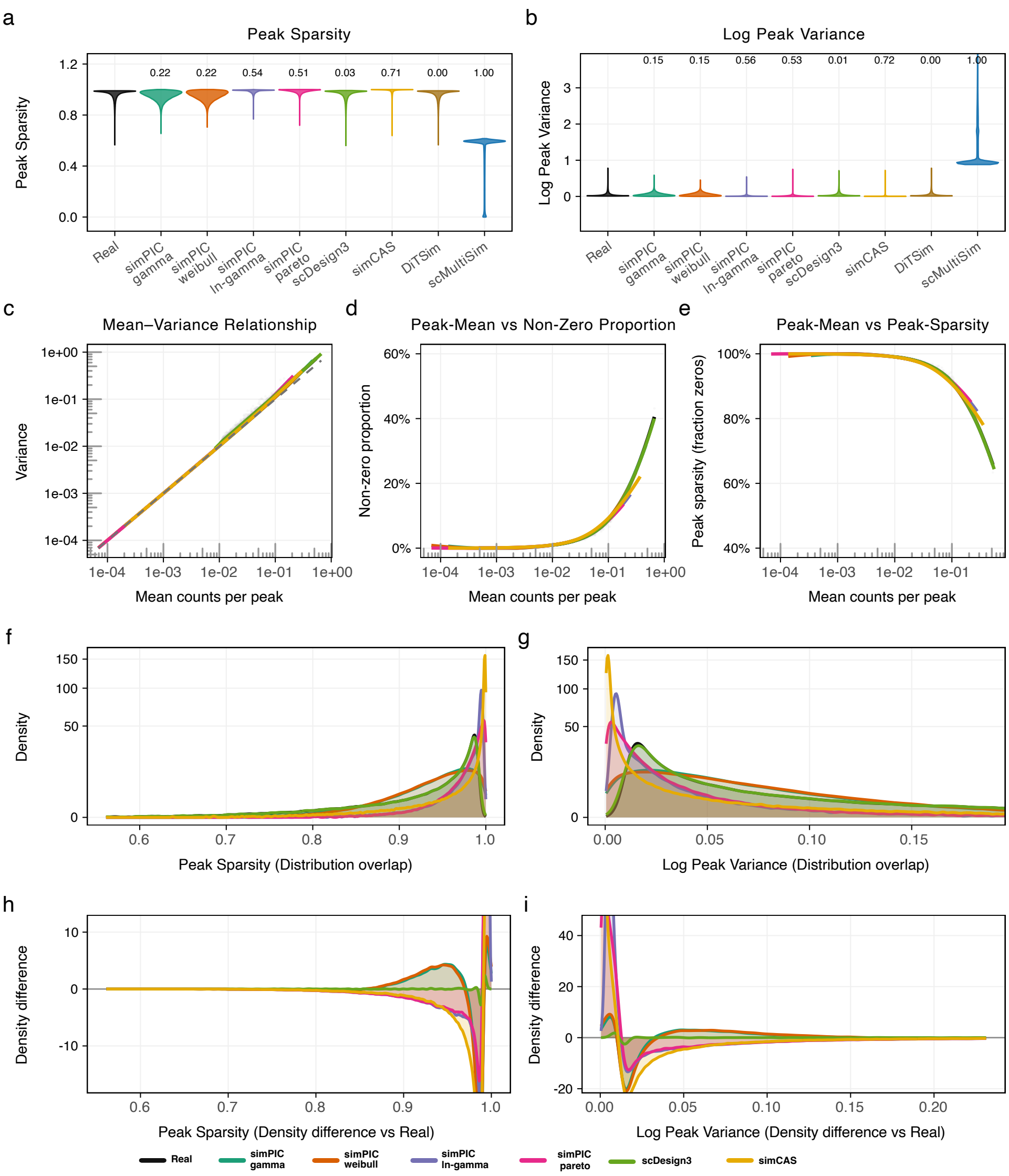

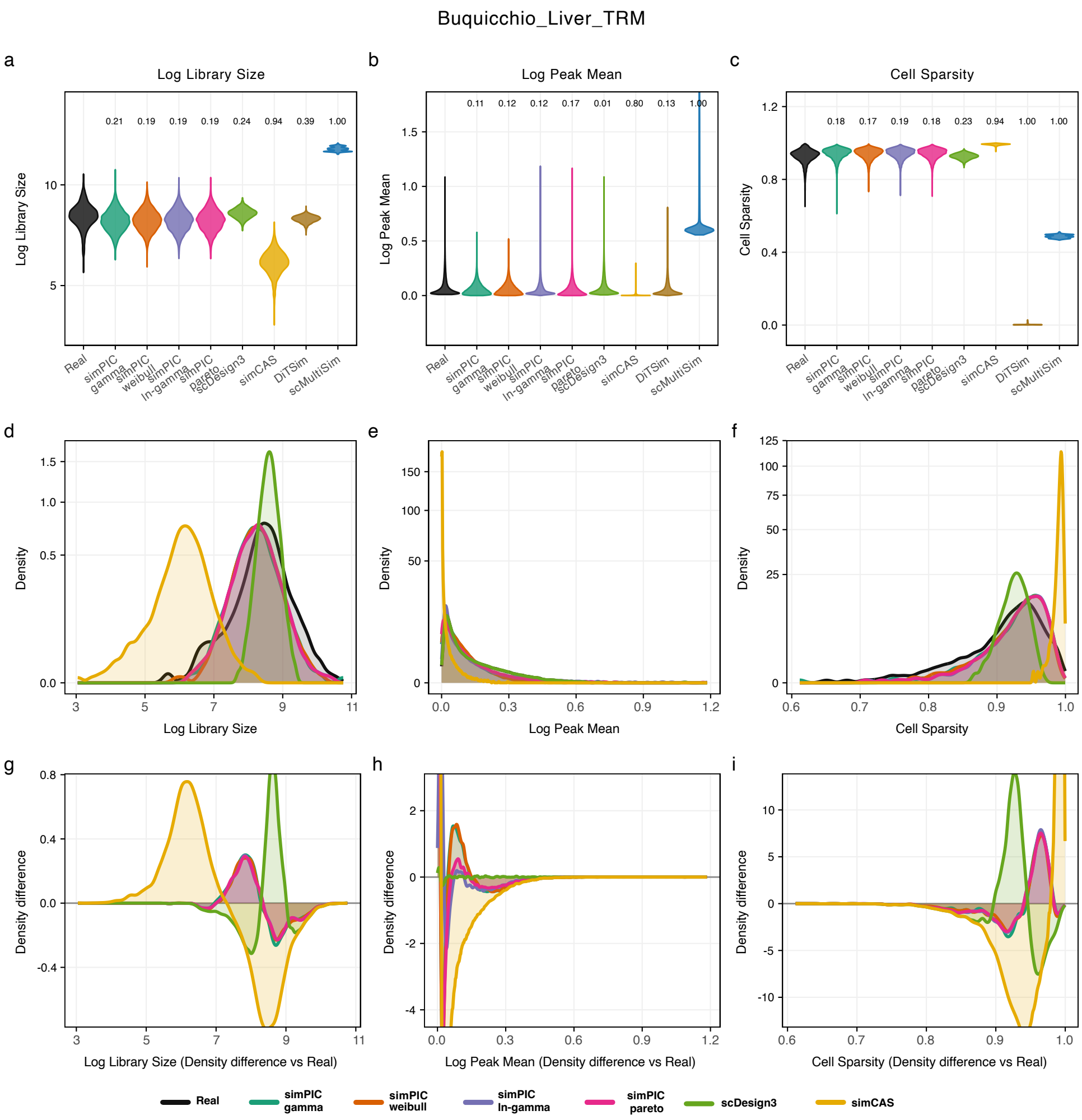
Buquicchio_Liver_TRM
a
Log Library Size
b
Log Peak Mean
c
Cell Sparsity
d
e
f
g
h
i
Log Library Size
Log Peak Mean
Cell Sparsity
Density
Density difference
Log Library Size (Density difference vs Real)
Log Peak Mean (Density difference vs Real)
Cell Sparsity (Density difference vs Real)
Real
simPIC gamma
simPIC weibull
simPIC ln-gamma
simPIC pareto
scDesign3
simCAS
DiTSim
scMultiSim

Buquicchio_Liver_TRM

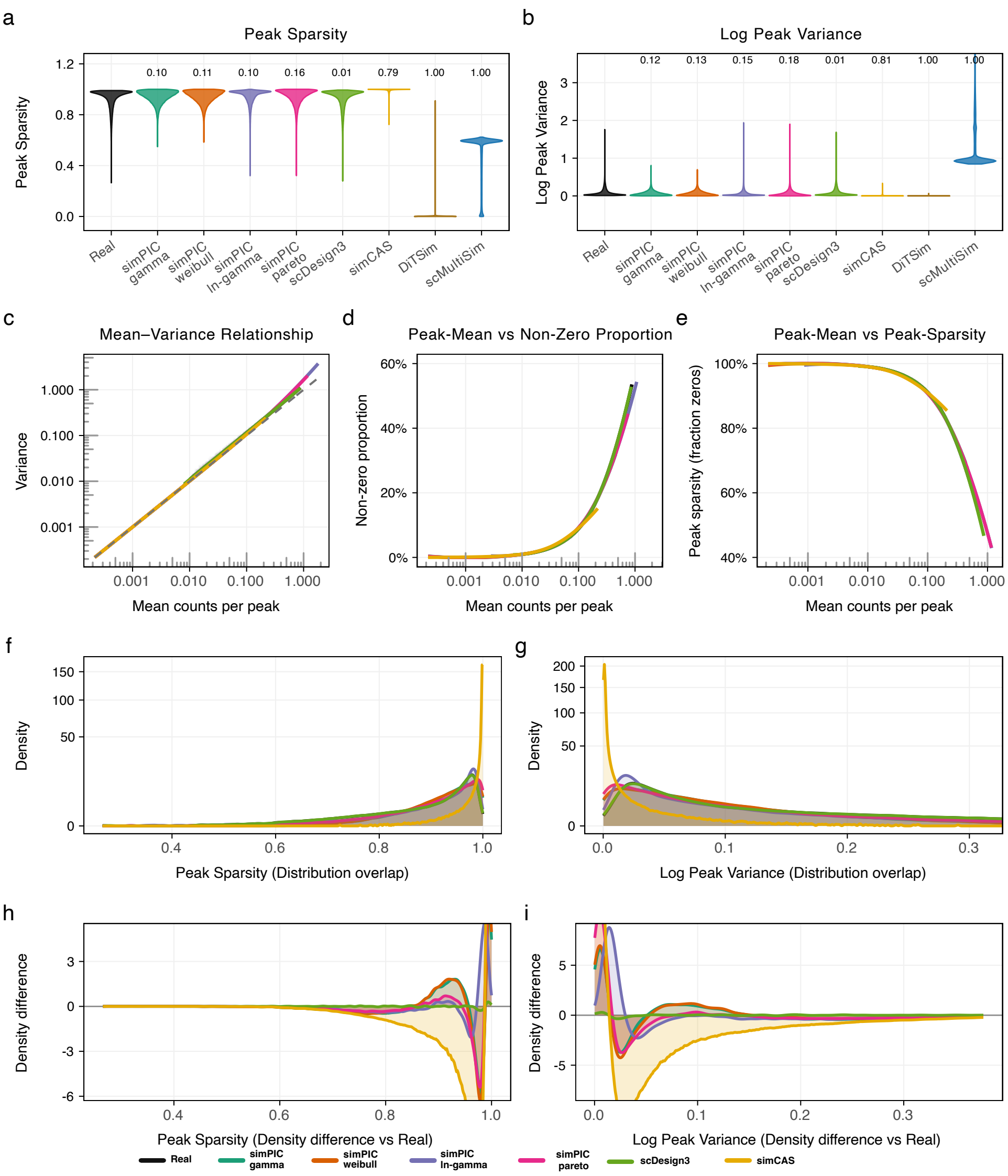

Evaluation metrics for additional datasets

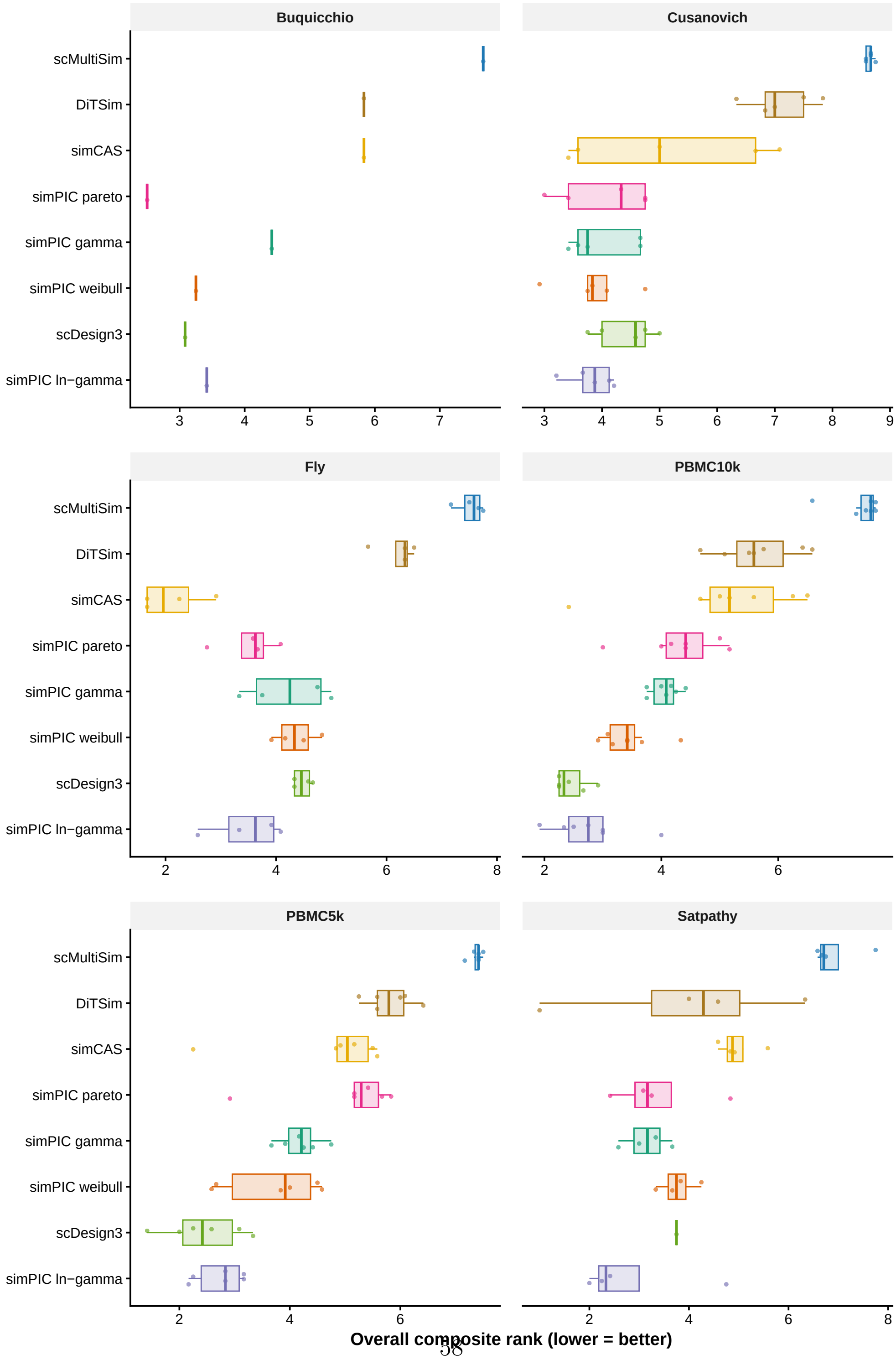

Fig. S4 Comparative boxplots for evaluation metrics MAD, MAE, RMSE and 1-PCC across library size, peak means and cell sparsity in PBMC5k, Cusanovich and Fly brain dataset for simPIC(g) in blue, simPIC(w) in green and simCAS in purple. Each point represents a cell-type.

simPIC simulates multiple cell types and batch effects with configurable group structure

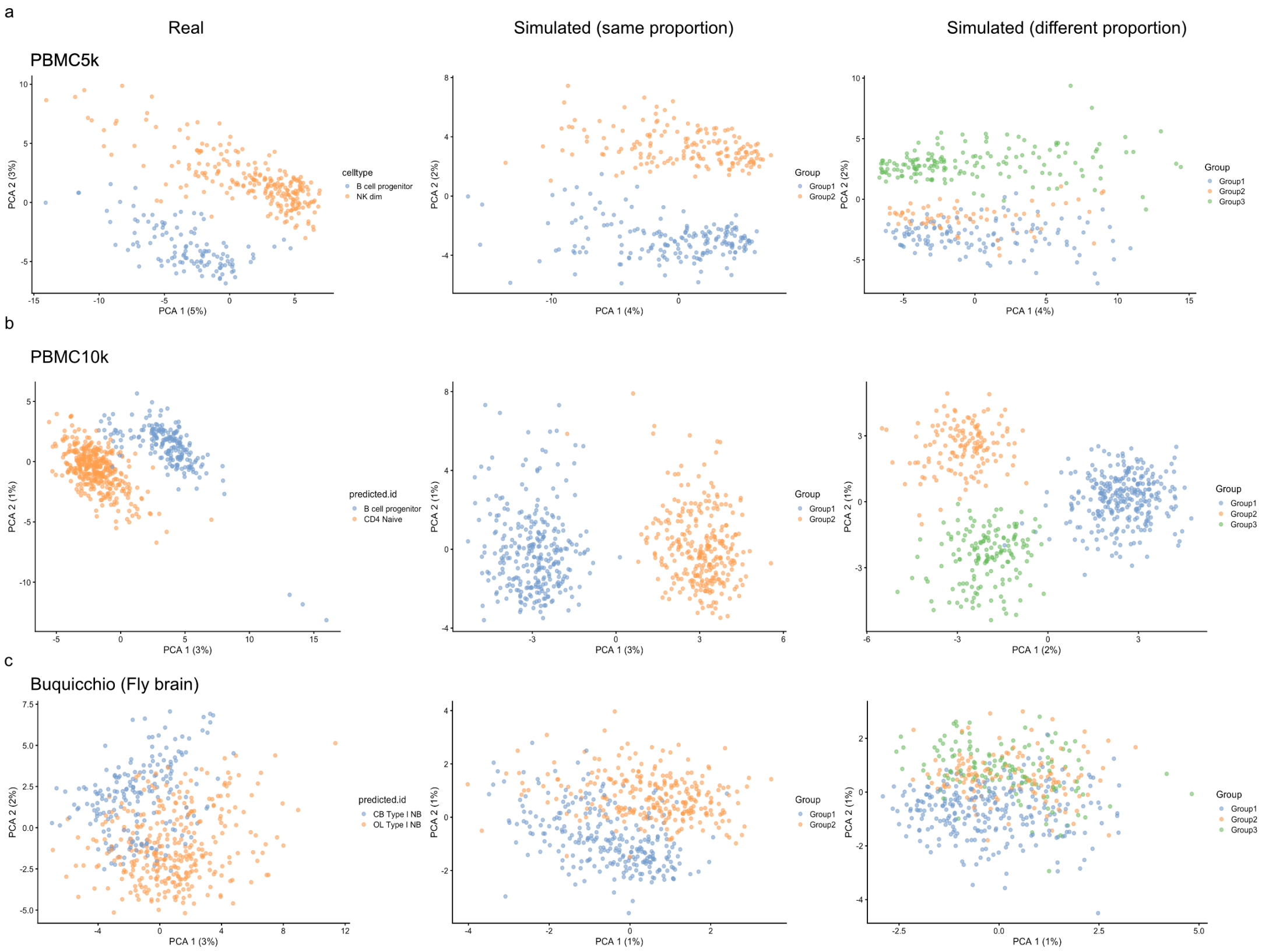

Fig. S5 Principal Component Analysis (PCA) plot illustrating the separation of two simulated cell type groups generated using simPIC. Group 1 is represented in blue, and Group 2 in orange, demonstrating that simPIC can simulate multiple cell types while maintaining the same or different proportions across groups.

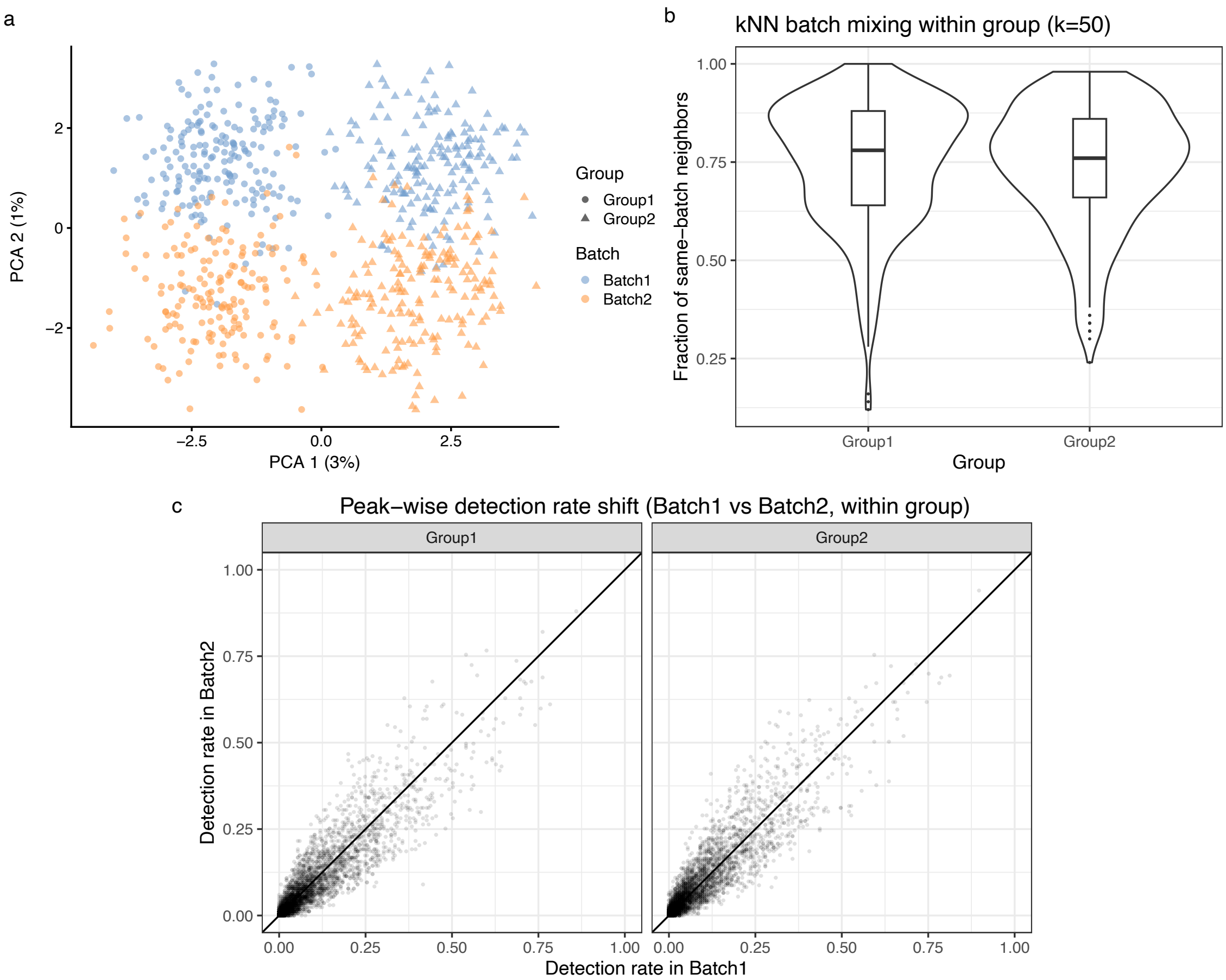

Fig. S6 a)PCA of simulated cells showing preservation of biological group structure (shapes) while introducing technical batch variation (colours). Batch effects are generated using peak-wise, batch-specific multiplicative factors applied to all cells within each batch.b) kNN batch-mixing (k = 50) quantifies within-group batch-associated structure as the fraction of same-batch neighbours (higher values indicate stronger batch separation).c) Peak-wise detection-rate shift shows per-peak detection rates in Batch1 versus Batch2 within each group, with the diagonal indicating no batch effect; systematic deviations reflect feature-level batch shifts consistent with the multiplicative batch model

Simulating population scale data with genetic effects

Clustering diagnostics were done for multiple libraries across the Alzheimer's disease (AD) and non Alzheimer's category. The following figures show all the different examples with varying no of cells for each library

The below figures are for earlyAD samples

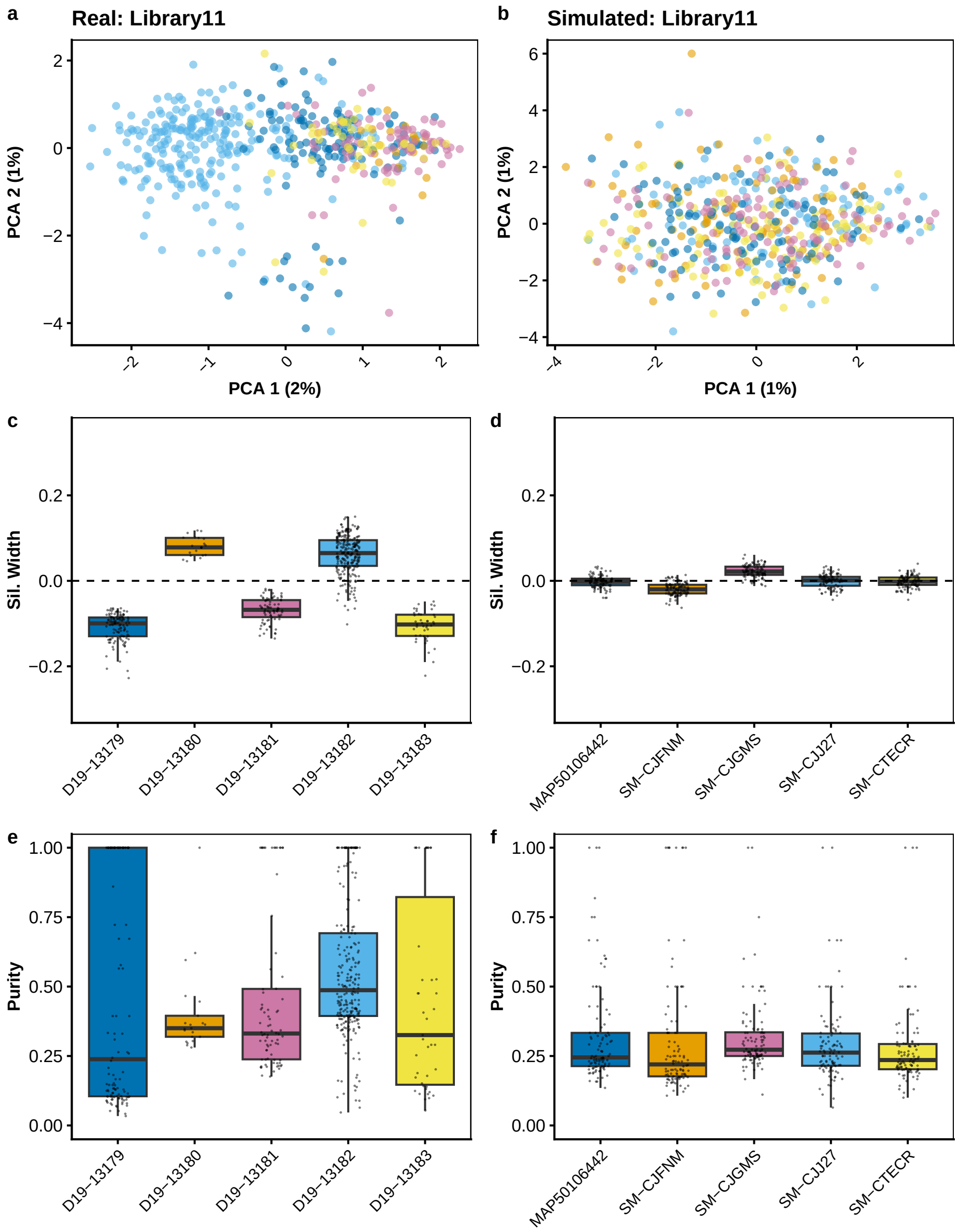

Fig. S7 Quantifying clustering in real and simPIC-simulated data for early AD library 11 with 1693 peaks and 507 cells. (Top row) PCA plot of real and simulated data, coloured by individual, showing the preservation of individual-specific structure. (Middle row) Silhouette width comparison between real and simulated data, assessing cluster compactness. (Bottom row) Neighbourhood purity analysis, evaluating local structure consistency across real and simulated datasets.

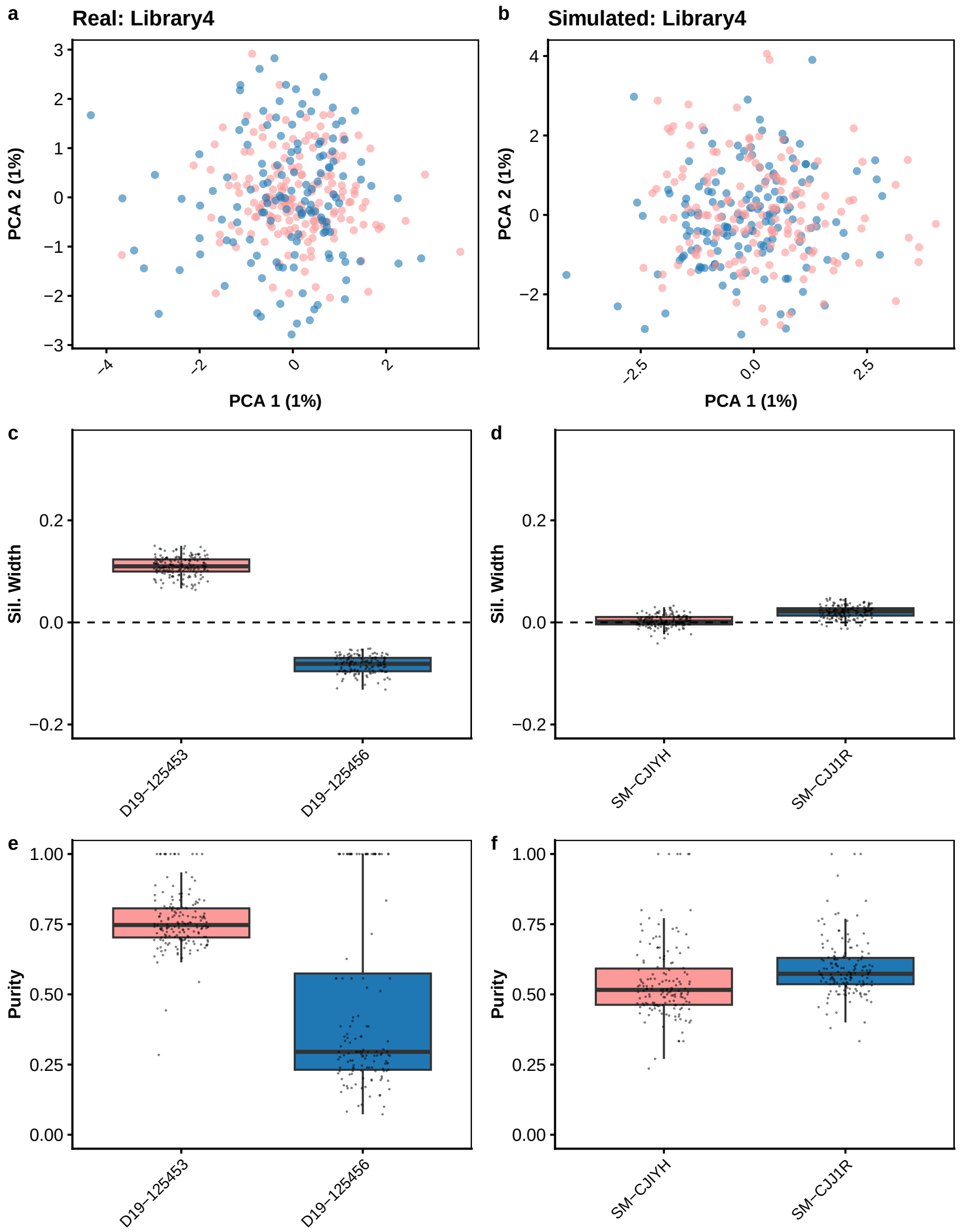

Fig. S8 Quantifying clustering in real and simPIC-simulated data for early AD library 4 with 1693 peaks and 307 cells. (Top row) PCA plot of real and simulated data, coloured by individual, showing the preservation of individual-specific structure. (Middle row) Silhouette width comparison between real and simulated data, assessing cluster compactness. (Bottom row) Neighbourhood purity analysis, evaluating local structure consistency across real and simulated datasets.

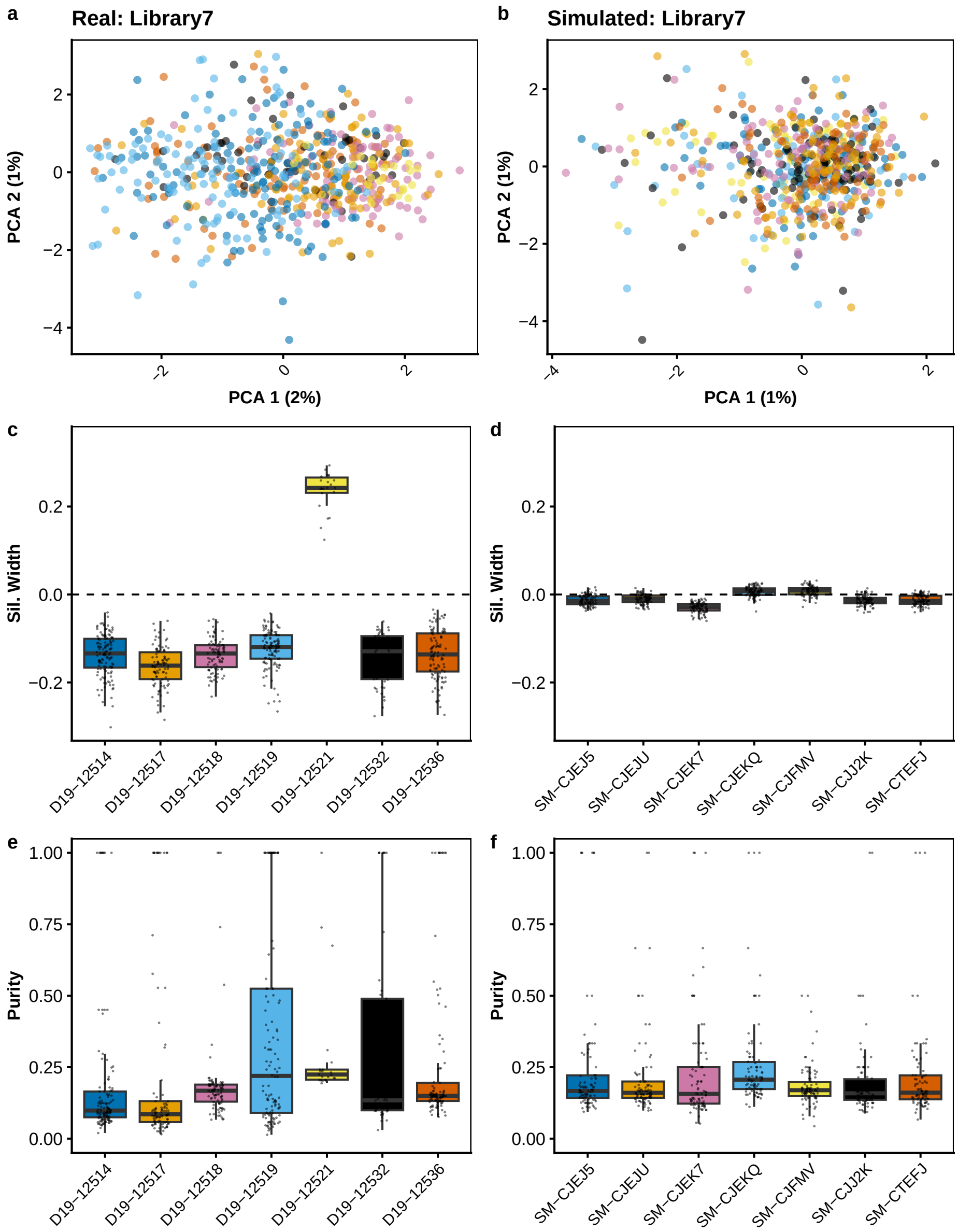

Fig. S9 Quantifying clustering in real and simPIC-simulated data for early AD library 7 with 1693 peaks and 604 cells. (Top row) PCA plot of real and simulated data, coloured by individual, showing the preservation of individual-specific structure. (Middle row) Silhouette width comparison between real and simulated data, assessing cluster compactness. (Bottom row) Neighbourhood purity analysis, evaluating local structure consistency across real and simulated datasets.

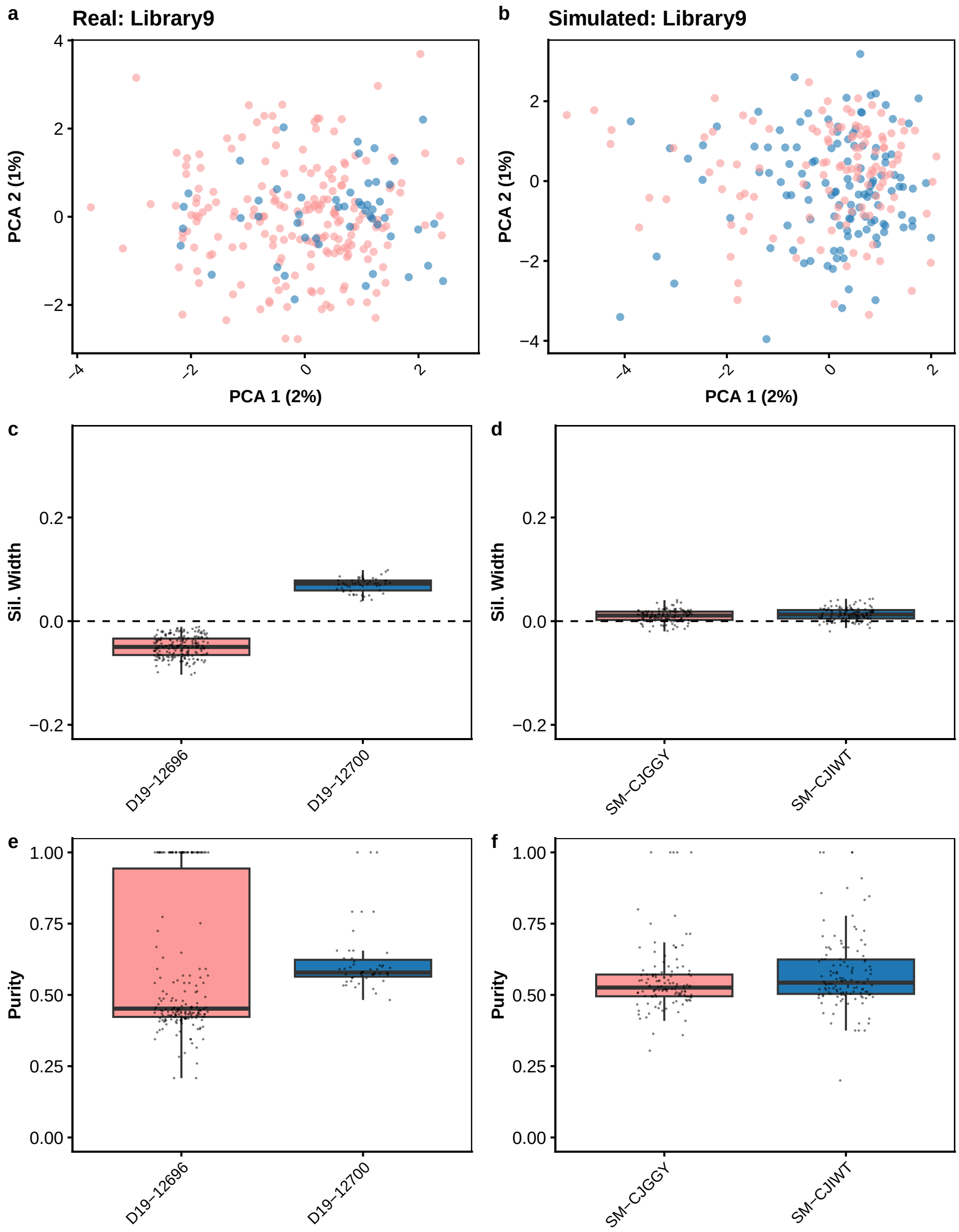

Fig. S10 Quantifying clustering in real and simPIC-simulated data for early AD library 9 with 1693 peaks and 244 cells. (Top row) PCA plot of real and simulated data, coloured by individual, showing the preservation of individual-specific structure. (Middle row) Silhouette width comparison between real and simulated data, assessing cluster compactness. (Bottom row) Neighbourhood purity analysis, evaluating local structure consistency across real and simulated datasets.

The below figures are for nonAD samples

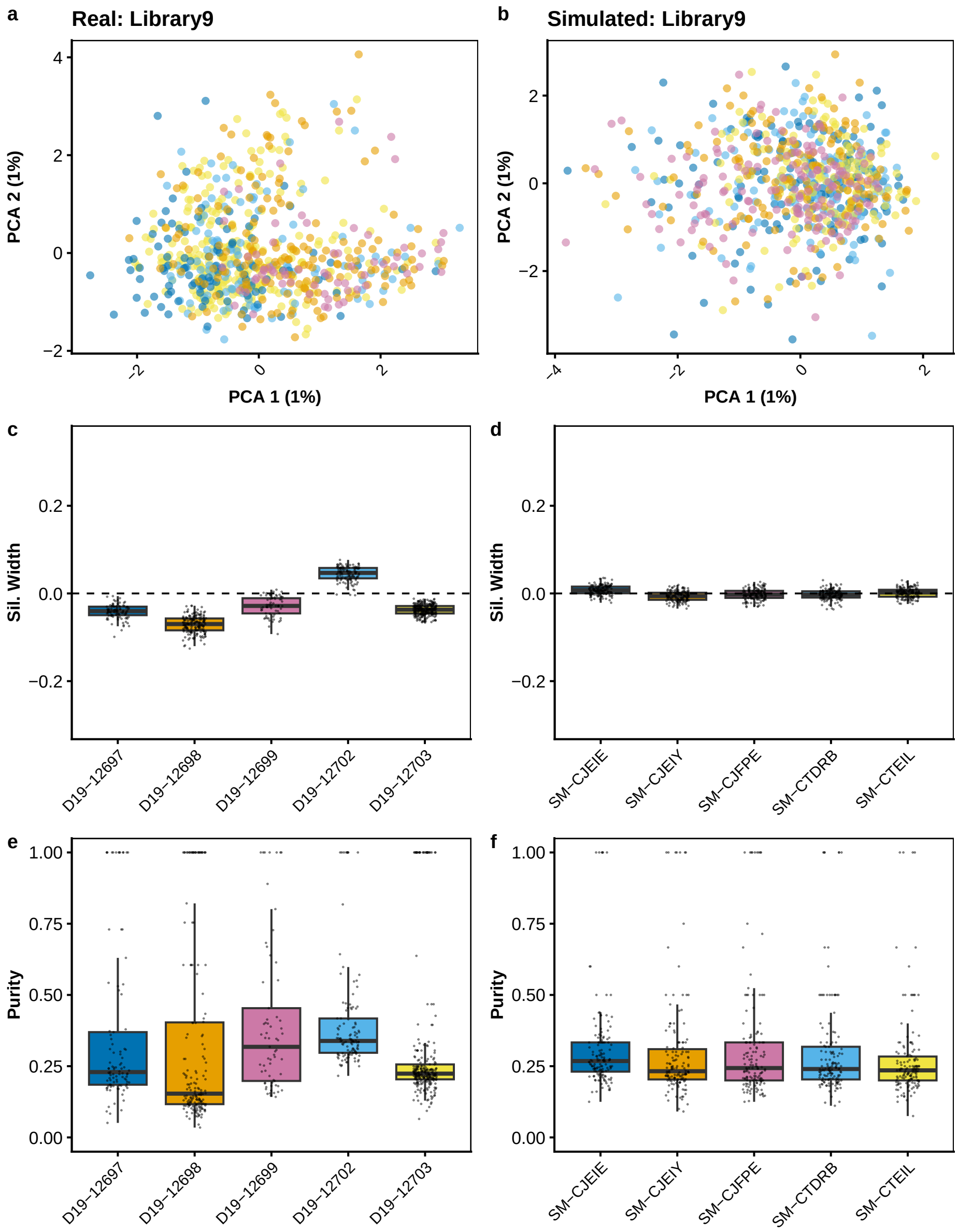

Fig. S11 Quantifying clustering in real and simPIC-simulated data for non AD library 9 with 1693 peaks and 710 cells. (Top row) PCA plot of real and simulated data, coloured by individual, showing the preservation of individual-specific structure. (Middle row) Silhouette width comparison between real and simulated data, assessing cluster compactness. (Bottom row) Neighbourhood purity analysis, evaluating local structure consistency across real and simulated datasets.

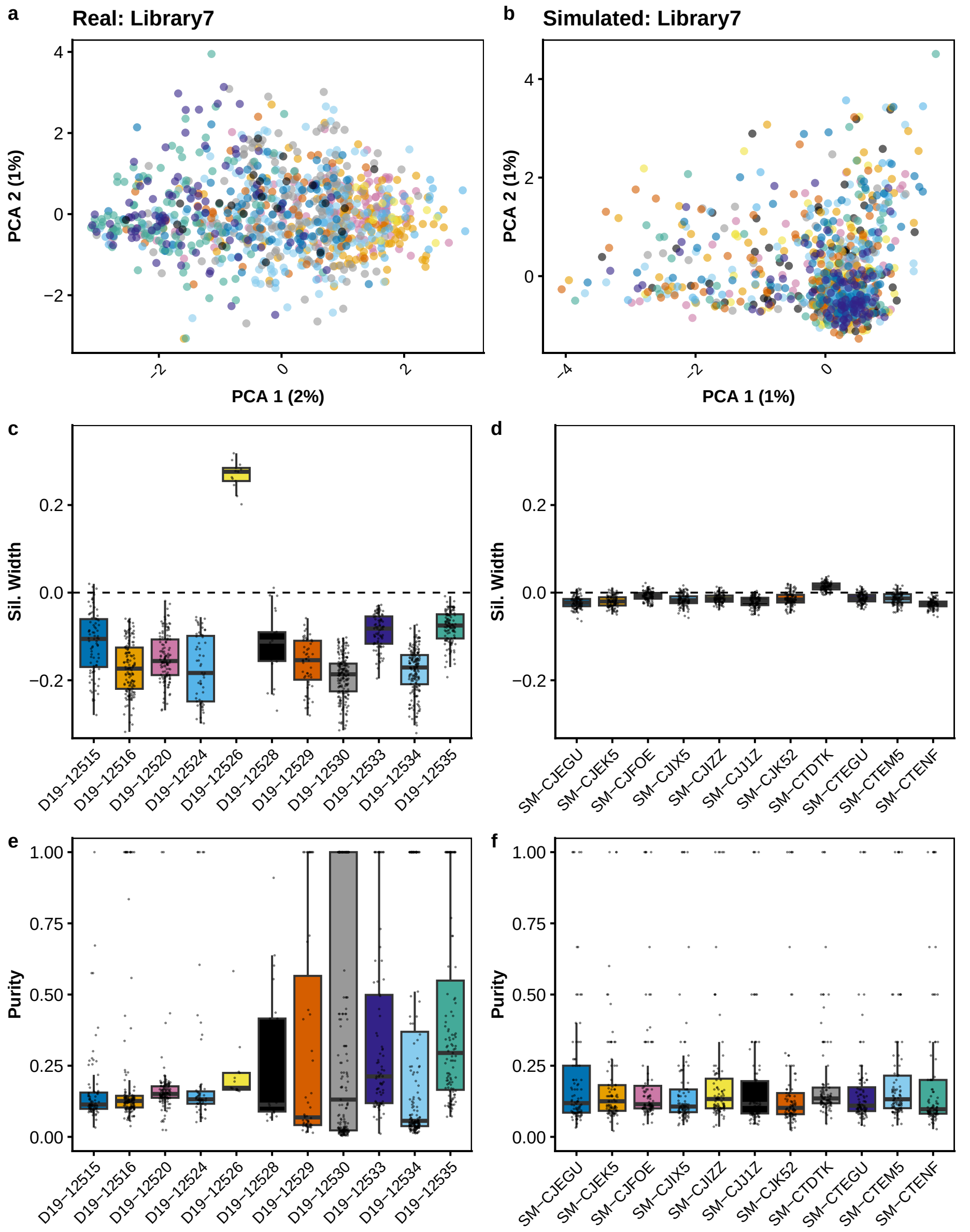

Fig. S12 Quantifying clustering in real and simPIC-simulated data for non AD library 7 with 1693 peaks and 1012 cells. (Top row) PCA plot of real and simulated data, coloured by individual, showing the preservation of individual-specific structure. (Middle row) Silhouette width comparison between real and simulated data, assessing cluster compactness. (Bottom row) Neighbourhood purity analysis, evaluating local structure consistency across real and simulated datasets.

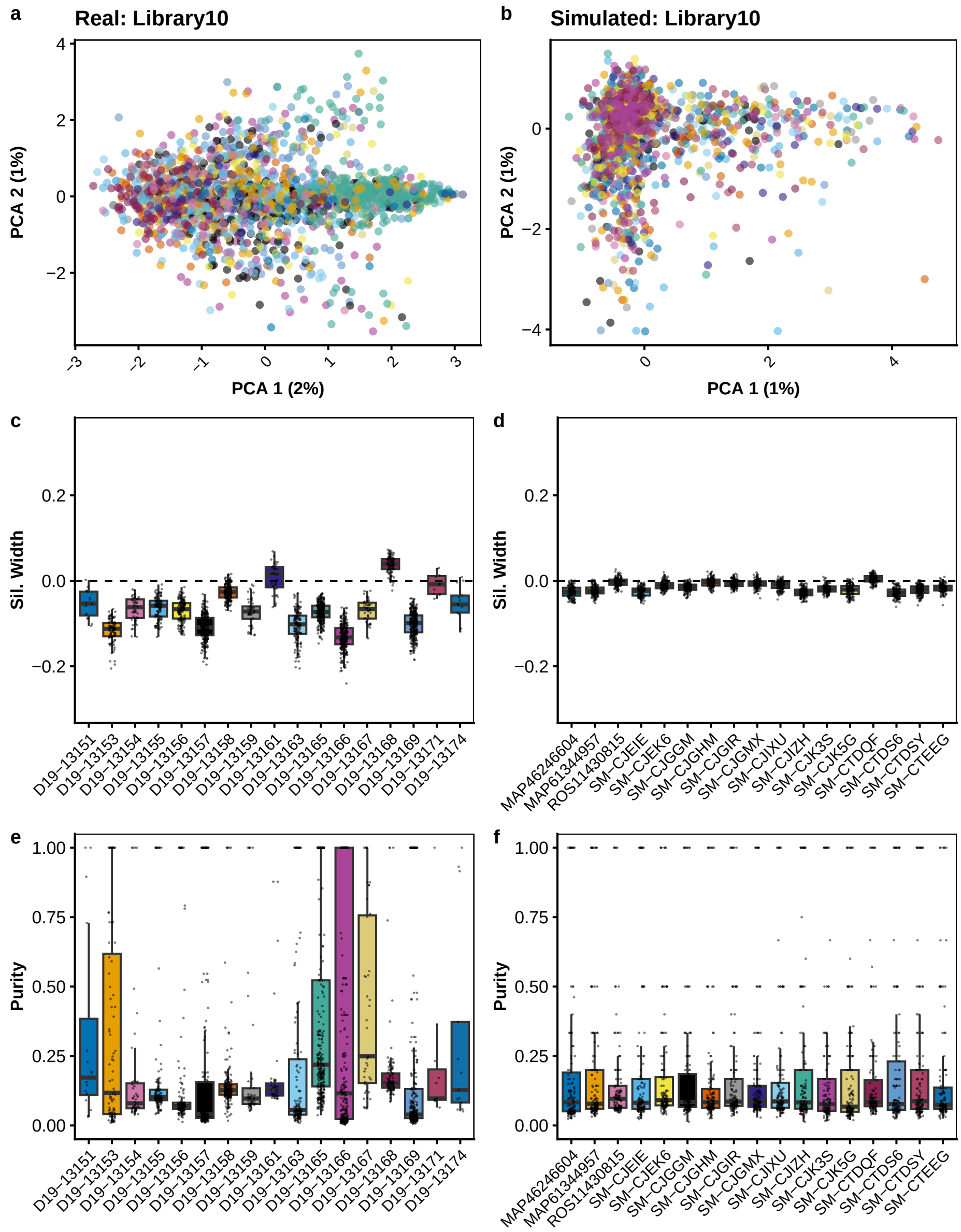

Fig. S13 Quantifying clustering in real and simPIC-simulated data for non AD library 10 with 1693 peaks and 2057 cells. (Top row) PCA plot of real and simulated data, coloured by individual, showing the preservation of individual-specific structure. (Middle row) Silhouette width comparison between real and simulated data, assessing cluster compactness. (Bottom row) Neighbourhood purity analysis, evaluating local structure consistency across real and simulated datasets.

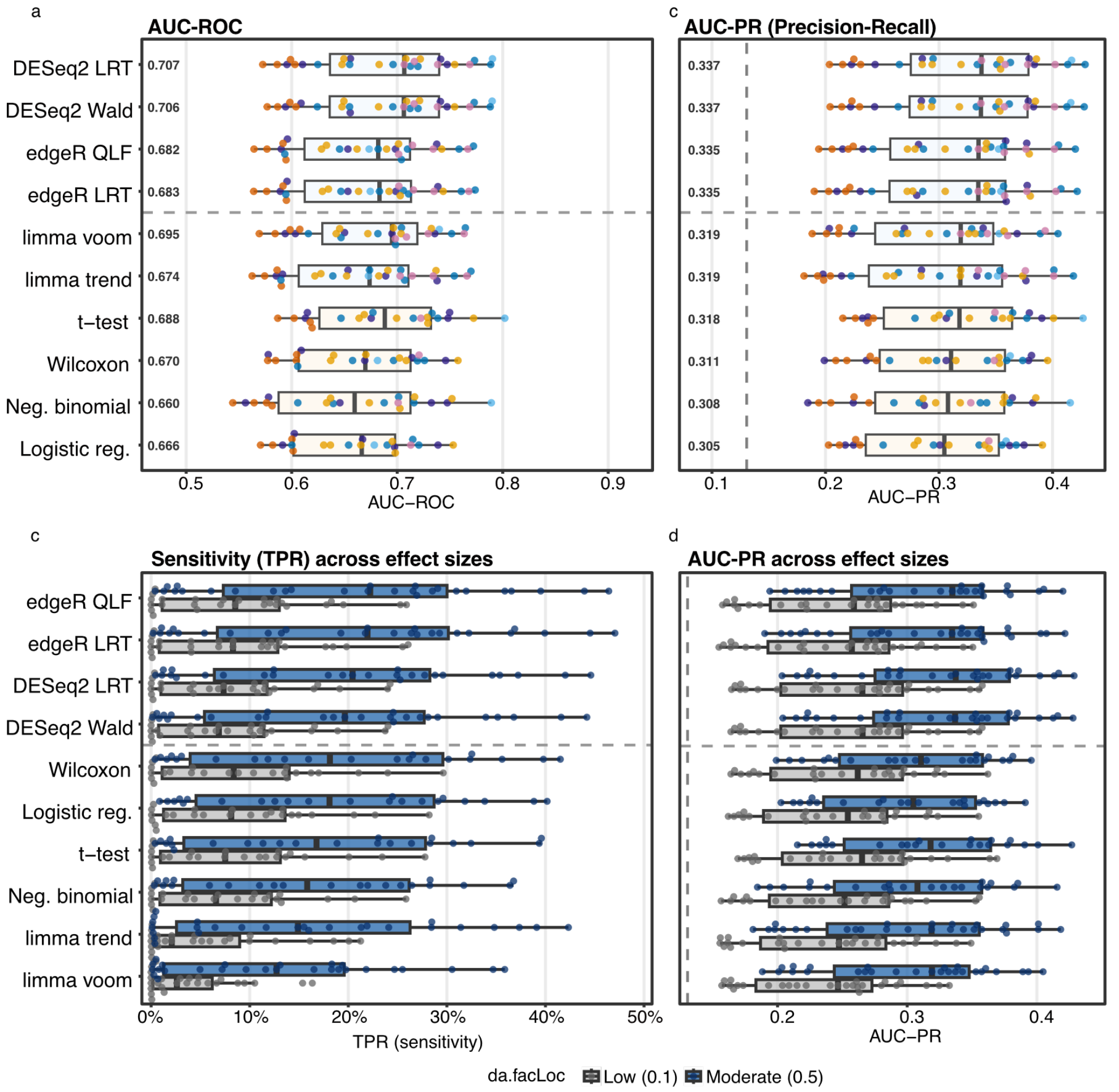

Fig. S14 Performance of differential accessibility methods across simPIC simulated scATAC-seq datasets. (a) Area under the receiver operating characteristic curve (AUC–ROC) for each method across simulated datasets, summarising overall discrimination performance. (b) Area under the precision–recall curve (AUC–PR), highlighting performance under class imbalance typical of differential accessibility analyses. (c) Sensitivity (true positive rate, TPR) across effect sizes, comparing detection power under low (0.1) and moderate (0.5) effect sizes.(d) AUC–PR stratified by effect size, illustrating method robustness to increasing signal strength.

References

[1] Zhao F, Ma X, Yao B, Lu Q, Chen L. scaDA: A novel statistical method for differential analysis of single-cell chromatin accessibility sequencing data. PLoS Comput Biol. 2024;20(8):e1011854.

[2] Zhang H, Mulqueen RM, Iannuzo N, Farrera DO, Polverino F, Galligan JJ, et al. txci-ATAC-seq: a massive-scale single-cell technique to profile chromatin

accessibility. Genome Biol. 2024;25(1):78.

[3] Robinson MD, McCarthy DJ, Smyth GK. edgeR: a Bioconductor package for differential expression analysis of digital gene expression data. Bioinformatics. 2010;26(1):139–140.

[4] Zappia L, Phipson B, Oshlack A. Splatter: simulation of single-cell RNA sequencing data. Genome Biol. 2017;18(1):174.
